# Cascade Annulation Strategy for Expeditious Assembly of Hydroxybenzo[*c*]chromen-6-ones and Their Photophysical Property Studies

**DOI:** 10.1021/acs.joc.3c02188

**Published:** 2023-11-18

**Authors:** Yanan Liu, Pui Ying Choy, Demao Wang, Mengdi Wu, Qiang Tang, Xinwei He, Yongjia Shang, Fuk Yee Kwong

**Affiliations:** † Key Laboratory of Functional Molecular Solids, Ministry of Education, Anhui Laboratory of Molecule-Based Materials (State Key Laboratory Cultivation Base), College of Chemistry and Materials Science, Anhui Normal University, Wuhu 241000, P. R. China; ‡ Department of Chemistry and State Key Laboratory of Synthetic Chemistry, The Chinese University of Hong Kong, New Territories, Shatin, Hong Kong, P. R. China

## Abstract

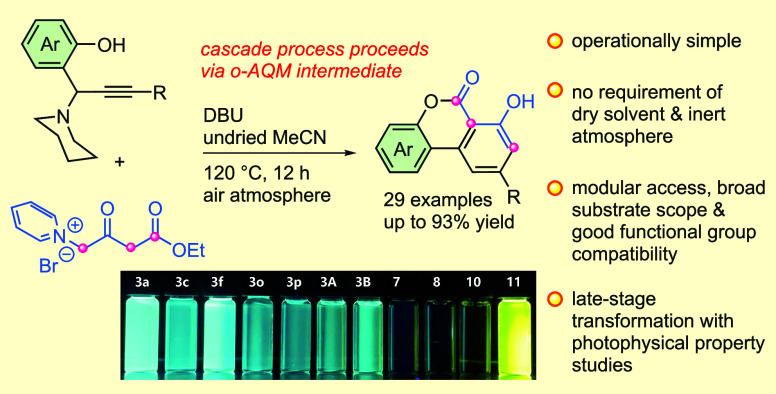

A 1,8-diazabicyclo[5.4.0]undec-7-ene-promoted cascade double-annulation of *ortho*-alkynyl quinone methide (in situ generated from modular propargylamine) for constructing of 2-aryl-4-hydroxybenzo[*c*]chromen-6-ones is developed. This synthetic strategy offers remarkable operational simplicity as it allows the use of benchtop-grade solvents without the need for predrying measures and inert atmosphere protection. Additionally, it demonstrates good functional group compatibility. The photophysical properties of these compounds were also examined, revealing bright fluorescence with high quantum yields.

## Introduction

**Scheme 1:**
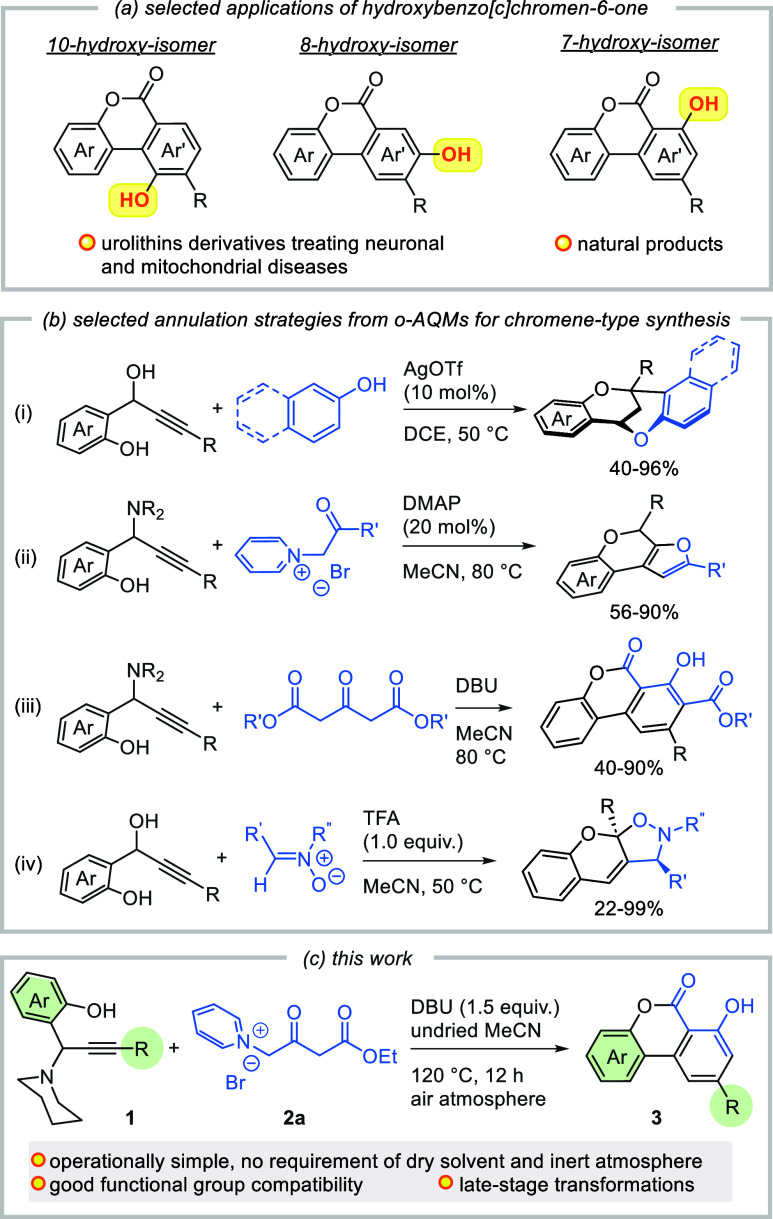
Selected Applications of Hydroxybenzo[*c*]chromen-6-one and Recent Selected Synthetic Strategies using *o*-AQMs for Chromene-type Synthesis

The benzo[*c*]chromen-6-one unit is widely recognized as one of the most important scaffolds embodied in various bioactive natural products^1^ and pharmaceutical lead molecules.^2^ In particular, benzo[*c*]chromen-6-one systems containing a hydroxyl group have proven to be biologically useful (Scheme 1a) and exhibit unique optical properties in photocatalysts and biosensors.^3^ Moreover, these systems can act as blue-green fluorescent dyes, characterized by high fluorescence quantum yields and large Stokes shifts.^4^ In recent years, several synthetic approaches have been developed to access and diversify this valuable framework.^5^ These include the Baeyer–Villiger oxidation of fluorenone,^6^ transition metal-catalyzed intra- or intermolecular coupling reactions,^7^ free-radical-mediated syntheses,^8^ and multicomponent strategies.^9^

Annulation of the *ortho*-alkynyl quinone methide (*o*-AQM)^10^ intermediate offers a versatile strategy for the rapid assembly of heterocyclic compounds, particularly for the synthesis of chromene-related systems (Scheme 1b).^11^ This approach allows for the efficient and streamlined construction of diverse structures within the chromene family. In 2018, Du and co-workers disclosed a versatile Lewis acid-catalyzed double annulation of *o*-AQM with electron-rich phenols for assembling synthetically and biologically interesting tetracyclic-bridged dioxabicyclo[3.3.1]nonane skeletons (Scheme 1b(i)).^12^ In 2020, a tandem 4-dimethylaminopyridine (DMAP)-catalyzed annulation reaction between propargylamines and acyl carbene surrogate for accessing furan-fused chromenes was reported (Scheme 1b(ii)).^13^ Later, lactonization/benzannulation of *o*-AQM (in situ generated from propargylamines) with dimethyl 3-oxoglutarate was described to achieve the functionalized benzo[*c*]chromen-6-one derivatives (Scheme 1b(iii)).^14^ Very recently, Liu established a Brønsted acid-catalyzed tandem 1,6-addition/double annulation protocol using *o*-hydroxyphenyl propargylic alcohols and nitrones to give densely functionalized chromeno[3,2-*d*]isoxazoles (Scheme 1b(iv)).^15^ In continuing our interest of investigating chromene^16^ and fused arene systems,^17^ we sought to adopt the *o*-AQM chemistry in a new cascade reaction between alkylaminophenols and 1-(4-ethoxy-2,4-dioxobutyl)pyridin-1-ium bromide, for modular assembly of hydroxybenzo[*c*]chromen-6-one with rich diversity of substitution patterns.

## Results and Discussion

To achieve our targeted hydroxybenzo[*c*]chromen-6-one, we began our attempts by investigating the reaction parameters favorable for this transformation using propargylamine **1a** and 1-(4-ethoxy-2,4-dioxobutyl)pyridin-1-ium bromide (**2a**) as the prototypical substrates (Table 1). Upon surveying common bases, 1,8-diazabicyclo[5.4.0]undec-7-ene (DBU) gave the best product yield of compound **3a** (entries 1–4). When the amine moiety of **1a** was replaced by piperidinyl, morpholinyl, tetrahydroisoquinolinyl, and benzimidazolyl groups, the desired product yields decreased significantly (entries 1 vs 7–10). *o*-Hydroxybenzyl alcohol was also tested in this reaction; only 35% product yield was obtained (entry 11). Other solvents such as tetrahydrofuran (THF), toluene, dimethylformamide (DMF), 1,4-dioxane, ethyl acetate (EtOAc), and dimethyl sulfoxide (DMSO) led to inferior yields (entries 5 vs 12–17). Lowering the reaction temperature or shortening the reaction time resulted in a drop of product yield (entries 5 vs 18–19).

**Table 1:**
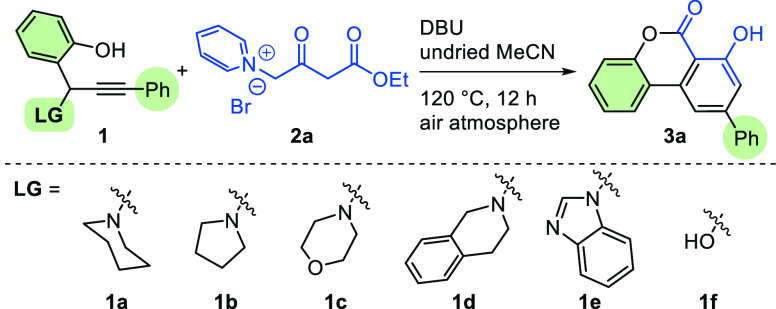
Evaluation of Reaction Parameters and Leaving Group Studies^a^

entry	substrate **1**	base (equiv)	solvent	% yield^b^
1	**1a**	DBU (1.0)	MeCN	53
2	**1a**	NaO*t*-Bu (1.0)	MeCN	21
3	**1a**	DABCO (1.0)	MeCN	n.r.
4	**1a**	K_2_CO_3_ (1.0)	MeCN	n.r.
5	**1a**	DBU (1.5)	MeCN	80
6	**1a**	DBU (1.75)	MeCN	60
7	**1b**	DBU (1.5)	MeCN	78
8	**1c**	DBU (1.5)	MeCN	45
9	**1d**	DBU (1.5)	MeCN	23
10	**1e**	DBU (1.5)	MeCN	n.r.
11	**1f**	DBU (1.5)	MeCN	35
12	**1a**	DBU (1.5)	toluene	n.r.
13	**1a**	DBU (1.5)	DMF	n.r.
14	**1a**	DBU (1.5)	dioxane	43
15	**1a**	DBU (1.5)	EtOAc	39
16	**1a**	DBU (1.5)	DMSO	39
17	**1a**	DBU (1.5)	THF	40
18^c^	**1a**	DBU (1.5)	MeCN	52
19^d^	**1a**	DBU (1.5)	MeCN	50
20^e^	**1a**	DBU (1.5)	MeCN	69

a Reaction conditions: **1a** (0.1 mmol), **2a** (0.1 mmol), base (as indicated), and undried solvent (2.0 mL) were stirred at 120 °C under an air atmosphere for 12 h. n.r. = no reaction. DBU = 1,8-diazabicyclo(5.4.0)undec-7-ene. DABCO = 1,4-diazabicyclo[2.2.2]octane.

b Isolated yields were reported.

c 90 °C was used.

d For 9 h.

e 1.0 mL solvent was used.

**Scheme 2:**
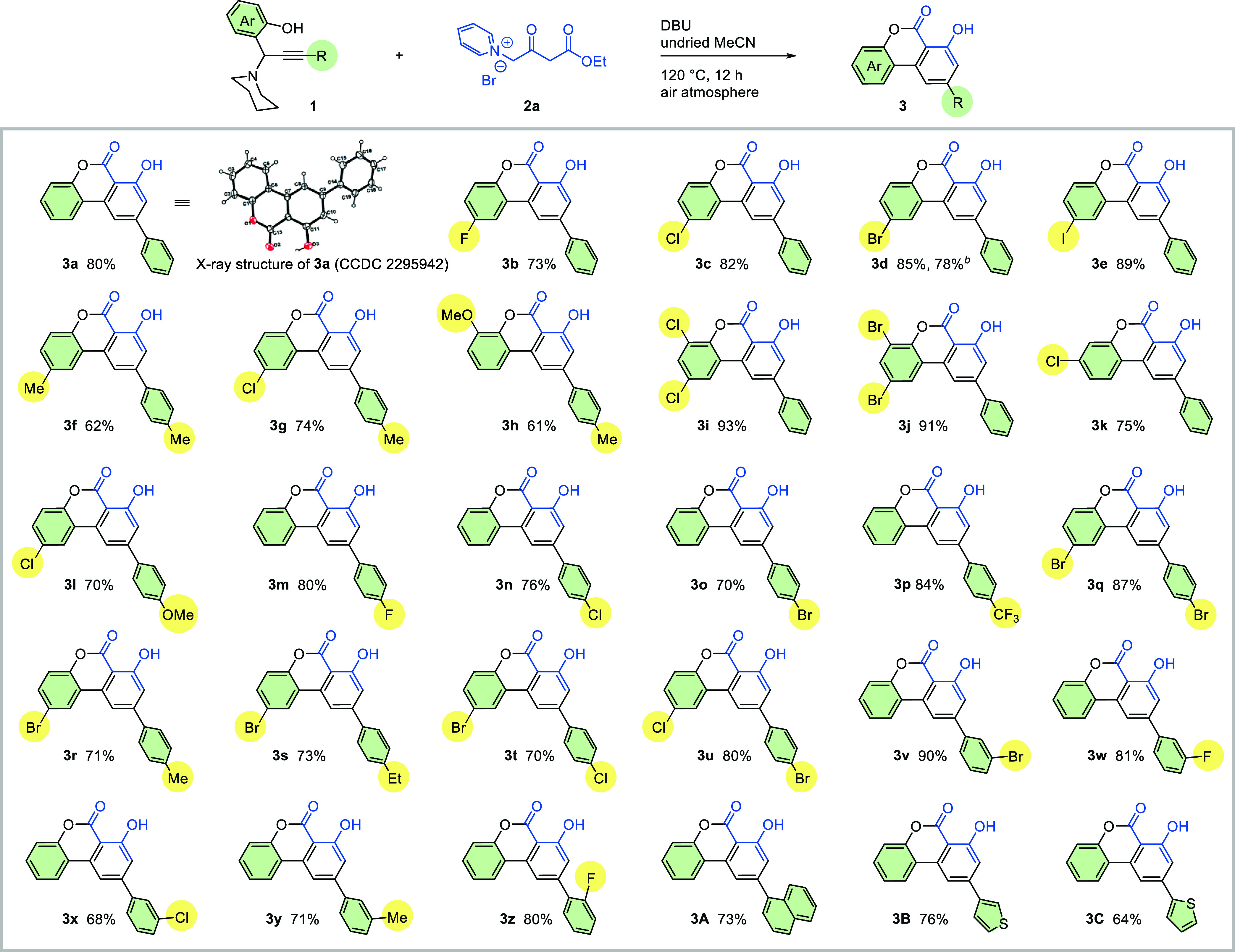
Substrate Scope Reaction conditions: propargylamines **1** (0.2 mmol), 1-(4-ethoxy-2,4-dioxobutyl)pyridin-1-ium bromide (**2a**) (0.2 mmol), DBU (0.3 mmol.) in undried MeCN (4.0 mL) at 120 °C under an air atmosphere for 12 h. Isolated yields were reported. ^b^Gram-scale synthesis (5 mmol of **1** was used, and 1.42 g of product **3d** was delivered).

With the identified reaction conditions in hand, we then turned our attention to examine the substrate scope (Scheme 2). In general, the reaction proceeded smoothly with a broad spectrum of propargylamines. Electron-neutral (products **3a**, **3f**, and **3y**), -rich (product **3h**), and -deficient (products **3b**, **3m**, **3p**, **3w**, and **3z**) propargylamines were well-tolerated. The structure of **3a** was unambiguously characterized by single-crystal X-ray analysis (CCDC 2295942, see Supporting Information for details). It is notable to show that halogen groups, e.g., −I, −Br, and −Cl groups, located at either the phenolic arene or the alkynyl arene, remained intact under these reaction conditions (products **3c**–**3e**, **3g**, **3k**, **3l**, **3n**, **3o**, **3r**, **3s**, **3v**, **3x**). This beneficial outcome offers us an excellent opportunity for further transformation using cross-coupling protocols.^18^ Gratifyingly, dihalide substituents proved to be compatible under the reaction conditions (products **3i**, **3j**, **3q**). In particular, products **3t** and **3u** containing both bromo- and chloro-group are applicable for complementarily chemoselective coupling reaction.^19^ Sterically congested naphthyl- and heterocyclic thiophenyl-substituted substrates proceeded smoothly to furnish the desired products **3A**-**3C** in 73, 76, and 64% yields, respectively. To demonstrate the practicability and synthetic utility of the current protocol, a gram-scale experiment was also conducted, and 1.42 g of the product **3d** was successfully obtained.

**Scheme 3:**
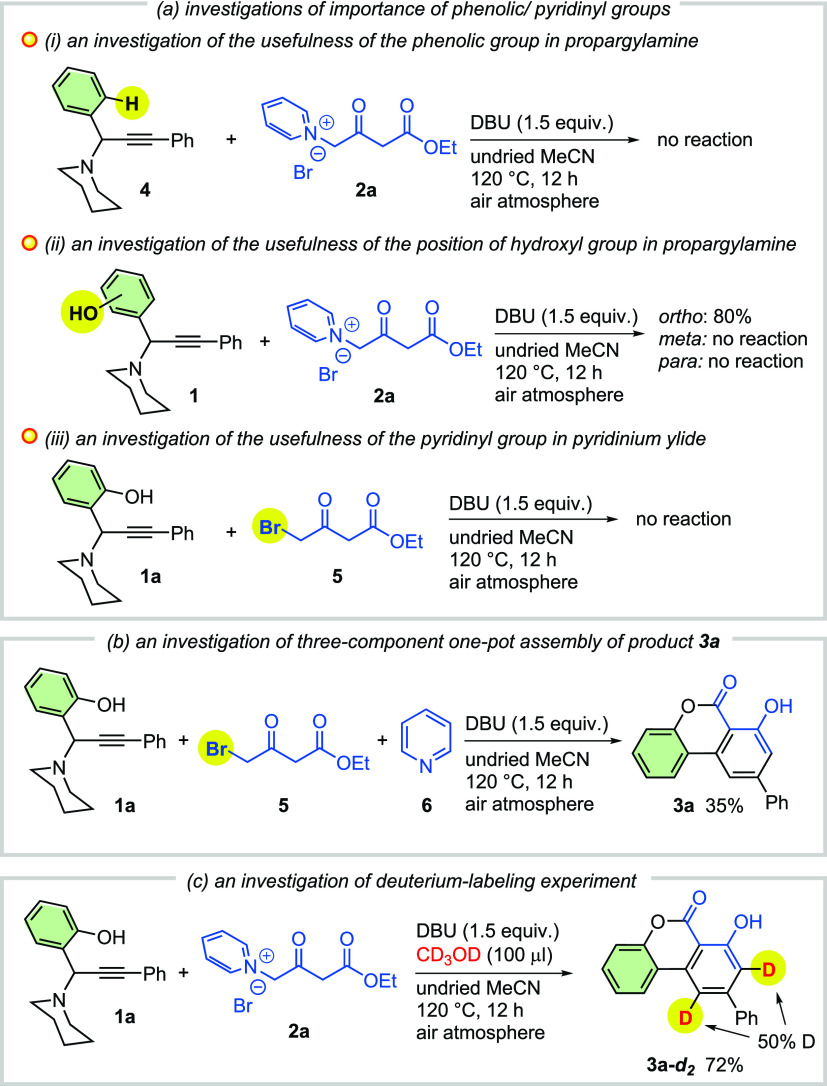
Control Experiments

To gain insight into the reaction mechanism, several control experiments were carried out (Scheme 3). Propargylamine **4** without hydroxyl group did not afford the desired product under the standard conditions, which implies that the hydroxyl group is essential for facilitating the reaction (Scheme 3a(i)). Similarly, no desired product was observed by altering the hydroxyl group from the *ortho-* to the *meta*- or *para*-position on the propargylamine (Scheme 3a(ii)), which demonstrated that the *ortho-*OH group is crucial to initiate the reaction through in situ generation of the *o*-AQM key intermediate. The cascade reaction did not proceed when propargylamine **1a** was treated with ethyl 4-bromo-3-oxobutanoate **5** (Scheme 3a(iii)). Furthermore, a direct three-component assembly for attaining the product **3a** was tested and the intended product was afforded in 35% yield only (Scheme 3b). A deuterium-labeling experiment with the addition of methanol-*d*_*4*_ was performed, and the deuterium-labeled product **3a**-*d*_*2*_ was isolated in 72% yield (Scheme 3c).

**Scheme 4:**
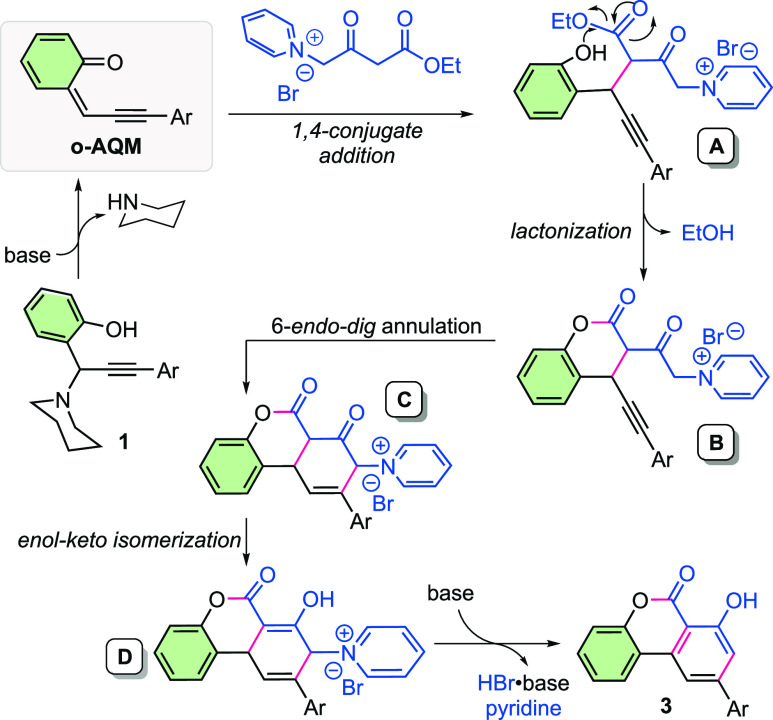
Plausible Mechanism

A mechanistic proposal for the reaction is illustrated in Scheme 4. The cascade reaction begins with the in situ generation of *o*-AQM in the presence of DBU, as suggested in the literature.^20^ 1,4-Conjugate addition of pyridinium methylide **2a** to *o*-AQM proceeds to give intermediate **A**, which then undergoes lactonization to produce the intermediate **B**. An intramolecular 6-*endo-dig* annulation of intermediate **B** provides ring-closured intermediate **C**, followed by an enol–keto tautomerization to generate the intermediate **D**. A 1,3-*H* shift and subsequent β-H elimination of the intermediate **D** furnishes the desired product **3**.

**Scheme 5:**
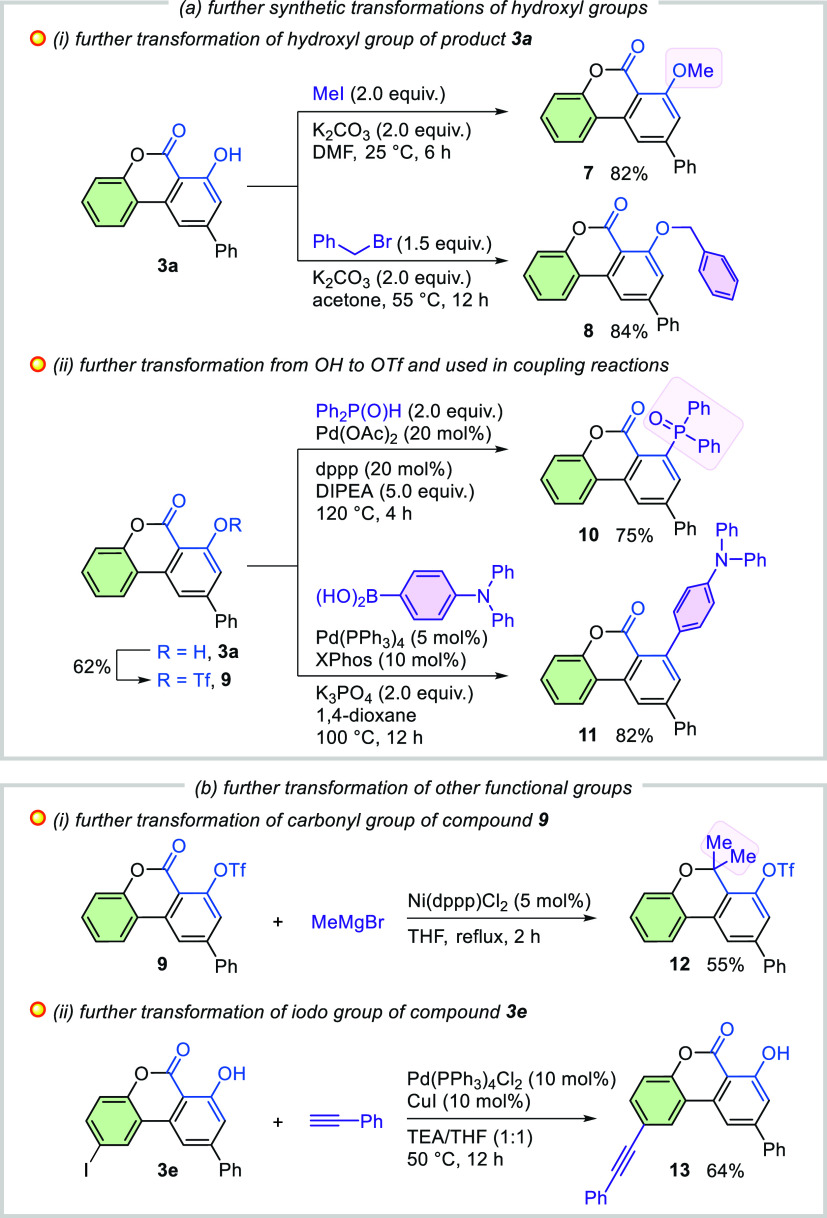
Further Synthetic Transformations

To showcase the versatility of the resulting products, we explored further transformations (Scheme 5). Alkylation and benzylation of the hydroxyl group of benzo[*c*]chromen-6-one **3a** were achieved in the presence of K_2_CO_3_, giving the corresponding products **7** and **8** in 82 and 84% yields, respectively (Scheme 5a(i)). Compound **3a** was converted to its Tf analogue **9**, which serves as a potential coupling partner in transition metal-catalyzed direct phosphorylation and arylation reactions (Scheme 5a(ii)). Furthermore, the treatment of compound **9** with methylmagnesium bromide also proceeded smoothly, resulting in the formation of the corresponding 6,6-dimethyl-6*H*-benzo[*c*]chromene (**12**) (Scheme 5b(i)). Alkynyl-functionalized benzo[*c*]chromen-6-one **13** was synthesized by palladium-catalyzed Sonogashira coupling between iodo-substituted product **3e** and phenylacetylene (Scheme 5b(ii)).

**Figure 1:**
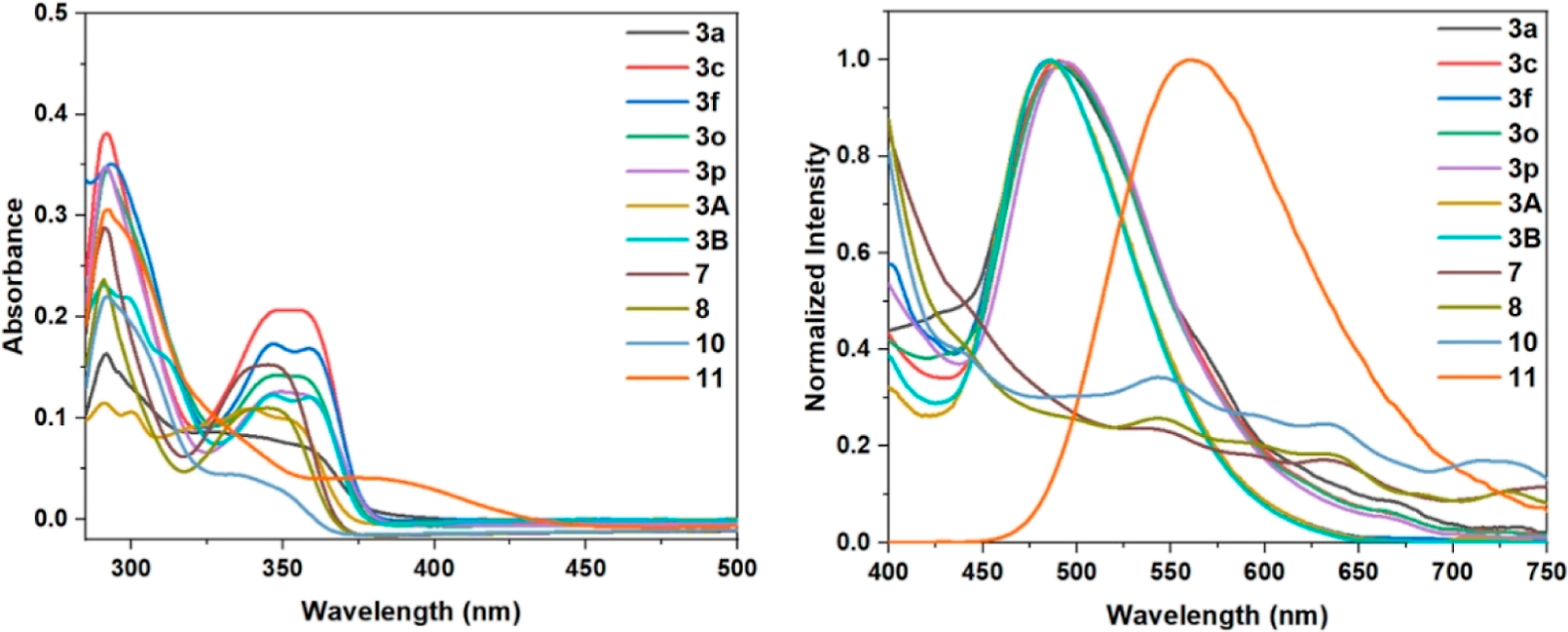
Fluorescence spectra of hydroxybenzo[*c*]chromen-6-ones **3** and their derivatives.

Following that, we proceeded to evaluate the physical characteristics of hydroxybenzo[*c*]chromen-6-ones **3** and their derivatives. It was observed that almost all of these compounds exhibited photoluminescence within the visible range (Figure 1). The photophysical property data are depicted in Table 2. The synthesized hydroxybenzo[*c*]chromen-6-ones **3** exhibit UV/vis absorption maxima in the 349–356 nm range in dichloromethane, with fluorescence emission maxima in the 488–494 nm range and Stokes shifts between 132 and 144 nm. The fluorescence quantum yields (Φ_F_) for the selected hydroxybenzo[*c*]chromen-6-ones **3** in dichloromethane were determined with the range 18.6–33.8%. The derivatives **7**, **8**, and **10** exhibited less fluorescence properties when the free OH group of **3a** was either protected or replaced, which demonstrated that the intermolecular H-bond plays an important role in the photophysical properties. Compound **11** showed an obvious red shift of the fluorescence maxima with the highest fluorescence quantum yield of 44.1%, which may be attributed to the aggregation-caused quenching (ACQ) and twisted intramolecular charge transfer (TICT) process enhanced by the π-extended groups on the arene moiety.^21^ These results indicated that such a class of fluorescent benzo[*c*]chromen-6-ones exhibits potential applications in the field of biology as fluorescent probes.

**Table 2:**
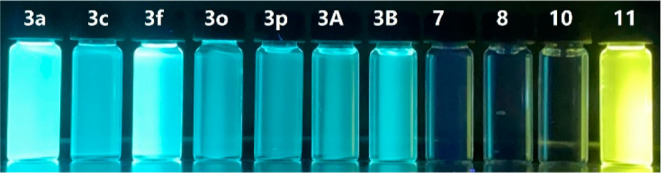
Photophysical Properties of Hydroxybenzo[*c*]chromen-6-ones **3** and Their Derivatives from Further Transformations

entry	product	λ_abs_ (nm)^a^	ε (L·mol^–^^1^·cm^–^^1^)^b^	λ_em_ (nm)^c^	Stokes shift (nm)	Φ_*F*_ (%)^d^
1	**3a**	356	3650	488	132	31.4
2	**3c**	353	10,250	488	135	26.6
3	**3f**	353	8277	483	130	26.4
4	**3o**	345	7150	494	149	33.8
5	**3p**	348	6350	492	144	18.6
6	**3A**	347	5030	485	138	20.0
7	**3B**	352	5873	485	133	19.8
8	**7**	346	7450			3.4
9	**8**	345	5400			0.7
10	**10**	334	2250			5.3
11	**11**	378	2050	560	182	44.1

a Wavelength in absorption spectra were measured in dichloromethane solutions (ca. 2 × 10^–5^ M) at room temperature.

b Refers to the molar extinction coefficient.

c Wavelength in emission spectra were recorded at λ_em_ = 350 nm.

d Fluorescence quantum yields (Φ_F_) were measured using integrating sphere (excited at 380 nm).

In conclusion, we have successfully developed an efficient and operationally simple method for modular assembly of hydroxybenzo[*c*]chromen-6-ones. This cascade reaction between propargylamines and pyridinium ylide offers notable advantages, including the use of benchtop-grade solvents and the absence of inert atmosphere protection. This method demonstrates excellent functional group compatibility and allows for easy manipulation of the substitution pattern, as shown by the synthesis of 29 examples with product yields of up to 93%. It is worth noting that this protocol shows favorable tolerance toward halo groups (–I, –Br, –Cl) located at various positions on the arene. This is particularly significant as it overcomes limitations observed in existing transition metal-catalyzed ring-closure processes for generating the chromene-type skeleton. Additionally, we demonstrated that the retained halo groups provide an excellent opportunity for further functionalization using modern cross-coupling technologies such as the Suzuki–Miyaura reaction, Sonogashira coupling, and aromatic C–P bond formation. This further expands the synthetic possibilities and potential applications of the hydroxybenzo[*c*]chromen-6-one compounds synthesized through this method. An array of products **3** display strong fluorescence in various regions of the visible range, accompanied by a significant Stokes shift of 132–182 nm. Notably, the advantage of the present protocol lies in its ability to easily incorporate additional arene moieties, leading to the generation of compound **11**, which exhibits distinct photophysical properties. We anticipate that the double annulation strategy presented here offers rich potential for future applications as fluorescent probes in chemical biology investigations.

## Experimental Section

### General Information

Unless otherwise noted, all reagents were purchased from commercial suppliers and used without purification. Starting materials propargylamines **1** were synthesized using literature procedures.^22^ All cascade reactions were performed in a resealable screw-capped Schlenk flask (approximately 25 mL volume) in the presence of a Teflon-coated magnetic stirrer bar (4 mm × 10 mm). The solvents were used directly without purification, unless stated. Thin-layer chromatography (TLC) was performed on precoated silica gel 60 F_254_ plates. Silica gel (200–300 mesh) was used for column chromatography. Melting points were recorded on an uncorrected Melting Point instrument. The ^1^H and ^13^C NMR spectra were recorded on 400 and 100 MHz NMR spectrometers, unless otherwise specified. Chemical shifts (δ) in parts per million were reported relative to the residual signals of chloroform (7.26 ppm for ^1^H and 77.16 ppm for ^13^C), and all ^13^C NMR spectra were recorded with proton broadband decoupling and indicated as ^13^C{^1^H} NMR. Multiplicities are described as s (singlet), d (doublet), t (triplet), q (quartet), or m (multiplet), and the coupling constants (*J*) are reported in Hertz (Hz). High-resolution mass spectrometry (HRMS) analysis with a quadrupole time-of-flight mass spectrometer yielded ion mass/charge (*m*/*z*) ratios in atomic mass units.

### General Procedure for Synthesis of 1-(4-Ethoxy-2,4-dioxobutyl)pyridin-1-ium Bromide (Compound **2a**)

According to the literature,^23^ a mixture of pyridine (5.0 mmol, 395 mg), ethyl 4-bromoacetoacetate (5.0 mmol, 1045 mg), and diethyl ether (5 mL) was added to a round-bottom flask and stirred at room temperature for 5 h. Upon completion of the reaction, the reaction mixture was filtered to obtain the crude product, which was then washed with ethyl acetate three times and dried to give desired product **2a** as a white solid in 85% yield (1.22 g). ^1^H NMR (400 MHz, CDCl_3_): δ 9.27 (d, *J* = 5.6 Hz, 2H), 8.52 (t, *J* = 7.9 Hz, 1H), 8.09 (t, *J* = 7.1 Hz, 2H), 6.68 (s, 2H), 4.19 (t, *J* = 7.1 Hz, 2H), 4.02 (s, 2H), 1.28 (t, *J* = 7.1 Hz, 3H); ^13^C{1H} NMR (100 MHz, CDCl_3_): δ 194.4, 166.9, 146.5, 145.8, 127.9, 68.8, 62.2, 47.1, 14.2.

### General Procedure for Synthesis of Hydroxybenzo[*c*]chromen-6-ones **3**

A mixture of propargylamines **1** (0.2 mmol, 1.0 equiv), 1-(4-ethoxy-2,4-dioxobutyl)pyridin-1-ium bromide (**2a**) (0.2 mmol, 1.0 equiv), and DBU (0.3 mmol, 1.5 equiv) were added under air atmosphere to a resealable screw-capped Schlenk tube. Acetonitrile (4 mL) was then added. The tube was sealed with a Teflon-coated cap, and the resulting mixture was stirred in an oil bath preheated to 120 °C for 12 h (monitored by TLC). Upon completion of the reaction, the reaction mixture was cooled to room temperature, extracted with CH_2_Cl_2_ (3 × 10 mL), and washed with brine. The organic layers were combined, dried over Na_2_SO_4_, filtered, and then evaporated under a vacuum. The residue was purified using flash column chromatography with silica gel (200–300 mesh), using ethyl acetate and petroleum ether as the elution solvent to give desired products **3**.

### 7-Hydroxy-9-phenyl-6*H*-benzo[*c*]chromen-6-one (Compound **3a**)^5e^

This compound was purified by column chromatography (ethyl acetate/petroleum ether = 1:10, *R*_*f*_ = 0.6) to afford a white solid in 80% yield (46 mg); mp = 173–174 °C; ^1^H NMR (400 MHz, CDCl_3_): δ 11.40 (s, 1H), 8.12 (d, *J* = 8.0 Hz, 1H), 7.79 (s, 1H), 7.70 (d, *J* = 7.2 Hz, 2H), 7.54–7.47 (m, 4H), 7.37 (t, *J* = 8.4 Hz, 2H), 7.30 (s, 1H); ^13^C{^1^H} NMR (100 MHz, CDCl_3_): δ 165.5, 162.7, 150.9, 150.4, 139.6, 135.6, 130.8, 129.2, 129.1, 127.5, 125.3, 123.5, 118.5, 117.9, 115.1, 111.2, 105.0; HRMS (electrospray ionization time-of-flight ESI-TOF) *m*/*z*: [M + H]^+^ calcd for C_19_H_13_O_3_, 289.0859; found, 289.0862.

### 2-Fluoro-7-hydroxy-9-phenyl-6*H*-benzo[*c*]chromen-6-one (Compound **3b**)

This compound was purified by column chromatography (ethyl acetate/petroleum ether = 1:10, *R*_*f*_ = 0.6) to afford a white solid in 73% yield (45 mg); mp = 191–192 °C; ^1^H NMR (400 MHz, CDCl_3_): δ 11.36 (s, 1H), 7.76 (dd, *J* = 9.1, 2.8 Hz, 1H), 7.69 (s, 1H), 7.68–7.66 (m, 2H), 7.50 (dd, *J* = 12.5 Hz, 7.2 Hz, 3H), 7.37 (dd, *J* = 9.0 Hz, 4.7 Hz, 1H), 7.33 (d, *J* = 1.4 Hz, 1H), 7.24–7.20 (m, 1H); ^13^C{^1^H} NMR (100 MHz, CDCl_3_): δ 165.1, 162.8, 159.7 (d, *J*_C–F_ = 242.9 Hz), 150.6, 146.9, 134.5 (d, *J*_C–F_ = 34.0 Hz), 129.3 (d, *J*_C–F_ = 4.5 Hz), 127.5, 119.8 (d, *J*_C–F_ = 8.4 Hz), 119.5 (d, *J*_C–F_ = 8.6 Hz), 118.3, 118.0, 115.8, 111.5, 109.6, 109.3, 104.8; ^19^F NMR (376 MHz, CDCl_3_): δ −116.09; HRMS (ESI-TOF) *m*/*z*: [M + H]^+^ calcd for C_19_H_12_FO_3_, 307.0765; found, 307.0768.

### 2-Chloro-7-hydroxy-9-phenyl-6*H*-benzo[*c*]chromen-6-one (Compound **3c**)

This compound was purified by column chromatography (ethyl acetate/petroleum ether = 1:10, *R*_*f*_ = 0.6) to afford a white solid in 82% yield (53 mg); mp = 219–220 °C; ^1^H NMR (400 MHz, CDCl_3_): δ 11.31 (s, 1H), 8.06 (d, *J* = 2.4 Hz, 1H), 7.71–7.68 (m, 3H), 7.54–7.45 (m, 4H), 7.35–7.32 (m, 2H); ^13^C{^1^H} NMR (100 MHz, CDCl_3_): δ 164.9, 162.8, 150.7, 149.2, 139.2, 134.3, 130.9, 130.8, 129.3, 129.3, 127.5, 123.2, 119.9, 119.3, 115.8, 111.4, 104.8; HRMS (ESI-TOF) *m*/*z*: [M + H]^+^ calcd for C_19_H_12_ClO_3_, 323.0469; found, 323.0467.

### 2-Bromo-7-hydroxy-9-phenyl-6*H*-benzo[*c*]chromen-6-one (Compound **3d**)

This compound was purified by column chromatography (ethyl acetate/petroleum ether = 1:10, *R*_*f*_ = 0.6) to afford a white solid in 85% yield (62 mg); mp = 210–211 °C; ^1^H NMR (400 MHz, CDCl_3_): δ 11.29 (s, 1H), 8.20 (d, *J* = 2.0 Hz, 1H), 7.70–7.68 (m, 3H), 7.59 (dd, *J* = 8.8 Hz, 2.2 Hz, 1H), 7.50 (dd, *J* = 12.9 Hz, 7.2 Hz, 3H), 7.33 (d, *J* = 1.2 Hz, 1H), 7.28 (s, 1H); ^13^C{^1^H} NMR (101 MHz, CDCl_3_): δ 164.9, 162.8, 150.7, 149.7, 139.3, 134.2, 133.6, 129.3, 129.3, 127.5, 126.3, 120.3, 119.7, 118.3, 115.9, 111.4, 104.8; HRMS (ESI-TOF) *m*/*z*: [M + H]^+^ calcd for C_19_H_12_BrO_3_, 366.9964; found, 366.9962.

### 7-Hydroxy-2-iodo-9-phenyl-6*H*-benzo[*c*]chromen-6-one (Compound **3e**)

This compound was purified by column chromatography (ethyl acetate/petroleum ether = 1:10, *R*_*f*_ = 0.6) to afford a white solid in 89% yield (74 mg); mp = 194–195 °C; ^1^H NMR (400 MHz, CDCl_3_): δ 11.28 (s, 1H), 8.38 (d, *J* = 1.6 Hz, 1H), 7.77 (dd, *J* = 8.6 Hz, 1.8 Hz, 1H), 7.70 (s, 1H), 7.68 (s, 2H), 7.54–7.46 (m, 3H), 7.32–7.31 (s, 1H), 7.13 (d, *J* = 8.6 Hz, 1H); ^13^C{^1^H} NMR (100 MHz, CDCl_3_): δ 164.8, 162.8, 150.7, 150.5, 139.4, 139.3, 134.0, 132.3, 129.3, 129.2, 127.5, 120.7, 119.9, 115.8, 111.3, 104.8, 88.7; HRMS (ESI-TOF) *m*/*z*: [M + H]^+^ calcd for C_19_H_12_IO_3_, 414.9826; found, 414.9818.

### 7-Hydroxy-2-methyl-9-(*p*-tolyl)-6*H*-benzo[*c*]chromen-6-one (Compound **3f**)^5e^

This compound was purified by column chromatography (ethyl acetate/petroleum ether = 1:10, *R*_*f*_ = 0.6) to afford a white solid in 62% yield (39 mg); mp = 265–266 °C; ^1^H NMR (400 MHz, CDCl_3_): δ 11.43 (s, 1H), 7.88 (s, 1H), 7.75 (s, 1H), 7.61 (d, *J* = 8.0 Hz, 2H), 7.32 (d, *J* = 7.6 Hz, 2H), 7.30 (s, 1H), 7.28 (s, 1H), 7.27 (s, 1H), 2.48 (s, 3H), 2.44 (s, 3H); ^13^C{^1^H} NMR (100 MHz, CDCl_3_): δ 165.7, 162.8, 150.3, 149.0, 139.3, 136.8, 135.6, 134.9, 131.7, 129.9, 127.4, 123.4, 118.1, 117.6, 114.7, 110.9, 104.8, 21.4, 21.3; HRMS (ESI-TOF) *m*/*z*: [M + H]^+^ calcd for C_21_H_17_O_3_, 317.1172; found, 317.1179.

### 2-Chloro-7-hydroxy-9-(*p*-tolyl)-6*H*-benzo[*c*]chromen-6-one (Compound **3g**)

This compound was purified by column chromatography (ethyl acetate/petroleum ether = 1:10, *R*_*f*_ = 0.6) to afford a white solid in 74% yield (50 mg); mp = 256–257 °C; ^1^H NMR (400 MHz, CDCl_3_): δ 11.29 (s, 1H), 8.06 (s, 1H), 7.70 (s, 1H), 7.60 (d, *J* = 7.8 Hz, 2H), 7.46 (d, *J* = 8.0 Hz, 1H), 7.33 (d, *J* = 7.6 Hz, 4H), 2.44 (s, 3H); ^13^C{^1^H} NMR (100 MHz, CDCl_3_): δ 165.0, 162.8, 150.6, 149.3, 139.5, 136.3, 134.3, 130.8, 130.7, 130.0, 127.4, 123.3, 119.9, 119.4, 115.5, 111.1, 104.6, 21.4; HRMS (ESI-TOF) *m*/*z*: [M + H]^+^ calcd for C_20_H_14_ClO_3_, 337.0626; found, 337.0621.

### 7-Hydroxy-4-methoxy-9-(*p*-tolyl)-6*H*-benzo[*c*]chromen-6-one (Compound **3h**)

This compound was purified by column chromatography (ethyl acetate/petroleum ether = 1:10, *R*_*f*_ = 0.6) to afford a white solid in 61% yield (41 mg); mp = 173–174 °C; ^1^H NMR (400 MHz, CDCl_3_): δ 11.39 (s, 1H), 7.75 (s, 1H), 7.68 (d, *J* = 8.0 Hz, 1H), 7.60 (d, *J* = 8.0 Hz, 2H), 7.33–7.28 (m, 4H), 7.06 (d, *J* = 8.0 Hz, 1H), 4.00 (s, 3H), 2.44 (s, 3H); ^13^C{^1^H} NMR (100 MHz, CDCl_3_): δ 164.9, 162.7, 150.3, 148.2, 140.7, 139.3, 136.7, 135.7, 129.9, 127.4, 124.9, 119.4, 114.8, 114.7, 112.4, 111.5, 104.8, 56.4, 21.4; HRMS (ESI-TOF) *m*/*z*: [M + H]^+^ calcd for C_21_H_17_O_4_, 333.1121; found, 333.1116.

### 2,4-Dichloro-7-hydroxy-9-phenyl-6*H*-benzo[*c*]chromen-6-one (Compound **3i**)

This compound was purified by column chromatography (ethyl acetate/petroleum ether = 1:10, *R*_*f*_ = 0.6) to afford a white solid in 93% yield (66 mg); mp = 185–186 °C; ^1^H NMR (400 MHz, CDCl_3_): δ 11.15 (s, 1H), 7.95 (d, *J* = 2.2 Hz, 1H), 7.69–7.65 (m, 3H), 7.55–7.46 (m, 4H), 7.34 (d, *J* = 1.2 Hz, 1H); ^13^C{^1^H} NMR (100 MHz, CDCl_3_): δ 163.8, 162.8, 150.9, 145.3, 139.0, 133.7, 130.8, 130.5, 129.5, 129.3, 127.5, 123.9, 121.7, 120.9, 116.4, 111.7, 104.6; HRMS (ESI-TOF) *m*/*z*: [M + H]^+^ calcd for C_19_H_11_Cl_2_O_3_, 357.0080; found, 357.0084.

### 2,4-Dibromo-7-hydroxy-9-phenyl-6*H*-benzo[*c*]chromen-6-one (Compound **3j**)

This compound was purified by column chromatography (ethyl acetate/petroleum ether = 1:10, *R*_*f*_ = 0.6) to afford a white solid in 91% yield (81 mg); mp = 204–205 °C; ^1^H NMR (400 MHz, CDCl_3_): δ 11.15 (s, 1H), 8.15 (d, *J* = 2.0 Hz, 1H), 7.85 (d, *J* = 2.0 Hz, 1H), 7.68 (d, *J* = 6.4 Hz, 3H), 7.55–7.47 (m, 3H), 7.35 (d, *J* = 1.2 Hz, 1H); ^13^C{^1^H} NMR (100 MHz, CDCl_3_): δ 163.9, 162.7, 150.9, 146.7, 138.9, 136.4, 133.5, 129.4, 129.3, 127.5, 125.4, 121.2, 116.3, 112.6, 111.6, 104.5; HRMS (ESI-TOF) *m*/*z*: [M + H]^+^ calcd for C_19_H_11_Br_2_O_3_, 444.9069; found, 444.9066.

### 3-Chloro-7-hydroxy-9-phenyl-6*H*-benzo[*c*]chromen-6-one (Compound **3k**)

This compound was purified by column chromatography (ethyl acetate/petroleum ether = 1:10, *R*_*f*_ = 0.6) to afford a white solid in 75% yield (48 mg); mp = 180–181 °C. ^1^H NMR (400 MHz, CDCl_3_): δ 11.23 (s, 1H), 8.02 (d, *J* = 8.6 Hz, 1H), 7.70 (d, *J* = 1.4 Hz, 1H), 7.69–7.66 (m, 2H), 7.54–7.47 (m, 3H), 7.37 (d, *J* = 2.0 Hz, 1H), 7.34 (dd, *J* = 8.5 Hz, 2.0 Hz, 1H), 7.29 (d, *J* = 1.4 Hz, 1H); ^13^C{^1^H} NMR (100 MHz, CDCl_3_): δ 164.9, 162.8, 151.0, 150.7, 139.3, 136.3, 134.7, 129.3, 129.2, 127.5, 125.7, 124.5, 118.1, 117.2, 115.4, 111.2, 104.6; HRMS (ESI-TOF) *m*/*z*: [M + H]^+^ calcd for C_19_H_12_ClO_3_, 323.0469; found, 323.0468.

### 2-Chloro-7-hydroxy-9-(4-methoxyphenyl)-6*H*-benzo[*c*]chromen-6-one (Compound **3L**)

This compound was purified by column chromatography (ethyl acetate/petroleum ether = 1:10, *R*_*f*_ = 0.6) to afford a white solid in 70% yield (49 mg); mp = 215–216 °C; ^1^H NMR (400 MHz, CDCl_3_): δ 11.28 (s, 1H), 8.05 (d, *J* = 2.4 Hz, 1H), 7.65 (d, *J* = 8.4 Hz, 3H), 7.45 (dd, *J* = 8.8, 2.4 Hz, 1H), 7.32 (d, *J* = 8.8 Hz, 1H), 7.29 (d, *J* = 1.4 Hz, 1H), 7.04 (d, *J* = 8.8 Hz, 2H), 3.89 (s, 3H); ^13^C{^1^H} NMR (100 MHz, CDCl_3_): δ 165.0, 162.8, 160.8, 150.2, 149.3, 134.2, 131.5, 130.8, 130.7, 128.7, 123.2, 119.99, 119.3, 115.1, 114.7, 110.8, 104.3, 55.6; HRMS (ESI-TOF) *m*/*z*: [M + H]^+^ calcd for C_20_H_14_ClO_4_, 353.0575; found, 353.0578.

### 9-(4-Fluorophenyl)-7-hydroxy-6*H*-benzo[*c*]chromen-6-one (Compound **3m**)

This compound was purified by column chromatography (ethyl acetate/petroleum ether = 1:10, *R*_*f*_ = 0.6) to afford a white solid in 80% yield (49 mg); mp = 246–247 °C; ^1^H NMR (400 MHz, CDCl_3_): δ 11.41 (s, 1H), 8.11 (d, *J* = 8.0 Hz, 1H), 7.73 (s, 1H), 7.69–7.65 (m, 2H), 7.55–7.50 (m, 1H), 7.40 (d, *J* = 8.8 Hz, 2H), 7.25 (d, *J* = 1.2 Hz, 1H), 7.21 (t, *J* = 8.8, 8.4 Hz, 2H); ^13^C{^1^H} NMR (100 MHz, CDCl_3_): δ 165.4, 163.5 (d, *J*_C–F_ = 247.7 Hz), 162.8, 150.9, 149.3, 135.7 (d, *J*_C–F_ = 8.3 Hz), 130.9, 129.3 (d, *J*_C–F_ = 8.3 Hz), 125.5, 123.4, 118.4, 118.0, 116.2 (d, *J*_C–F_ = 21.6 Hz), 114.9, 111.0, 105.0; ^19^F NMR (376 MHz, CDCl_3_): δ −112.55; HRMS (ESI-TOF) *m*/*z*: [M + H]^+^ calcd for C_19_H_12_FO_3_, 307.0765; found, 307.0775.

### 9-(4-Chlorophenyl)-7-hydroxy-6*H*-benzo[*c*]chromen-6-one (Compound **3n**)

This compound was purified by column chromatography (ethyl acetate/petroleum ether = 1:10, *R*_*f*_ = 0.6) to afford a white solid in 76% yield (49 mg); mp = 241–242 °C; ^1^H NMR (400 MHz, CDCl_3_): δ 11.41 (s, 1H), 8.12 (dd, *J* = 8.0 Hz, 1.2 Hz, 1H), 7.74 (d, *J* = 1.2 Hz, 1H), 7.69–7.66 (m, 2H), 7.55–7.51 (m, 1H), 7.41 (d, *J* = 2.4 Hz, 1H), 7.40–7.38 (m, 1H), 7.25 (d, *J* = 1.2 Hz, 1H), 7.20 (d, *J* = 8.6 Hz, 2H); ^13^C{^1^H} NMR (125 MHz, CDCl_3_): δ 165.4, 162.8, 150.9, 149.1, 138.1, 135.8, 135.4, 131.0, 129.4, 128.8, 125.3, 123.5, 118.4, 118.0, 114.9, 111.0, 105.3; HRMS (ESI-TOF) *m*/*z*: [M + H]^+^ calcd for C_19_H_12_ClO_3_, 323.0469; found, 323.0466.

### 9-(4-Bromophenyl)-7-hydroxy-6*H*-benzo[*c*]chromen-6-one (Compound **3o**)^5f^

This compound was purified by column chromatography (ethyl acetate/petroleum ether = 1:10, *R*_*f*_ = 0.6) to afford a white solid in 70% yield (51 mg); mp = 220–221 °C; ^1^H NMR (400 MHz, CDCl_3_): δ 11.41 (s, 1H), 8.10 (d, *J* = 8.0 Hz, 1H), 7.73 (d, *J* = 1.6 Hz, 1H), 7.66 (s, 1H), 7.64 (s, 1H), 7.57–7.53 (m, 3H), 7.40 (d, *J* = 8.0 Hz, 2H), 7.24 (d, *J* = 1.6 Hz, 1H); ^13^C{^1^H} NMR (100 MHz, CDCl_3_): δ 165.4, 162.8, 150.9, 149.1, 138.5, 135.8, 132.4, 131.0, 129.1, 125.3, 123.6, 123.4, 118.3, 118.0, 114.9, 110.9, 105.3; HRMS (ESI-TOF) *m*/*z*: [M + H]^+^ calcd for C_19_H_12_BrO_3_, 366.9970; found, 366.9962.

### 7-Hydroxy-9-(4-(trifluoromethyl)phenyl)-6*H*-benzo[*c*]chromen-6-one (Compound **3p**)

This compound was purified by column chromatography (ethyl acetate/petroleum ether = 1:10, *R*_*f*_ = 0.6) to afford a white solid in 84% yield (60 mg); mp = 199–200 °C; ^1^H NMR (400 MHz, CDCl_3_): δ 11.44 (s, 1H), 8.10 (d, *J* = 8.4 Hz, 1H), 7.77 (dd, *J* = 8.0 Hz, 1.6 Hz, 5H), 7.56–7.52 (m, 1H), 7.40 (d, *J* = 8.0 Hz, 2H), 7.28 (d, *J* = 1.6 Hz, 1H); ^13^C{^1^H} NMR (100 MHz, CDCl_3_): δ 165.3, 162.9, 150.9, 148.8, 143.1, 135.9, 131.1, 127.9, 126.1 (q, *J*_C–F_ = 3.8 Hz), 125.4, 123.4, 118.2, 118.0, 115.3, 111.3, 105.7; ^19^F NMR (376 MHz, CDCl_3_): δ −62.60; HRMS (ESI-TOF) *m*/*z*: [M + H]^+^ calcd for C_20_H_12_F_3_O_3_, 357.0733; found, 357.0739.

### 2-Bromo-9-(4-bromophenyl)-7-hydroxy-6*H*-benzo[*c*]chromen-6-one (Compound **3q**)

This compound was purified by column chromatography (ethyl acetate/petroleum ether = 1:10, *R*_*f*_ = 0.6) to afford a white solid in 87% yield (77 mg); mp = 235–236 °C; ^1^H NMR (400 MHz, CDCl_3_): δ 11.32 (s, 1H), 8.21 (d, *J* = 2.1 Hz, 1H), 7.68–7.65 (m, 3H), 7.61 (dd, *J* = 8.8 Hz, 2.1 Hz, 1H), 7.57 (s, 1H), 7.55 (s, 1H), 7.29 (t, *J* = 4.4, 3.2 Hz, 2H); ^13^C{^1^H} NMR (100 MHz, CDCl_3_): δ 164.8, 162.9, 149.8, 149.4, 138.2, 134.4, 133.8, 132.5, 129.1, 126.3, 123.9, 120.2, 119.7, 118.4, 115.6, 111.1, 105.1; HRMS (ESI-TOF) *m*/*z*: [M – H]^−^ calcd for C_19_H_11_Br_2_O_3_, 442.8924; found, 442.8933.

### 2-Bromo-7-hydroxy-9-(*p*-tolyl)-6*H*-benzo[*c*]chromen-6-one (Compound **3r**)

This compound was purified by column chromatography (ethyl acetate/petroleum ether = 1:10, *R*_*f*_ = 0.6) to afford a white solid in 71% yield (54 mg); mp = 262–263 °C; ^1^H NMR (400 MHz, CDCl_3_): δ 11.28 (s, 1H), 8.21 (s, 1H), 7.70 (s, 1H), 7.60 (d, *J* = 7.2 Hz, 3H), 7.33 (d, *J* = 7.0 Hz, 3H), 7.29 (s, 1H), 2.44 (s, 3H); ^13^C{^1^H} NMR (100 MHz, CDCl_3_): δ 164.9, 162.8, 150.7, 149.8, 139.6, 136.3, 134.1, 133.6, 130.0, 127.4, 126.3, 120.4, 119.7, 118.3, 115.6, 111.1, 104.6, 21.4; HRMS (ESI-TOF) *m*/*z*: [M + H]^+^ calcd for C_20_H_14_BrO_3_, 381.0121; found, 381.0130.

### 2-Bromo-9-(4-ethylphenyl)-7-hydroxy-6*H*-benzo[*c*]chromen-6-one (Compound **3s**)

This compound was purified by column chromatography (ethyl acetate/petroleum ether = 1:10, *R*_*f*_ = 0.6) to afford a white solid in 73% yield (58 mg); mp = 211–212 °C; ^1^H NMR (400 MHz, CDCl_3_): δ 11.26 (s, 1H), 8.17 (d, *J* = 2.2 Hz, 1H), 7.66 (d, *J* = 1.2 Hz, 1H), 7.61 (d, *J* = 8.0 Hz, 2H), 7.57 (dd, *J* = 8.8 Hz, 2.2 Hz, 1H), 7.35 (d, *J* = 8.0 Hz, 2H), 7.30 (d, *J* = 1.2 Hz, 1H), 7.24 (d, *J* = 8.8 Hz, 1H), 2.73 (t, *J* = 7.6 Hz, 2H), 1.30 (t, *J* = 7.6 Hz, 3H); ^13^C{^1^H} NMR (100 MHz, CDCl_3_): δ 164.8, 162.7, 150.6, 149.7, 145.8, 136.5, 134.0, 133.5, 128.8, 127.4, 126.2, 120.3, 119.6, 118.2, 115.5, 111.1, 104.5, 28.7, 15.6; HRMS (ESI-TOF) *m*/*z*: [M + H]^+^ calcd for C_21_H_16_BrO_3_ 395.0277; found, 395.0271.

### 2-Bromo-9-(4-chlorophenyl)-7-hydroxy-6*H*-benzo[*c*]chromen-6-one (Compound **3t**)

This compound was purified by column chromatography (ethyl acetate/petroleum ether = 1:10, *R*_*f*_ = 0.6) to afford a white solid in 70% yield (56 mg); mp = 247–248 °C; ^1^H NMR (500 MHz, CDCl_3_): δ 11.30 (s, 1H), 8.19 (d, *J* = 2.2 Hz, 1H), 7.64 (d, *J* = 1.0 Hz, 1H), 7.62 (s, 1H), 7.61–7.58 (m, 2H), 7.49 (d, *J* = 8.5 Hz, 2H), 7.28–7.27 (m, 1H), 7.24 (s, 1H); ^13^C{^1^H} NMR (100 MHz, CDCl_3_): δ 164.8, 162.9, 149.8, 149.4, 137.7, 135.6, 134.4, 133.8, 129.5, 128.8, 126.3, 120.2, 119.7, 118.4, 115.7, 111.1, 105.1; HRMS (ESI-TOF) *m*/*z*: [M + H]^+^ calcd for C_19_H_11_BrClO_3_, 400.9575; found, 400.9585.

### 9-(4-Bromophenyl)-2-chloro-7-hydroxy-6*H*-benzo[*c*]chromen-6-one (Compound **3u**)

This compound was purified by column chromatography (ethyl acetate/petroleum ether = 1:10, *R*_*f*_ = 0.6) to afford a white solid in 80% yield (64 mg); mp = 234–235 °C; ^1^H NMR (500 MHz, CDCl_3_): δ 11.32 (s, 1H), 8.05 (d, *J* = 2.4 Hz, 1H), 7.67–7.65 (m, 3H), 7.57–7.55 (m, 2H), 7.47 (dd, *J* = 8.8 Hz, 2.4 Hz, 1H), 7.34 (d, *J* = 8.8 Hz, 1H), 7.29 (d, *J* = 1.5 Hz, 1H); ^13^C{^1^H} NMR (100 MHz, CDCl_3_): δ 164.9, 162.9, 149.4, 149.3, 138.2, 134.5, 132.4, 130.9, 129.1, 123.9, 123.2, 119.7, 119.4, 115.6, 111.1, 105.1; HRMS (ESI-TOF) *m*/*z*: [M + H]^+^ calcd for C_19_H_11_BrClO_3_, 400.9575; found, 400.9569.

### 9-(3-Bromophenyl)-7-hydroxy-6*H*-benzo[*c*]chromen-6-one (Compound **3v**)

This compound was purified by column chromatography (ethyl acetate/petroleum ether = 1:10, *R*_*f*_ = 0.6) to afford a white solid in 90% yield (66 mg); mp = 192–193 °C; ^1^H NMR (400 MHz, CDCl_3_): δ 11.41 (s, 1H), 8.13 (d, *J* = 8.2 Hz, 1H), 7.82 (t, *J* = 1.6 Hz, 1H), 7.72 (d, *J* = 1.4 Hz, 1H), 7.62–7.58 (m, 2H), 7.55–7.51 (m, 1H), 7.41–7.37 (m, 3H), 7.24 (d, *J* = 1.4 Hz, 1H); ^13^C{^1^H} NMR (100 MHz, CDCl_3_): δ 165.3, 162.8, 150.9, 148.8, 141.7, 135.8, 132.0, 131.0, 130.7, 130.5, 126.2, 125.4, 123.5, 123.3, 118.3, 118.0, 115.1, 111.1, 105.4; HRMS (ESI-TOF) *m*/*z*: [M + H]^+^ calcd for C_19_H_12_BrO_3_, 366.9964; found, 366.9965.

### 9-(3-Fluorophenyl)-7-hydroxy-6*H*-benzo[*c*]chromen-6-one (Compound **3w**)

This compound was purified by column chromatography (ethyl acetate/petroleum ether = 1:10, *R*_*f*_ = 0.6) to afford a white solid in 81% yield (50 mg); mp = 188–189 °C; ^1^H NMR (400 MHz, CDCl_3_): δ 11.42 (d, *J* = 1.6 Hz, 1H), 8.13 (d, *J* = 8.0 Hz, 1H), 7.76 (s, 1H), 7.56–7.47 (m, 3H), 7.42–7.37 (m, 3H), 7.27 (d, *J* = 4.4 Hz, 1H), 7.20–7.14 (m, 1H); ^13^C{^1^H} NMR (125 MHz, CDCl_3_): δ 165.4, 162.8, 150.9, 149.0, 141.9 (d, *J*_C–F_ = 7.4 Hz), 135.8, 131.0, 130.8 (d, *J*_C–F_ = 8.3 Hz), 125.4, 123.5, 123.2 (d, *J*_C–F_ = 2.8 Hz), 118.3, 118.0, 116.1, 115.9, 114.5 (d, *J*_C–F_ = 178.0 Hz), 111.2, 105.5; ^19^F NMR (376 MHz, CDCl_3_): δ −112.08 ppm. HRMS (ESI-TOF) *m*/*z*: [M + H]^+^ calcd for C_19_H_12_FO_3_, 307.0765; found, 307.0768.

### 9-(3-Chlorophenyl)-7-hydroxy-6*H*-benzo[*c*]chromen-6-one (Compound **3x**)

This compound was purified by column chromatography (ethyl acetate/petroleum ether = 1:10, *R*_*f*_ = 0.6) to afford a white solid in 68% yield (44 mg); mp = 191–192 °C; ^1^H NMR (400 MHz, CDCl_3_): δ 11.40 (s, 1H), 8.11 (d, *J* = 7.8 Hz, 1H), 7.72 (d, *J* = 1.2 Hz, 1H), 7.66 (s, 1H), 7.57–7.54 (m, 1H), 7.53–7.50 (m, 1H), 7.46–7.43 (m, 2H), 7.41–7.37 (m, 2H), 7.24 (d, *J* = 1.4 Hz, 1H); ^13^C{^1^H} NMR (100 MHz, CDCl_3_): δ 165.3, 162.8, 150.9, 148.8, 141.4, 135.8, 135.1, 131.0, 130.4, 129.1, 127.6, 125.7, 125.4, 123.5, 118.3, 118.0, 115.1, 111.1, 105.4; HRMS (ESI-TOF) *m*/*z*: [M + H]^+^ calcd for C_19_H_12_ClO_3_, 323.0469; found, 323.0460.

### 7-Hydroxy-9-(*m*-tolyl)-6*H*-benzo[*c*]chromen-6-one (Compound **3y**)

This compound was purified by column chromatography (ethyl acetate/petroleum ether = 1:10, *R*_*f*_ = 0.6) to afford a white solid in 71% yield (43 mg); mp = 166–167 °C; ^1^H NMR (400 MHz, CDCl_3_): δ 11.37 (s, 1H), 8.12 (d, *J* = 7.4 Hz, 1H), 7.77 (d, *J* = 1.4 Hz, 1H), 7.52–7.47 (m, 3H), 7.40–7.35 (m, 3H), 7.28 (d, *J* = 1.4 Hz, 1H), 7.24 (s, 1H), 2.46 (s, 3H); ^13^C{^1^H} NMR (100 MHz, CDCl_3_): δ 165.5, 162.7, 150.9, 150.6, 139.6, 138.9, 135.5, 130.8, 129.9, 129.1, 128.2, 125.3, 124.7, 123.5, 118.6, 117.9, 115.1, 111.3, 105.0, 21.7; HRMS (ESI-TOF) *m*/*z*: [M + H]^+^ calcd for C_20_H_15_O_3_, 303.1016; found, 303.1020.

### 9-(2-Fluorophenyl)-7-hydroxy-6*H*-benzo[*c*]chromen-6-one (Compound **3z**)

This compound was purified by column chromatography (ethyl acetate/petroleum ether = 1:10, *R*_*f*_ = 0.6) to afford a white solid in 80% yield (49 mg); mp = 202–203 °C. ^1^H NMR (500 MHz, CDCl_3_): δ 11.39 (s, 1H), 8.08 (dd, *J* = 8.0 Hz, 1.5 Hz, 1H), 7.78 (s, 1H), 7.54–7.50 (m, 2H), 7.48–7.33 (m, 4H), 7.30–7.28 (m, 1H), 7.25–7.21 (s, 1H); ^13^C{^1^H} NMR (100 MHz, CDCl_3_): δ 165.4, 162.4, 159.8 (d, *J*_C–F_ = 248.3 Hz), 150.8, 145.1, 135.2, 130.8 (d, *J*_C–F_ = 10.0 Hz), 127.6 (d, *J*_C–F_ = 12.7 Hz), 125.3, 124.8 (d, *J*_C–F_ = 3.7 Hz), 123.5, 118.4, 117.9, 117.0 (d, *J*_C–F_ = 2.5 Hz), 116.7, 116.5, 113.3 (d, *J*_C–F_ = 3.8 Hz), 105.3; ^19^F NMR (376 MHz, CDCl_3_): δ −62.76; HRMS (ESI-TOF) *m*/*z*: [M + H]^+^ calcd for C_19_H_12_FO_3_, 307.0765; found, 307.0768.

### 7-Hydroxy-9-(naphthalen-1-yl)-6*H*-benzo[*c*]chromen-6-one (Compound **3A**)^24^

This compound was purified by column chromatography (ethyl acetate/petroleum ether = 1:12, *R*_*f*_ = 0.6) to afford a white solid in 73% yield (49 mg); mp = 223–224 °C; ^1^H NMR (400 MHz, CDCl_3_): δ 11.49 (s, 1H), 8.03 (d, *J* = 8.0 Hz, 1H), 7.97 (d, *J* = 8.2 Hz, 2H), 7.93 (d, *J* = 8.4 Hz, 1H), 7.74 (s, 1H), 7.59 (dd, *J* = 14.3 Hz, 6.4 Hz, 2H), 7.54–7.48 (m, 3H), 7.44 (d, *J* = 8.2 Hz, 1H), 7.36 (d, *J* = 7.6 Hz, 1H), 7.27 (d, *J* = 5.6 Hz, 1H); ^13^C{^1^H} NMR (100 MHz, CDCl_3_): δ 165.5, 162.4, 150.9, 150.5, 138.6, 135.2, 133.9, 131.0, 130.9, 129.0, 128.6, 126.9, 126.8, 126.3, 125.5, 125.4, 125.3, 123.6, 118.4, 118.2, 117.9, 114.4, 105.2; HRMS (ESI-TOF) *m*/*z*: [M + H]^+^ calcd for C_23_H_15_O_3_, 339.1016; found, 339.1024.

### 7-Hydroxy-9-(thiophen-3-yl)-6*H*-benzo[*c*]chromen-6-one (Compound **3B**)

This compound was purified by column chromatography (ethyl acetate/petroleum ether = 1:10, *R*_*f*_ = 0.6) to afford a white solid in 76% yield (45 mg); mp = 216–217 °C; ^1^H NMR (400 MHz, CDCl_3_): δ 11.39 (s, 1H), 8.11 (d, *J* = 8.0 Hz, 1H), 7.79 (d, *J* = 1.2 Hz, 1H), 7.71 (t, *J* = 2.8, 1.6 Hz, 1H), 7.52–7.47 (m, 3H), 7.41–7.37 (m, 2H), 7.30 (d, *J* = 1.2 Hz, 1H); ^13^C{^1^H} NMR (100 MHz, CDCl_3_): δ 165.3, 162.9, 150.9, 144.5, 140.8, 135.7, 130.9, 127.2, 126.3, 125.3, 123.6, 123.4, 118.4, 118.0, 114.1, 110.3, 104.9; HRMS (ESI-TOF) *m*/*z*: [M + H]^+^ calcd for C_17_H_11_O_3_S, 295.0423; found, 295.0428.

### 7-Hydroxy-9-(thiophen-2-yl)-6*H*-benzo[*c*]chromen-6-one (Compound **3C**)

This compound was purified by column chromatography (ethyl acetate/petroleum ether = 1:10, *R*_*f*_ = 0.6) to afford a white solid in 64% yield (38 mg); mp = 220–221 °C; ^1^H NMR (400 MHz, CDCl_3_): δ 11.39 (s, 1H), 8.10 (d, *J* = 7.6 Hz, 1H), 7.78 (s, 1H), 7.70 (s, 1H), 7.54–7.47 (m, 3H), 7.41–7.36 (m, 2H), 7.29 (s, 1H); ^13^C{^1^H} NMR (100 MHz, CDCl_3_): δ 165.3, 162.9, 150.9, 144.5, 140.8, 135.7, 130.9, 127.2, 126.3, 125.3, 123.6, 123.4, 118.4, 118.0, 114.1, 110.3, 104.9; HRMS (ESI-TOF) *m*/*z*: [M + H]^+^ calcd for C_17_H_11_O_3_S, 295.0423; found, 295.0413.

### 7-Methoxy-9-phenyl-6*H*-benzo[*c*]chromen-6-one (Compound **7**)

According to the reported literature,^25^ in a resealable screw-capped Schlenk tube equipped with magnetic stirring bar, 7-hydroxy-9-phenyl-6*H*-benzo[*c*]chromen-6-one (**3a**) (0.2 mmol, 1 equiv) was dissolved in DMF (5 mL). Then, K_2_CO_3_ (2 equiv) and MeI (2 equiv) were added in tube. The mixture was stirred at 25 °C until the substrate was consumed, as judged by TLC (∼6 h). Upon completion of the reaction, the mixture was cooled to room temperature, extracted with CH_2_Cl_2_ (3 × 20 mL), and washed with brine. The organic layers were combined, dried over Na_2_SO_4_, filtered, and then evaporated under vacuum. The residue was purified using flash column chromatography (ethyl acetate/petroleum ether = 1:8) to give the desired product **7** as a white solid in 82% yield (50 mg). mp = 154–155 °C; ^1^H NMR (400 MHz, CDCl_3_): δ 8.07 (d, *J* = 8.0 Hz, 1H), 7.85 (d, *J* = 1.0 Hz, 1H), 7.69 (d, *J* = 7.8 Hz, 2H), 7.57–7.43 (m, 4H), 7.34–7.27 (m, 2H), 7.21 (s, 1H), 4.10 (s, 3H); ^13^C{^1^H} NMR (100 MHz, CDCl_3_): δ 162.6, 157.9, 151.3, 148.8, 139.9, 137.8, 130.8, 129.2, 129.0, 127.5, 124.2, 123.3, 117.9, 117.5, 112.4, 110.1, 108.7, 56.6; HRMS (ESI-TOF) *m*/*z*: [M + H]^+^ calcd for C_20_H_15_O_3_, 303.1016; found, 303.1018.

### 7-(Benzyloxy)-9-phenyl-6*H*-benzo[*c*]chromen-6-one (Compound **8**)

According to the reported literature,^26^ to compound **3a** (0.2 mmol, 1 equiv) in CH_3_CN (2 mL), (bromomethyl)benzene (1.5 equiv) and K_2_CO_3_ (2.0 equiv) were added in a reaction flask under air atmosphere. Then, the solution was mixed at 55 °C. The reaction mixture was stirred overnight. After the completion of the reaction (monitored by TLC), the mixture was cooled to room temperature. The crude products were extracted with CH_2_Cl_2_ (3 × 20 mL) and washed with brine. The organic layers were combined, dried over Na_2_SO_4_, filtered, and then evaporated under vacuum. The residue was purified using flash column chromatography (ethyl acetate/petroleum ether = 1:8) to give the desired product **8** as a white solid in 84% yield (64 mg). mp = 174–175 °C; ^1^H NMR (400 MHz, CDCl_3_): δ 8.06 (d, *J* = 7.8 Hz, 1H), 7.85 (s, 1H), 7.65–7.60 (m, 4H), 7.52–7.39 (m, 6H), 7.34–7.27 (m, 3H), 7.24 (s, 1H), 5.38 (s, 2H); ^13^C{^1^H} NMR (100 MHz, CDCl_3_): δ 161.4, 157.7, 151.9, 148.5, 139.8, 137.8, 136.4, 130.7, 129.2, 129.0, 128.7, 127.9, 127.5, 126.8, 124.1, 123.3, 117.9, 117.5, 112.7, 112.1, 109.4, 71.0; HRMS (ESI-TOF) *m*/*z*: [M + H]^+^ calcd for C_26_H_19_O_3_, 379.1329; found, 379.1326.

### 6-Oxo-9-phenyl-6*H*-benzo[*c*]chromen-7-yl Trifluoromethanesulfonate (Compound **9**)

According to the reported literature,^27^ compound **3a** (0.5 mmol, 1.0 equiv), pyridine (2.0 equiv), and dichloromethane (3.0 mL) were mixed in a reaction flask under a nitrogen atmosphere. Then, the solution was cooled to 0 °C, and a solution of trifluoromethanesulfonic anhydride (1.5 equiv) in dichloromethane (2.0 mL) was added in a dropwise manner within 30 min. The reaction mixture was stirred overnight. After the completion of the reaction, 25 mL of water was added to the mixture and stirred for 3 h. The mixture was extracted with ethyl acetate (∼20 mL), and the organic layer was washed with water (∼20 mL), 10% aqueous HCl (∼10 mL) three times, water (∼20 mL, twice), saturated aqueous NaHCO_3_ (twice), and brine (10 mL, twice), and dried over Na_2_SO_4_. The organic layer was evaporated under vacuum, and the crude product was purified by flash column chromatography (ethyl acetate/petroleum ether = 1:8) to afford the desired product **9** as a white solid in 62% yield (1.30 g). mp = 149–150 °C; ^1^H NMR (400 MHz, CDCl_3_): δ 8.32 (s, 1H), 8.12 (d, *J* = 8.0 Hz, 1H), 7.68 (d, *J* = 7.2 Hz, 2H), 7.61–7.50 (m, 5H), 7.41–7.33 (m, 2H); ^13^C{^1^H} NMR (100 MHz, CDCl_3_): δ 156.8, 151.6, 150.4, 149.1, 138.2, 137.7, 131.9, 129.9, 129.6, 127.5, 125.0, 123.4, 121.6, 120.3, 117.9, 116.8, 113.0; ^19^F NMR (376 MHz, CDCl_3_): δ −73.03; HRMS (ESI-TOF) *m*/*z*: [M + H]^+^ calcd for C_20_H_12_F_3_O_5_S, 421.0352; found, 421.0366.

### 7-(Diphenylphosphoryl)-9-phenyl-6*H*-benzo[*c*]chromen-6-one (Compound **10**)

According to the reported literature,^25^ a solution of Pd(OAc)_2_ (20 mmol %) and 1,3-bis(diphenylphosphino)propane (dppp) (20 mmol %) in dry DMSO (5 mL) was stirred at room temperature for 0.5 h under N_2_. Subsequently, **9** (0.2 mmol, 1 equiv), diphenylphosphine oxide (2.5 equiv) and DIPEA (5 equiv) were added. The reaction was stirred at 120 °C for 4 h. The reaction was quenched with H_2_O. The solution was extracted with CH_2_Cl_2_ (3 × 20 mL), washed with H_2_O and brine, and dried over anhydrous Na_2_SO_4_. The solvent was evaporated under reduced pressure. The crude product was purified by flash column chromatography (ethyl acetate/petroleum ether = 1:6) to give product **10** as a white solid in 75% yield (71 mg). mp = 290–291 °C; ^1^H NMR (400 MHz, CDCl_3_): δ 8.86 (d, *J* = 14.0 Hz, 1H), 8.58 (s, 1H), 8.20 (d, *J* = 7.8 Hz, 1H), 7.78 (m, 6H), 7.54–7.47 (m, 6H), 7.44 (dd, *J* = 7.6 Hz, 2.8 Hz, 4H), 7.37 (t, *J* = 7.6 Hz, 1H), 7.28 (s, 1H); ^13^C{^1^H} NMR (100 MHz, CDCl_3_): δ 158.8, 151.4, 147.0 (d, *J*_*C–P*_ = 11.7 Hz), 138.5, 137.4, 136.8(d, *J*_*C–P*_ = 7.8 Hz), 136.5, 133.8, 132.6, 131.7, 131.6, 131.3 (d, *J*_*C–P*_ = 11.6 Hz), 129.4 (d, *J*_*C–P*_ = 8.3 Hz), 128.3 (d, *J*_*C–P*_ = 12.8 Hz), 127.7, 124.8, 123.7, 123.2, 121.2 (d, *J*_*C–P*_ = 3.8 Hz), 117.6, 117.5; ^31^P NMR (162 MHz, CDCl_3_): δ 32.57; HRMS (ESI-TOF) *m*/*z*: [M + H]^+^ calcd for C_31_H_22_O_3_P, 473.1301; found, 473.1300.

### 7-(4-(Diphenylamino)phenyl)-9-phenyl-6*H*-benzo[*c*]chromen-6-one (Compound **11**)

According to the reported literature,^27^ to a 25 mL sealed tube containing a magnetic stir bar, were added compound **9** (0.2 mmol, 1.0 equiv), 4-(diphenylamino) phenylboronic acid (1.2 equiv), Pd(PPh_3_)_4_ (5 mol %), XPhos (10 mol %), K_3_PO_4_ (2.0 equiv), and 1,4-dioxane (4 mL). The tube was sealed under nitrogen and heated to 100 °C with stirring for 12 h. Then, the reaction mixture was cooled to room temperature and washed with ethyl acetate (2 × 10 mL). The combined organic layer was concentrated under vacuum, and the residue was purified by flash column chromatography (ethyl acetate/petroleum ether = 1:10) to afford the desired product **11** as a yellow solid in 82% yield (85 mg). mp = 262–263 °C; ^1^H NMR (400 MHz, CDCl_3_): δ 8.33 (d, *J* = 1.6 Hz, 1H), 8.19 (d, *J* = 8.0 Hz, 1H), 7.75 (d, *J* = 7.1 Hz, 2H), 7.70 (d, *J* = 1.7 Hz, 1H), 7.56–7.45 (m, 4H), 7.36 (d, *J* = 7.7 Hz, 2H), 7.32–7.26 (m, 6H), 7.21 (d, *J* = 7.4 Hz, 4H), 7.14 (d, *J* = 8.6 Hz, 2H), 7.04 (t, *J* = 7.2 Hz, 2H); ^13^C{^1^H} NMR (100 MHz, CDCl_3_): δ 159.5, 151.8, 147.8, 147.4, 147.2, 146.4, 139.4, 136.8, 135.8, 131.5, 130.6, 129.4, 129.4, 129.2, 129.0, 127.6, 124.7, 124.3, 123.2, 123.0, 122.7, 119.3, 118.3, 117.6,117.4; HRMS (ESI-TOF) *m*/*z*: [M + H]^+^ calcd for C_37_H26NO_2_, 516.1958; found, 516.1972.

### 6,6-Dimethyl-9-phenyl-6*H*-benzo[*c*]chromen-7-yl Trifluoromethanesulfonate (Compound **12**)

According to the reported literature,^27^ an oven-dried two-neck flask equipped with a magnetic stir bar was charged with compound **9** (0.2 mmol, 1 equiv) and NiCl_2_dppp (5 mol %), then purged with argon three times. Anhydrous THF (1 mL) followed by MeMgBr (0.3 mL, 1 mol/L in THF, 0.6 mmol) were added. The reaction was heated to reflux for 2 h. Water was added, and the mixture was extracted with EtOAc. The organic phase was washed with brine, dried over anhydrous Na_2_SO_4_, filtered, and the filtrated was concentrated under vacuum to obtain the residue, which was purified by flash column chromatography (ethyl acetate/petroleum ether = 1:20) to give the desired product **12** as a white solid in 55% yield (48 mg). mp = 129–130 °C; ^1^H NMR (400 MHz, CDCl_3_): δ 7.59 (d, *J* = 2.0 Hz, 1H), 7.56–7.53 (m, 2H), 7.47–7.39 (m, 3H), 7.36 (d, *J* = 2.0 Hz, 1H), 7.32–7.27 (m, 1H), 7.10 (dd, *J* = 7.5, 1.6 Hz, 1H), 7.01–6.94 (m, 2H), 1.66 (s, 3H), 1.54 (s, 3H); ^13^C{^1^H} NMR (100 MHz, CDCl_3_): δ 152.4, 149.1, 141.5, 139.0, 137.9, 137.9, 131.2, 130.1, 129.9, 129.7, 129.2, 128.7, 127.0, 120.8, 120.7, 116.2, 74.0, 31.6, 31.4; ^19^F NMR (376 MHz, CDCl_3_): δ −74.0; HRMS (ESI-TOF) *m*/*z*: [M + H]^+^ calcd for C_22_H_18_F_3_O_4_S, 435.0872; found, 435.0885.

### 7-Hydroxy-9-phenyl-2-(phenylethynyl)-6*H*-benzo[*c*]chromen-6-one (Compound **13**)

According to the reported literature,^28^ in a Schlenk tube equipped with magnetic stirring bar and reflux condenser, Pd(PPh_3_)_4_Cl_2_ (10 mmol %), CuI (10 mmol %), ethynylbenzene (2.0 equiv), and 7-hydroxy-2-iodo-9-phenyl-6*H*-benzo[*c*]chromen-6-one (**3e**) (0.2 mmol, 1.0 equiv) were added under an argon atmosphere followed by dry THF (1.5 mL) and Et_3_N (1.5 mL). The mixture was stirred at 50 °C for 12 h (TLC). It was stirred at this temperature until all of the raw material was consumed. Reaction was quenched with brine (20 mL) and extracted with dichloromethane (3 × 20 mL). The organic phase was separated and dried with anhydrous magnesium sulfate. The solvent was removed by rotary evaporation, and the residue was purified by flash chromatography (ethyl acetate/petroleum ether = 1:12) to afford the product **13** as a white solid in 64% yield (50 mg. mp = 167–168 °C; ^1^H NMR (400 MHz, CDCl_3_): δ 11.33 (s, 1H), 8.29 (d, *J* = 1.6 Hz, 1H), 7.81 (d, *J* = 1.0 Hz, 1H), 7.72 (d, *J* = 7.0 Hz, 2H), 7.66 (dd, *J* = 8.4, 1.7 Hz, 1H), 7.59–7.47 (m, 5H), 7.38 (dd, *J* = 6.0 Hz, 1.7 Hz, 4H), 7.34 (d, *J* = 1.2 Hz, 1H); ^13^C{^1^H} NMR (100 MHz, CDCl_3_): δ 165.1, 162.8, 150.7, 150.4, 139.4, 134.8, 133.8, 131.8, 129.3, 129.2, 128.8, 128.6, 127.6, 126.8, 122.8, 120.7, 118.7, 118.2, 115.5, 111.5, 105.0, 90.2, 88.1; HRMS (ESI-TOF) *m*/*z*: [M + H]^+^ calcd for C_27_H_16_O_3_, 389.1172; found, 389.1185.

### 7-Hydroxy-9-phenyl-6*H*-benzo[*c*]chromen-6-one-8,10-d_2_ (Compound **3a**-*d*_2_)

Following the general procedure, the reaction of 2-(3-phenyl-1-(piperidin-1-yl)prop-2-yn-1-yl)phenol (**1a**) (0.2 mmol, 1.0 equiv) with 1-(4-ethoxy-2,4-dioxobutyl)pyridin-1-ium bromide (**2a**) (0.2 mmol, 1.0 equiv), DBU (0.3 mmol, 1.5 equiv), and methanol-*d*_*4*_ (100 μL) using undried acetonitrile (2 mL) as solvent at 120 °C (oil bath) for 12 h afforded product **3a***-d*_*2*_ as a white solid in 72% yield (42 mg). mp = 202–203 °C; ^1^H NMR (400 MHz, CDCl_3_): δ 11.40 (s, 1H), 8.13 (d, *J* = 8.0 Hz, 1H), 7.80 (s, 0.5H), 7.70 (d, *J* = 7.2 Hz, 2H), 7.56–7.47 (m, 4H), 7.43–7.37 (m, 2H), 7.31 (s, 0.5H); ^13^C{^1^H} NMR (100 MHz, CDCl_3_): δ 165.5, 162.8, 150.9, 150.4, 139.6, 135.6, 130.8, 129.2, 129.1, 127.5, 125.3, 123.5, 118.5, 118.0, 115.1, 111.3, 105.0; HRMS (ESI-TOF) *m*/*z*: [M + H]^+^ calcd for C_19_H_11_D_2_O_3_, 291.0985; found, 291.0983.

### Gram-Scale Synthesis of compound **3d**

Following the general procedures, the reaction of 4-bromo-2-(3-phenyl-1-(piperidin-1-yl)prop-2-yn-1-yl)phenol (**1d**) (5 mmol, 1.0 equiv) with 1-(4-ethoxy-2,4-dioxobutyl)pyridin-1-ium bromide (**2a**) (5 mmol, 1.0 equiv), and DBU (7.5 mmol, 1.5 equiv) using undried acetonitrile (15 mL) as solvent at 120 °C (oil bath) for 12 h afforded product **3d** as a white solid in 78% yield (1.42 g).

## Data Availability

The data underlying this study are available in the published article and its Supporting Information.

## Supporting Information Available

Experimental details, Copies of ^1^H NMR, ^13^C NMR, ^31^P NMR,^19^F NMR spectra, and X-ray crystallographic data for product **3a** (PDF)

## Accession Codes

CCDC 2295942 contains the supplementary crystallographic data for this paper. These data can be obtained free of charge via www.ccdc.cam.ac.uk/data_request/cif, or by emailing data_request@ccdc.cam.ac.uk, or by contactig The Cambridge Crystallographic Data Centre.
