# Supplementary material for: Cascade Annulation Strategy for Expeditious Assembly of Hydroxybenzo[c]chromen-6-ones and Their Photophysical Property Studies
Source: J Org Chem. 2023 Nov 18;88(23):16609–20. doi: 10.1021/acs.joc.3c02188 (PMC11966774; doi:10.1021/acs.joc.3c02188)

Supporting Information

Cascade Annulation Strategy for Expeditious Assembly of Hydroxybenzo[*c*]chromen-6-ones and Their Photophysical Property Studies

Yanan Liu,[†] Pui Ying Choy,[‡] Demao Wang,[†] Mengdi Wu,[†] Qiang Tang,[†] Xinwei He,[†,*] Yongjia Shang,[†,*] Fuk Yee Kwong[‡,*]

[†]Key Laboratory of Functional Molecular Solids, Ministry of Education, Anhui Laboratory of Molecule-Based Materials (State Key Laboratory Cultivation Base), College of Chemistry and Materials Science, Anhui Normal University, Wuhu 241000, P.R. China

[‡]Department of Chemistry and State Key Laboratory of Synthetic Chemistry, The Chinese University of Hong Kong, New Territories, Shatin, Hong Kong, P.R. China.

Email: xinweihe@mail.ahnu.edu.cn, shyj@mail.ahnu.edu.cn, fykwong@cuhk.edu.hk

Table of Content

1. GC-MS Spectra for Mechanistic Investigations S2
2. X-ray Crystallographic Data of Product **3a**....................S5
3. ^{1}H, ^{13}C, ^{31}P and ^{19}F NMR Spectra S6

1. GC-MS Spectra for Mechanistic Investigations

Intermediate *o*-AQM

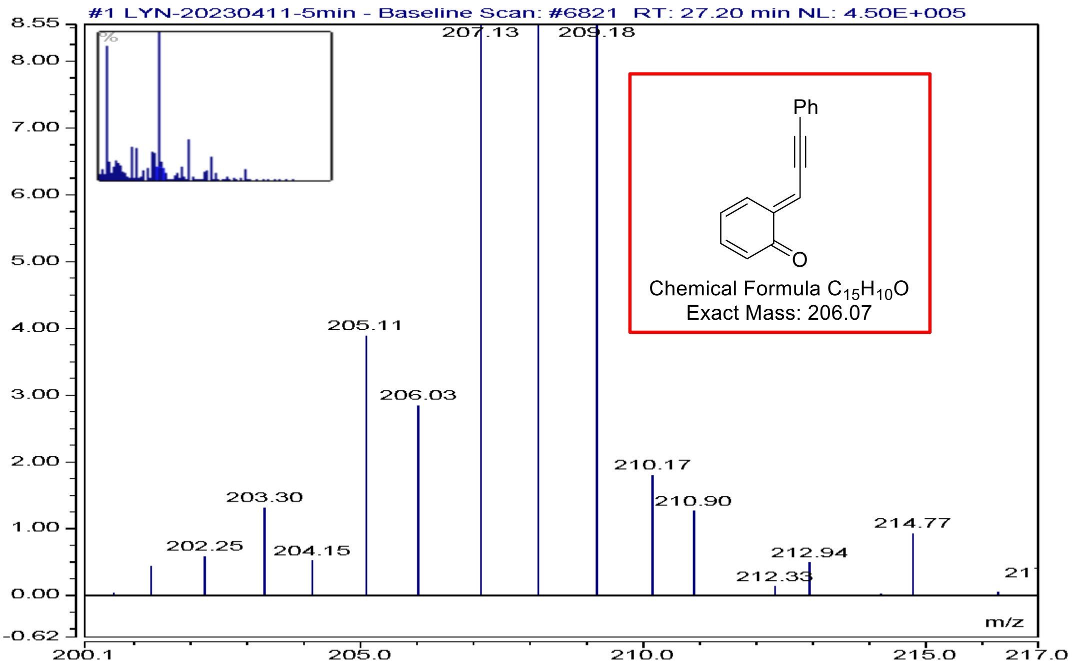

Figure S1. GC-MS Spectra of possibly intermediate *o*-AQM

Intermediate A

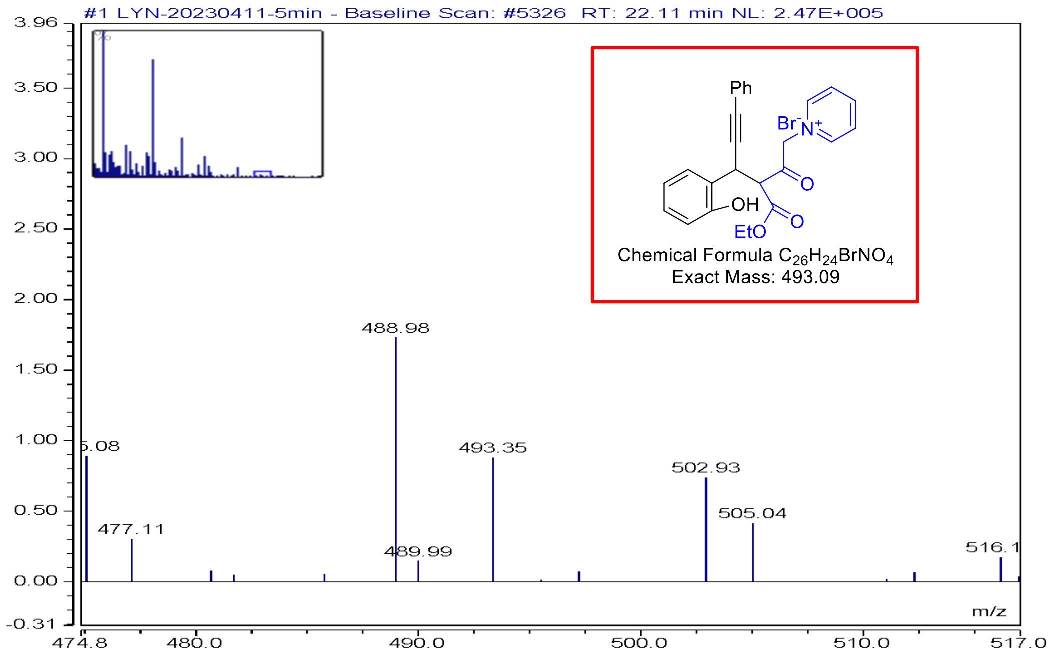

Figure S2. GC-MS Spectra of possibly intermediate A

Intermediate B

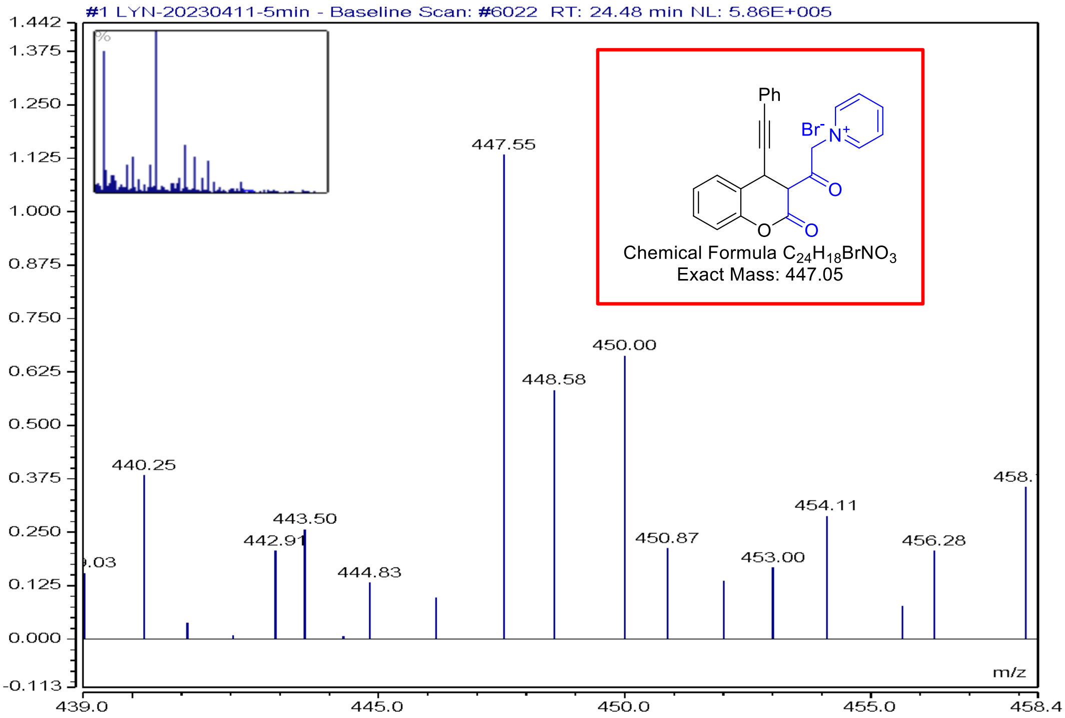

Figure S3. GC-MS Spectra of possibly intermediate B

Intermediate C

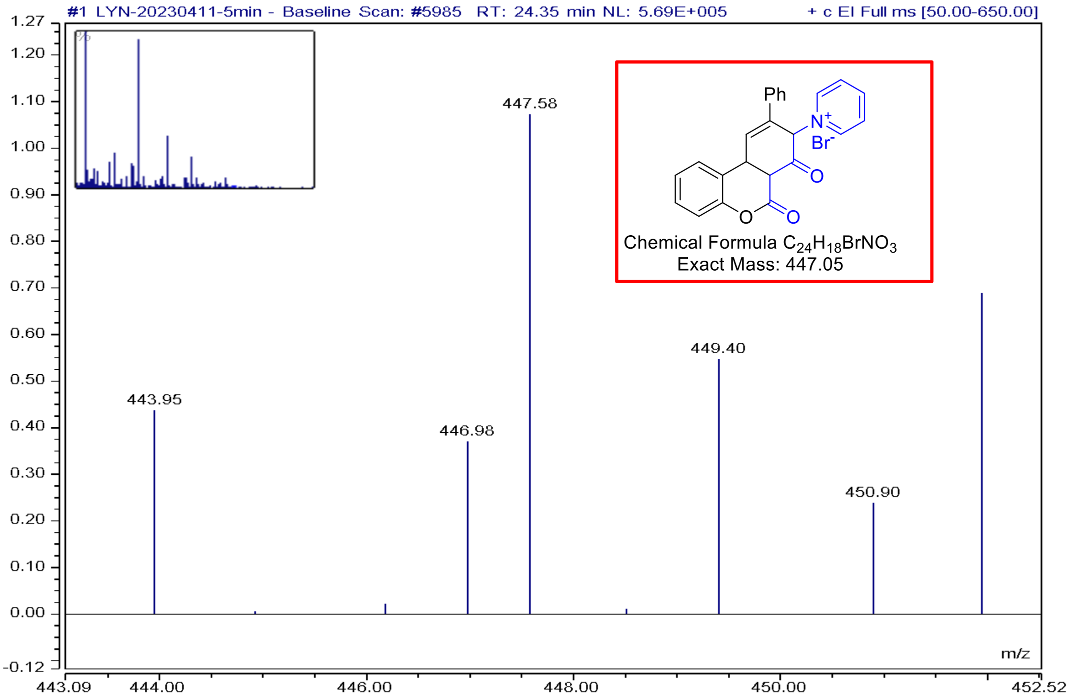

Figure S4. GC-MS Spectra of possibly intermediate C

Intermediate D

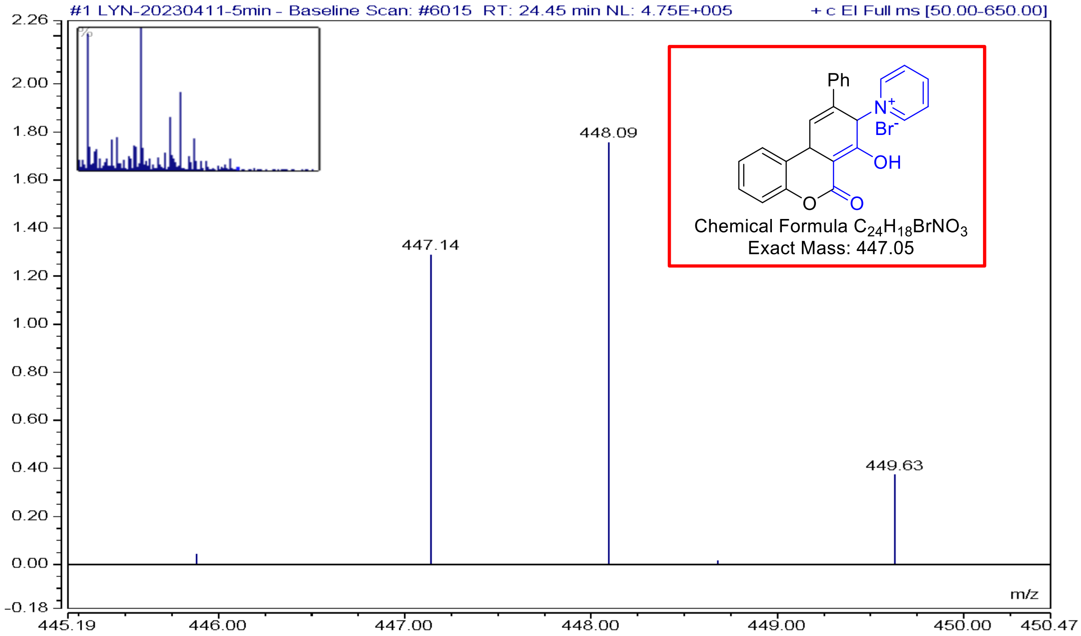

Figure S5. GC-MS Spectra of possibly intermediate D

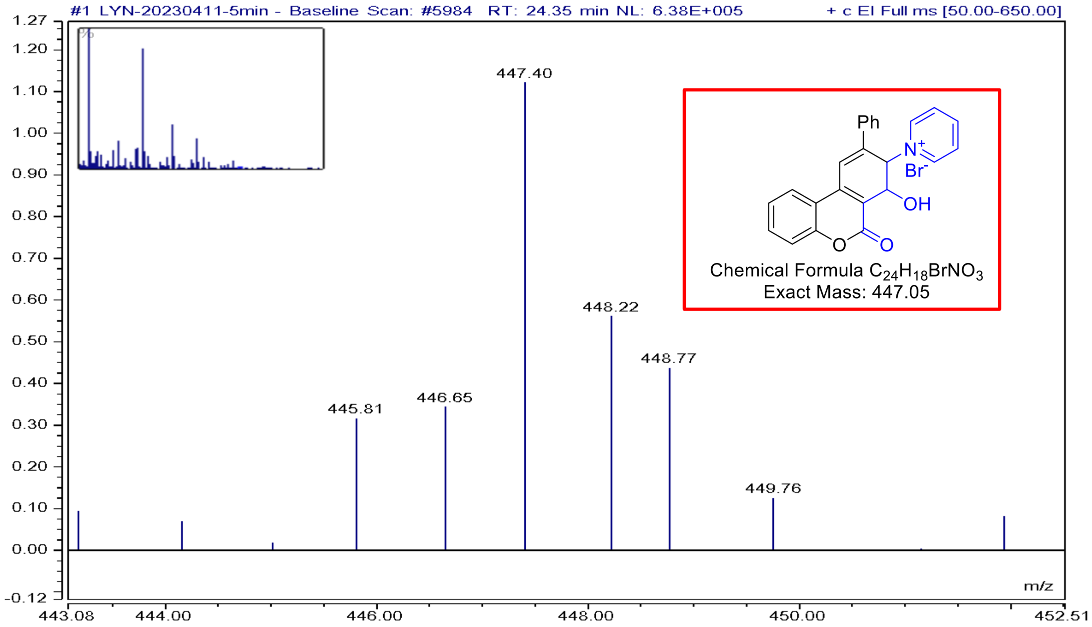

Figure S6. GC-MS Spectra of possibly intermediate from intermediate D through 1,3-H shift process

2. X-ray Crystallographic Data of Product 3a

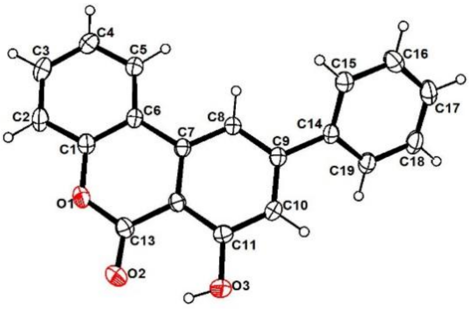

Figure S7. ORTEP drawing of compound **3a**. All hydrogen atoms have been omitted for clarity (CCDC 2295942) (30% probability for the thermal ellipsoid).

The purified compound **3a** is dissolved in a mixed solvent of ethyl acetate and petroleum ether, and placed in a dark cabinet to slowly evaporate. After several days, a colourless bulk crystal was obtained. The X-ray crystal-structure determinations were obtained on a Bruker Smart CCDC APEX-2 diffractometer (graphite- monochromated Mo *Kα* radiation, *λ*=0.71073 nm) at 300 K.

Table S1. Crystal data and structure refinement for product **3a**

CCDC number	2295942	
Identification code	20221007b	
Empirical formula	C19 H12 O3	
Formula weight	288.29	
Temperature	300 K	
Radiation	MoKα (λ = 0.71073 Å)	
Crystal system	Monoclinic	
Space group	$P2_1/c$	
Unit cell dimensions	a = 11.5189(17) Å	α= 90°
	b = 15.798(2) Å	β= 92.516(5)°
	c = 7.7319(9) Å	γ= 90°
Volume	1340.2(3) $Å^3$	
Z	4	
Density (calculated)	1.429 g/cm^3	
μ	0.097 mm^{-1}	
F(000)	600.0	
Crystal size	0.28 × 0.25 × 0.23 mm^3	
2Θ range for data collection	6.104 to 55.742°	
Index ranges	$-15 \leq h \leq 15$, $-20 \leq k \leq 20$, $-9 \leq l \leq 9$	
Reflections collected	47478	
Independent reflections	3144[R_{int} = 0.0649, R_{sigma} = 0.0348]	
Data/restraints/parameters	3144 / 0 / 201	
Goodness-of-fit on F^2	1.032	
Final R indices [I>2σ (I)]	R_1 = 0.0399, wR_2 = 0.1037	
Final R indices (all data)	R_1 = 0.0560, wR_2 = 0.1152	
Largest diff. peak/hole	0.22 and -0.17 e.$Å^{-3}$	

3. ^{1}H, ^{13}C, ^{31}P and ^{19}F NMR Spectra

1-(4-Ethoxy-2,4-dioxobutyl)pyridin-1-ium bromide (compound 2a)

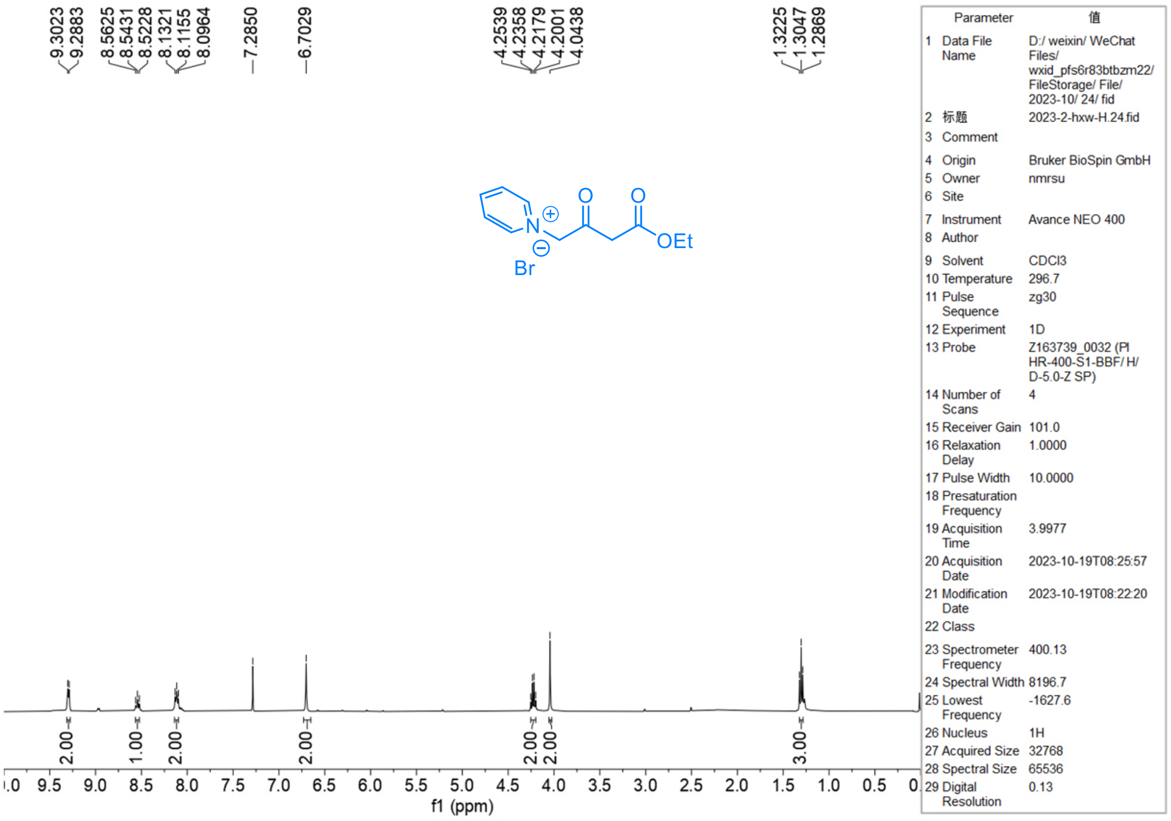

Parameter	值
1 Data File Name	D:/ weixin/ WeChat Files/ wxid_pfs6r83btbzm22/ FileStorage/ File/ 2023-10/ 24/ fid
2 标题	2023-2-hxw-H.24.fid
3 Comment	
4 Origin	Bruker BioSpin GmbH
5 Owner	nmrsu
6 Site	
7 Instrument	Avance NEO 400
8 Author	
9 Solvent	CDCl3
10 Temperature	296.7
11 Pulse Sequence	zg30
12 Experiment	1D
13 Probe	Z163739_0032 (PI HR-400-S1-BBF/ H/ D-5.0-Z SP)
14 Number of Scans	4
15 Receiver Gain	101.0
16 Relaxation Delay	1.0000
17 Pulse Width	10.0000
18 Presaturation Frequency	
19 Acquisition Time	3.9977
20 Acquisition Date	2023-10-19T08:25:57
21 Modification Date	2023-10-19T08:22:20
22 Class	
23 Spectrometer Frequency	400.13
24 Spectral Width	8196.7
25 Lowest Frequency	-1627.6
26 Nucleus	1H
27 Acquired Size	32768
28 Spectral Size	65536
29 Digital Resolution	0.13

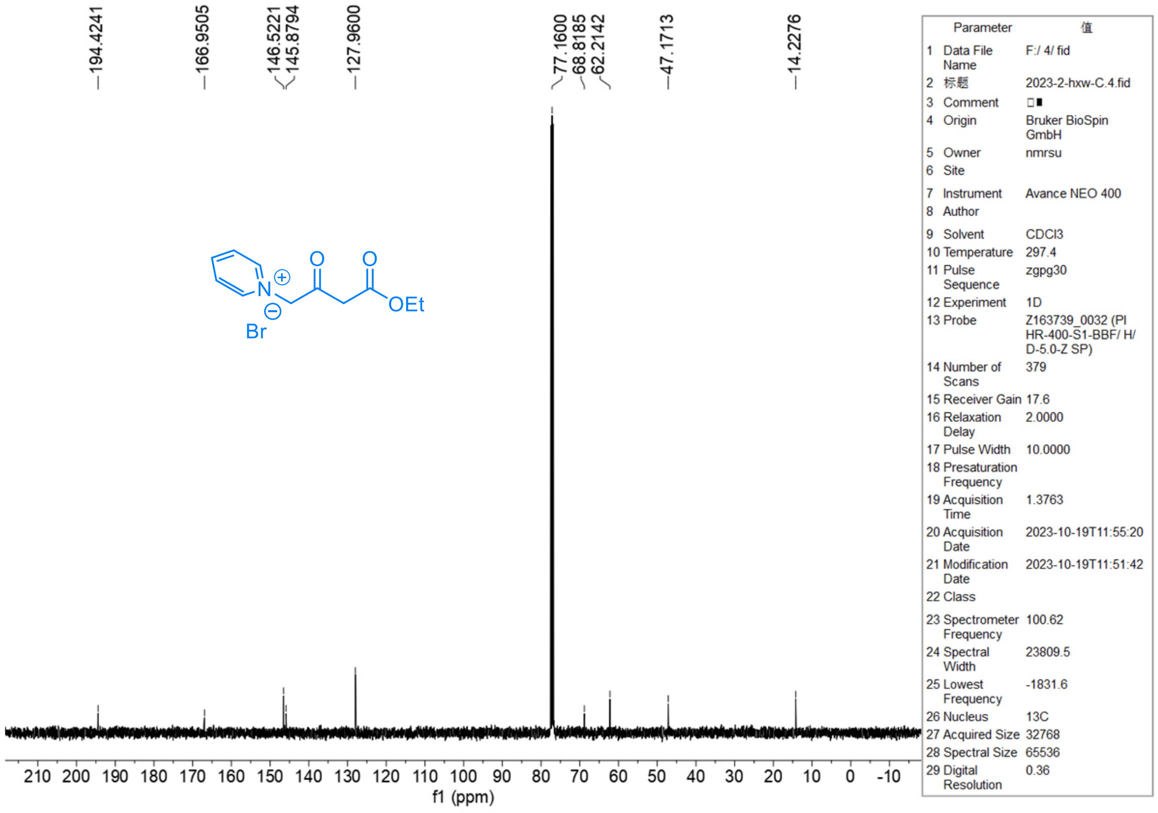

Parameter	值
1 Data File Name	F:/ 4/ fid
2 标题	2023-2-hxw-C.4.fid
3 Comment	□■
4 Origin	Bruker BioSpin GmbH
5 Owner	nmrsu
6 Site	
7 Instrument	Avance NEO 400
8 Author	
9 Solvent	CDCl3
10 Temperature	297.4
11 Pulse Sequence	zgpg30
12 Experiment	1D
13 Probe	Z163739_0032 (PI HR-400-S1-BBF/ H/ D-5.0-Z SP)
14 Number of Scans	379
15 Receiver Gain	17.6
16 Relaxation Delay	2.0000
17 Pulse Width	10.0000
18 Presaturation Frequency	
19 Acquisition Time	1.3763
20 Acquisition Date	2023-10-19T11:55:20
21 Modification Date	2023-10-19T11:51:42
22 Class	
23 Spectrometer Frequency	100.62
24 Spectral Width	23809.5
25 Lowest Frequency	-1831.6
26 Nucleus	13C
27 Acquired Size	32768
28 Spectral Size	65536
29 Digital Resolution	0.36

7-Hydroxy-9-phenyl-6*H*-benzo[*c*]chromen-6-one (product 3a)

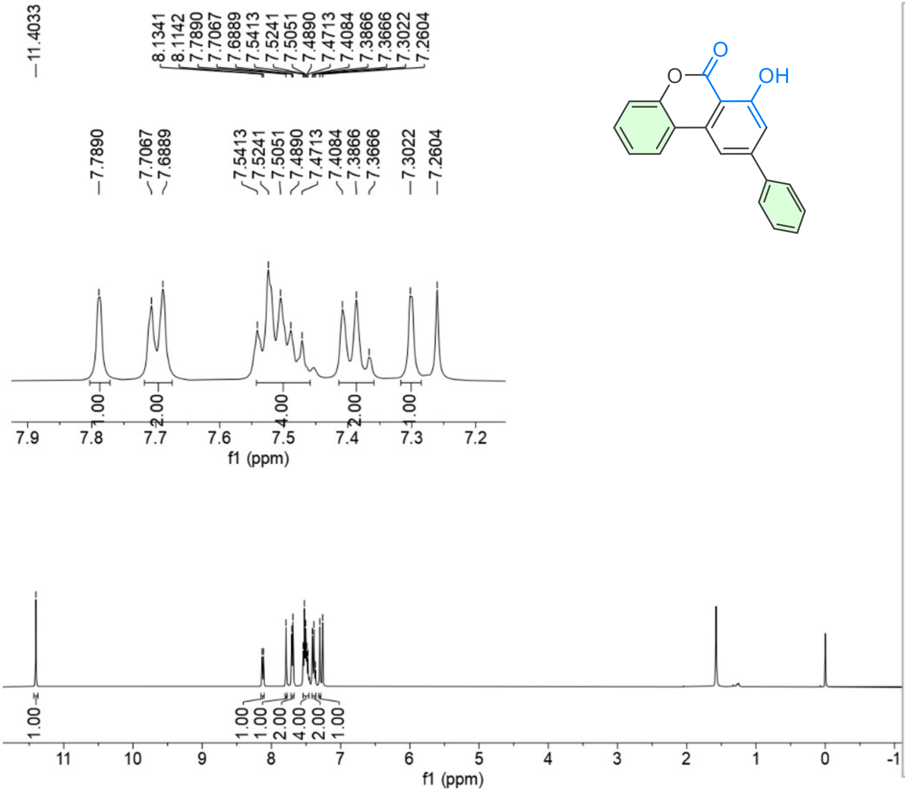

	Parameter	值
1	Data File Name	F:/ hxw2022/ 400-new/ 22-1-hxw-H/ 183/ fid
2	标题	22-1-hxw-H.183.fid
3	Comment	
4	Origin	Bruker BioSpin GmbH
5	Owner	nmrsu
6	Site	
7	Instrument	Avance NEO 400
8	Author	
9	Solvent	CDCl3
10	Temperature	292.7
11	Pulse Sequence	zg30
12	Experiment	1D
13	Probe	Z163739_0511 (PI HR-BBO400S1-BBF/ H/ D-5.0-Z SP)
14	Number of Scans	4
15	Receiver Gain	101.0
16	Relaxation Delay	1.0000
17	Pulse Width	8.0000
18	Presaturation Frequency	
19	Acquisition Time	3.9977
20	Acquisition Date	2022-09-21T11:29:12
21	Modification Date	2022-09-21T11:29:16
22	Class	
23	Spectrometer Frequency	400.15
24	Spectral Width	8196.7
25	Lowest Frequency	-1637.0
26	Nucleus	1H
27	Acquired Size	32768
28	Spectral Size	65536

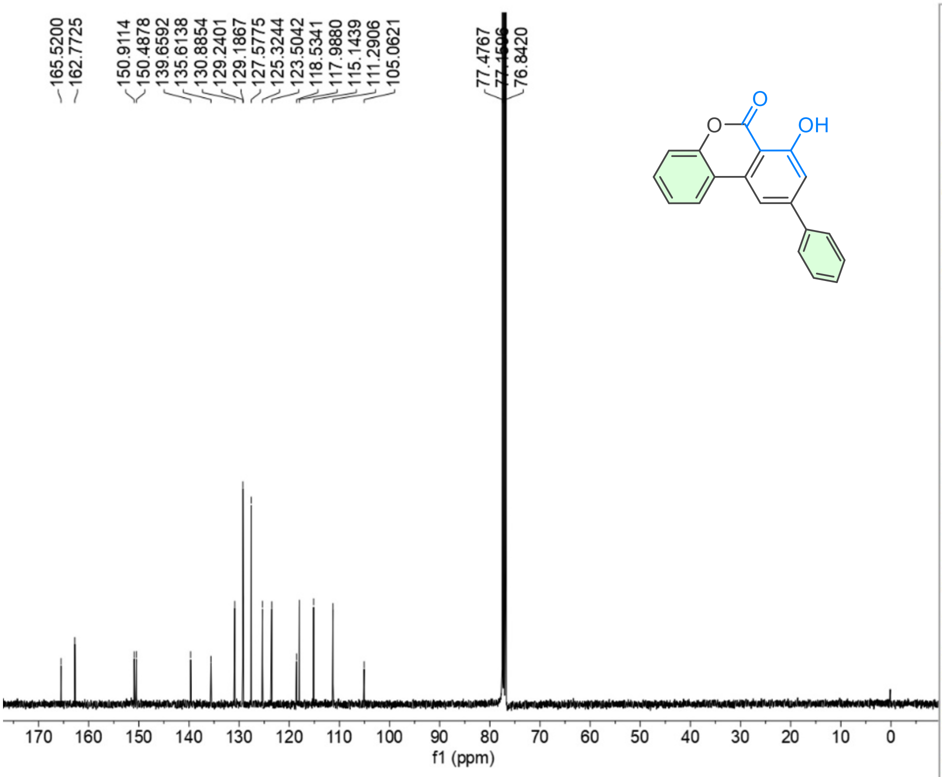

	Parameter	值
1	Data File Name	F:/ hxw2022/ 400-new/ 22-1-hxw-C/ 81/ fid
2	标题	22-1-hxw-C. 81.fid
3	Comment	
4	Origin	Bruker BioSpin GmbH
5	Owner	nmrsu
6	Site	
7	Instrument	Avance NEO 400
8	Author	
9	Solvent	CDCl3
10	Temperature	293.9
11	Pulse Sequence	zgpg30
12	Experiment	1D
13	Probe	Z163739_0511 (PI HR-BBO400S1-BBF/ H/ D-5.0-Z SP)
14	Number of Scans	800
15	Receiver Gain	13.3
16	Relaxation Delay	2.0000
17	Pulse Width	8.0000
18	Presaturation Frequency	
19	Acquisition Time	1.3763
20	Acquisition Date	2022-09-22T12:30:12
21	Modification Date	2022-09-22T12:30:22
22	Class	
23	Spectrometer Frequency	100.63
24	Spectral Width	23809.5
25	Lowest Frequency	-1831.3
26	Nucleus	13C
27	Acquired Size	32768
28	Spectral Size	65536

2-Fluoro-7-hydroxy-9-phenyl-6*H*-benzo[*c*]chromen-6-one (product 3b)

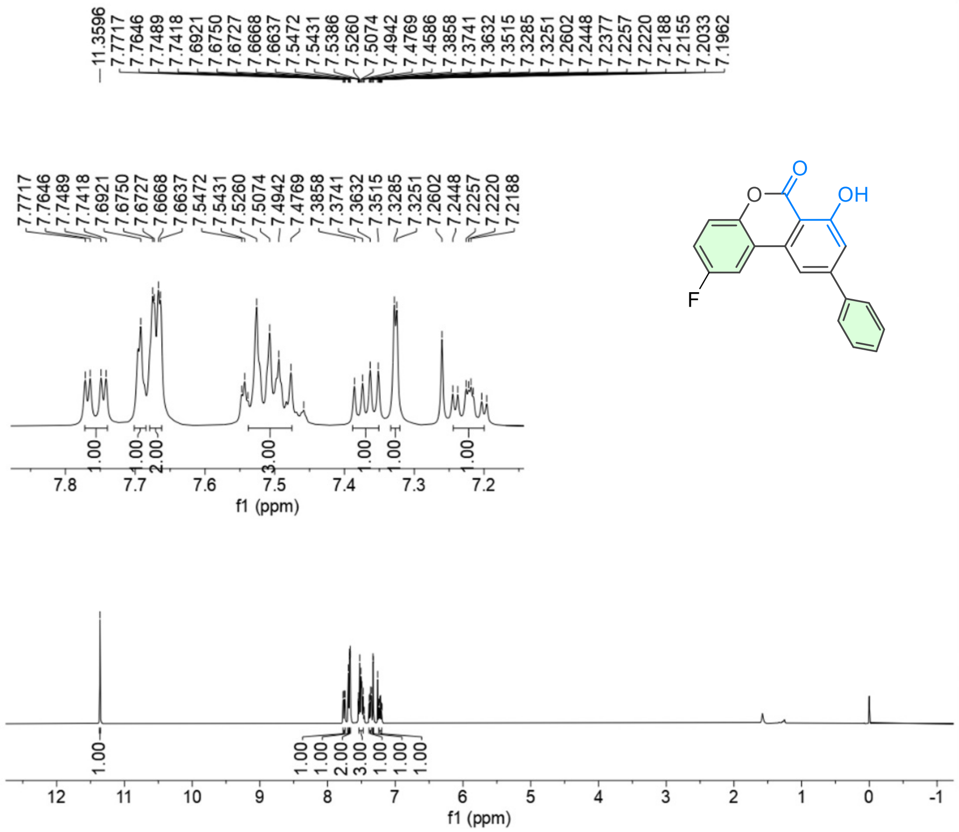

Parameter	值
1 Data File Name	F:/ hxw2022/ 400-new/ 22-2-hxw-H/ 47/ fid
2 标题	22-2-hxw-H. 47. fid
3 Comment	
4 Origin	Bruker BioSpin GmbH
5 Owner	nmrsu
6 Site	
7 Instrument	Avance NEO 400
8 Author	
9 Solvent	CDCl3
10 Temperature	295.1
11 Pulse Sequence	zg30
12 Experiment	1D
13 Probe	Z163739_0511 (PI HR-BB0400S1-BBF/ H/ D-5.0-Z SP)
14 Number of Scans	4
15 Receiver Gain	101.0
16 Relaxation Delay	1.0000
17 Pulse Width	8.0000
18 Presaturation Frequency	
19 Acquisition Time	3.9977
20 Acquisition Date	2022-10-11T17:47:02
21 Modification Date	2022-10-11T17:47:06
22 Class	
23 Spectrometer Frequency	400.15
24 Spectral Width	8196.7
25 Lowest Frequency	-1637.0
26 Nucleus	1H
27 Acquired Size	32768
28 Spectral Size	65536

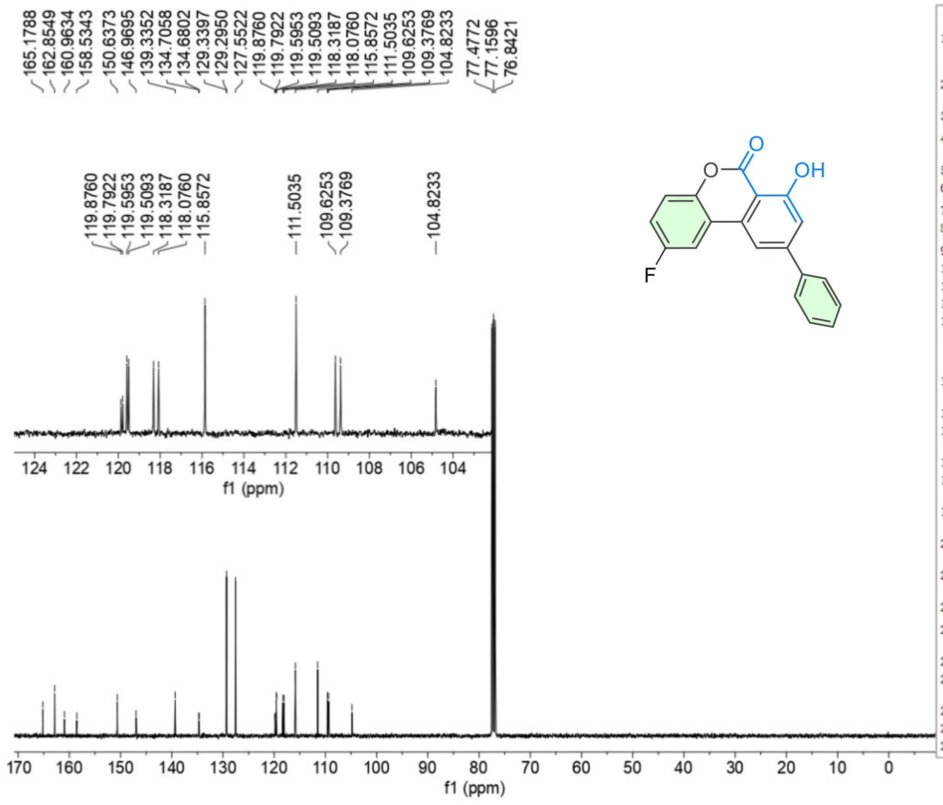

Parameter	值
1 Data File Name	F:/ hxw2022/ 400-new/ 22-2-hxw-C/ 26/ fid
2 标题	22-2-hxw-C. 26. fid
3 Comment	
4 Origin	Bruker BioSpin GmbH
5 Owner	nmrsu
6 Site	
7 Instrument	Avance NEO 400
8 Author	
9 Solvent	CDCl3
10 Temperature	295.8
11 Pulse Sequence	zgpg30
12 Experiment	1D
13 Probe	Z163739_0511 (PI HR-BB0400S1-BBF/ H/ D-5.0-Z SP)
14 Number of Scans	500
15 Receiver Gain	15.4
16 Relaxation Delay	2.0000
17 Pulse Width	8.0000
18 Presaturation Frequency	
19 Acquisition Time	1.3763
20 Acquisition Date	2022-10-12T01:37:05
21 Modification Date	2022-10-12T01:37:08
22 Class	
23 Spectrometer Frequency	100.63
24 Spectral Width	23809.5
25 Lowest Frequency	-1799.0
26 Nucleus	13C
27 Acquired Size	32768
28 Spectral Size	65536

2-Fluoro-7-hydroxy-9-phenyl-6*H*-benzo[*c*]chromen-6-one (product 3b)

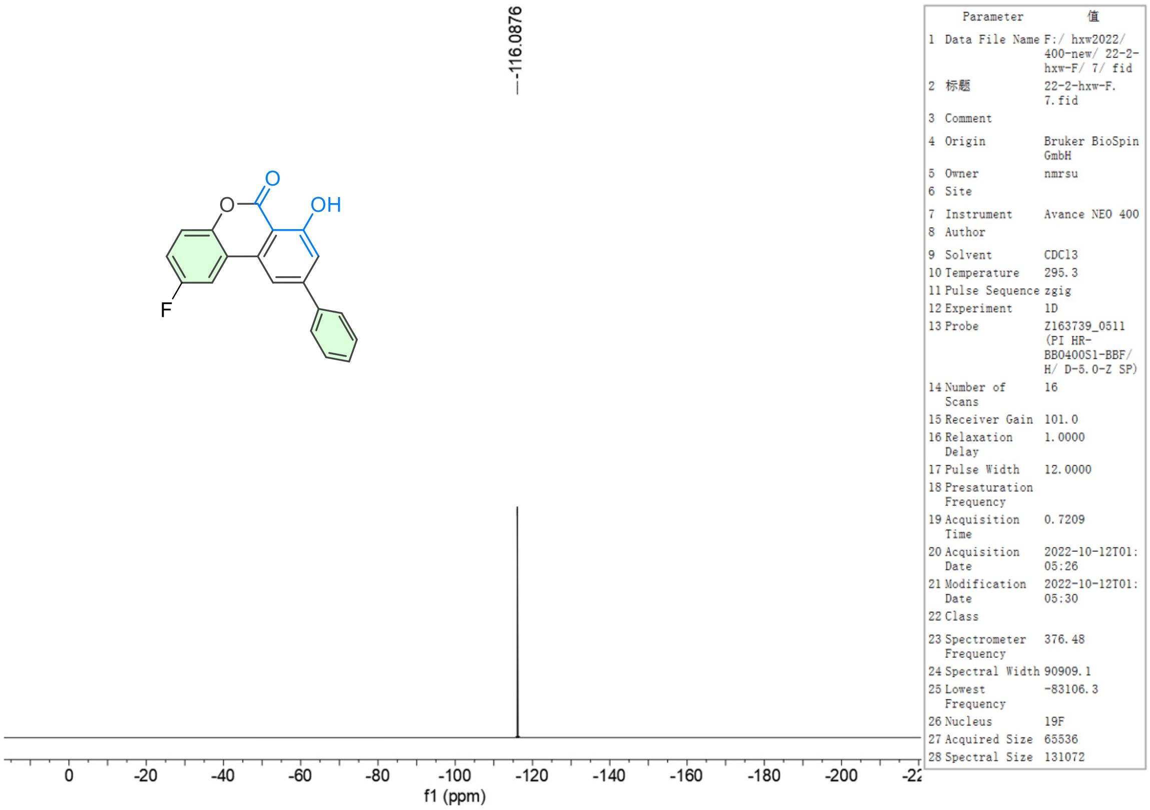

	Parameter	值
1	Data File Name	F:/ hxw2022/ 400-new/ 22-2-hxw-F/ 7/ fid
2	标题	22-2-hxw-F. 7.fid
3	Comment	
4	Origin	Bruker BioSpin GmbH
5	Owner	nmrsu
6	Site	
7	Instrument	Avance NEO 400
8	Author	
9	Solvent	CDCl3
10	Temperature	295.3
11	Pulse Sequence	zgig
12	Experiment	1D
13	Probe	Z163739_0511 (PI HR-BB0400S1-BBF/ H/ D-5.0-Z SP)
14	Number of Scans	16
15	Receiver Gain	101.0
16	Relaxation Delay	1.0000
17	Pulse Width	12.0000
18	Presaturation Frequency	
19	Acquisition Time	0.7209
20	Acquisition Date	2022-10-12T01:05:26
21	Modification Date	2022-10-12T01:05:30
22	Class	
23	Spectrometer Frequency	376.48
24	Spectral Width	90909.1
25	Lowest Frequency	-83106.3
26	Nucleus	19F
27	Acquired Size	65536
28	Spectral Size	131072

2-Chloro-7-hydroxy-9-phenyl-6*H*-benzo[*c*]chromen-6-one (product 3c)

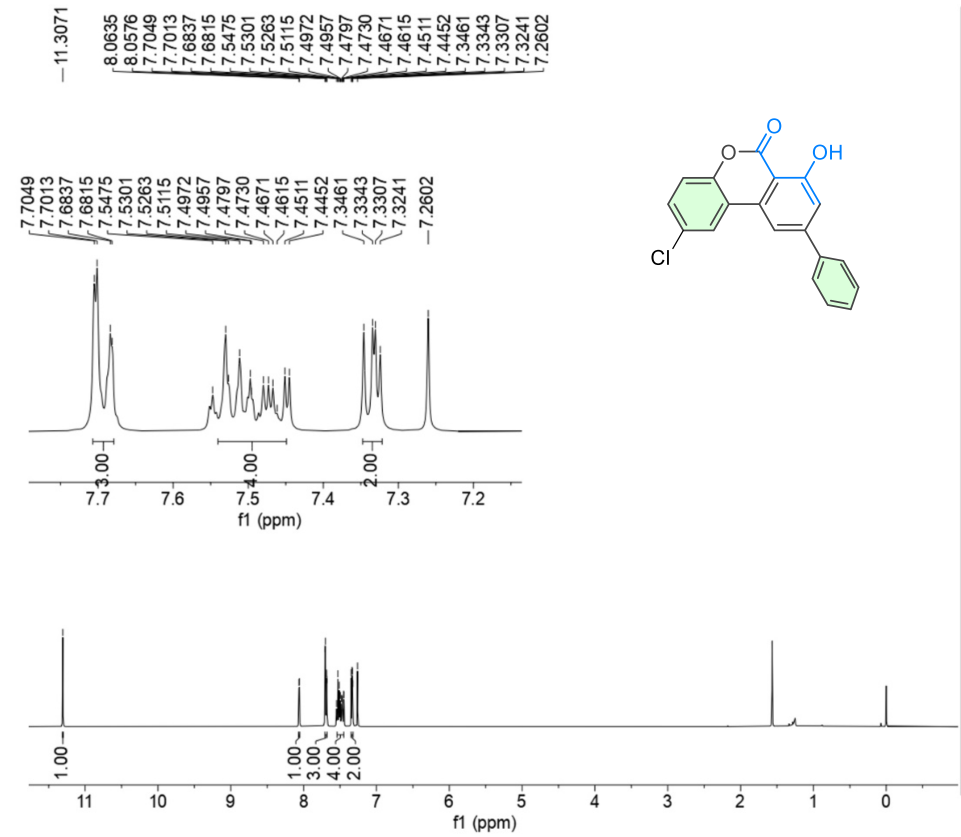

Parameter	值
1 Data File Name	F:/ hxw2022/ 400-new/ 22-2-hxw-H/ 35/ fid
2 标题	22-2-hxw-H. 35. fid
3 Comment	
4 Origin	Bruker BioSpin GmbH
5 Owner	nmrsu
6 Site	
7 Instrument	Avance NEO 400
8 Author	
9 Solvent	CDCl3
10 Temperature	295. 0
11 Pulse Sequence	zg30
12 Experiment	1D
13 Probe	Z163739_0511 (PI HR-BBO400S1-BBF/ H/ D-5. 0-Z SP)
14 Number of Scans	4
15 Receiver Gain	101. 0
16 Relaxation Delay	1. 0000
17 Pulse Width	8. 0000
18 Presaturation Frequency	
19 Acquisition Time	3. 9977
20 Acquisition Date	2022-10-10T10: 50:00
21 Modification Date	2022-10-10T10: 50:04
22 Class	
23 Spectrometer Frequency	400. 15
24 Spectral Width	8196. 7
25 Lowest Frequency	-1637. 0
26 Nucleus	1H
27 Acquired Size	32768
28 Spectral Size	65536

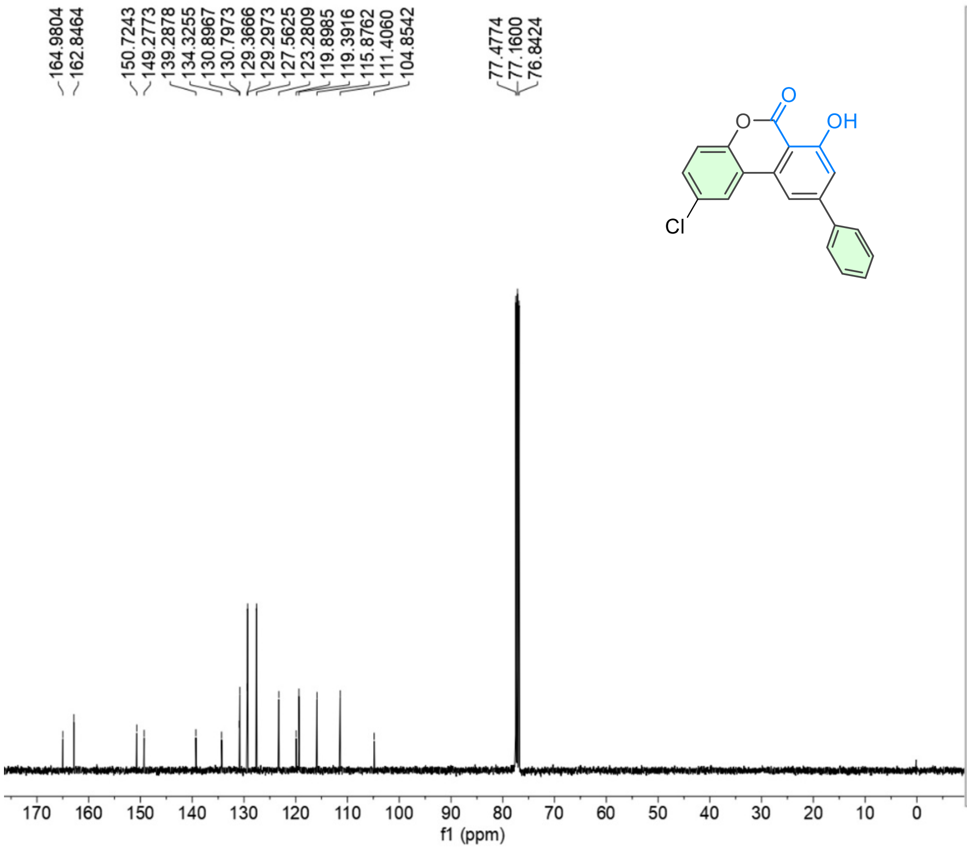

Parameter	值
1 Data File Name	F:/ hxw2022/ 400-new/ 22-2-hxw-C/ 49/ fid
2 标题	22-2-hxw-C. 49. fid
3 Comment	
4 Origin	Bruker BioSpin GmbH
5 Owner	nmrsu
6 Site	
7 Instrument	Avance NEO 400
8 Author	
9 Solvent	CDCl3
10 Temperature	295. 9
11 Pulse Sequence	zgpg30
12 Experiment	1D
13 Probe	Z163739_0511 (PI HR-BBO400S1-BBF/ H/ D-5. 0-Z SP)
14 Number of Scans	200
15 Receiver Gain	15. 0
16 Relaxation Delay	2. 0000
17 Pulse Width	8. 0000
18 Presaturation Frequency	
19 Acquisition Time	1. 3763
20 Acquisition Date	2022-10-18T17: 55:03
21 Modification Date	2022-10-18T17: 55:12
22 Class	
23 Spectrometer Frequency	100. 63
24 Spectral Width	23809. 5
25 Lowest Frequency	-1798. 9
26 Nucleus	13C
27 Acquired Size	32768
28 Spectral Size	65536

2-Bromo-7-hydroxy-9-phenyl-6*H*-benzo[*c*]chromen-6-one (product 3d)

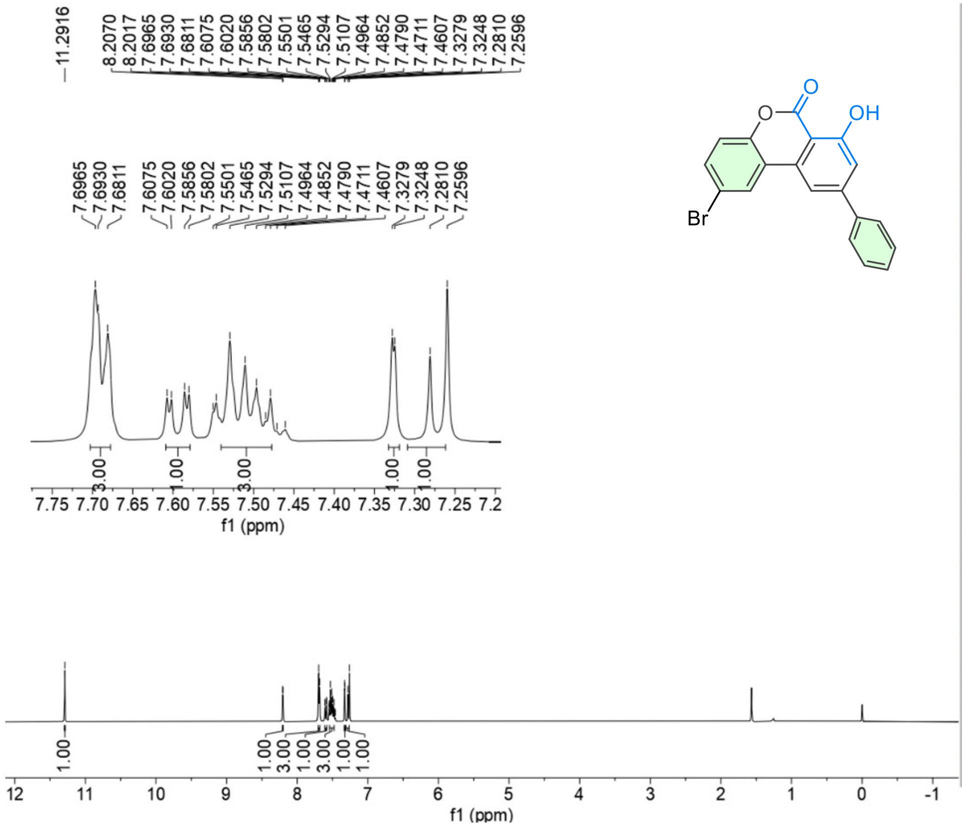

Parameter		值
1	Data File Name	F:/ hxw2022/ 400-new/ 22-2-hxw-H/ 32/ fid
2	标题	22-2-hxw-H. 32. fid
3	Comment	
4	Origin	Bruker BioSpin GmbH
5	Owner	nmrsu
6	Site	
7	Instrument	Avance NEO 400
8	Author	
9	Solvent	CDCl3
10	Temperature	295. 0
11	Pulse Sequence	zg30
12	Experiment	1D
13	Probe	Z163739_0511 (PI HR-BBO400S1-BBF/ H/ D-5. 0-Z SP)
14	Number of Scans	4
15	Receiver Gain	101. 0
16	Relaxation Delay	1. 0000
17	Pulse Width	8. 0000
18	Presaturation Frequency	
19	Acquisition Time	3. 9977
20	Acquisition Date	2022-10-10T10: 22:10
21	Modification Date	2022-10-10T10: 22:14
22	Class	
23	Spectrometer Frequency	400. 15
24	Spectral Width	8196. 7
25	Lowest Frequency	-1637. 0
26	Nucleus	1H
27	Acquired Size	32768
28	Spectral Size	65536

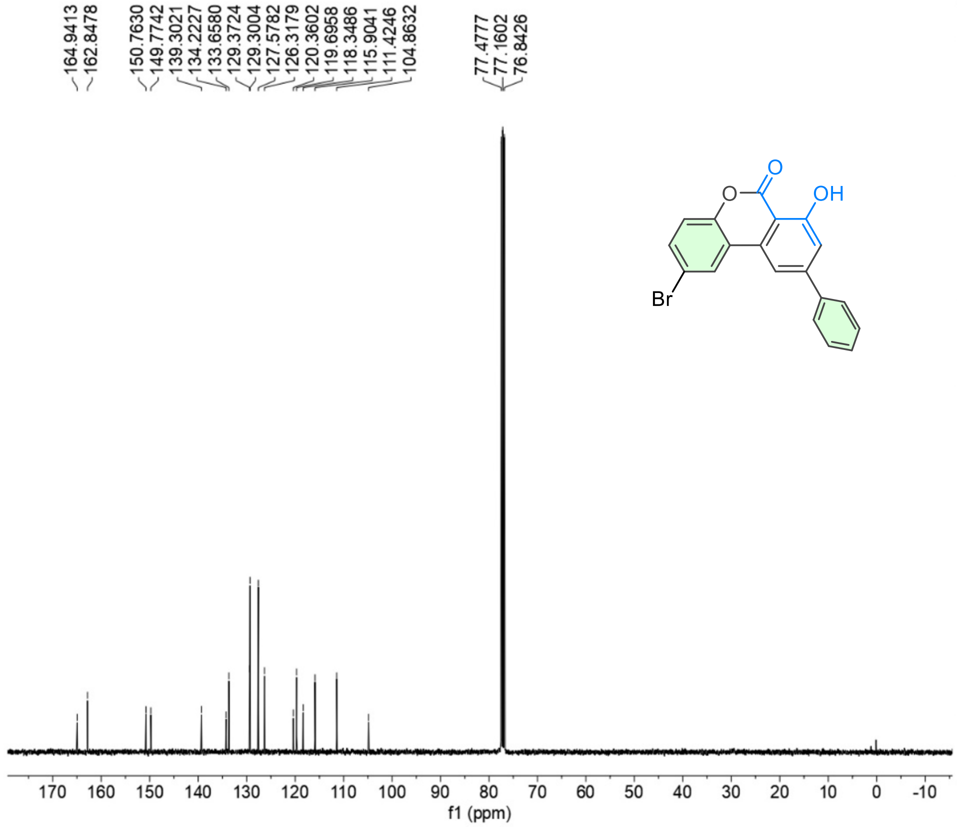

Parameter		值
1	Data File Name	F:/ hxw2022/ 400-new/ 22-2-hxw-C/ 66/ fid
2	标题	22-2-hxw-C. 66. fid
3	Comment	
4	Origin	Bruker BioSpin GmbH
5	Owner	nmrsu
6	Site	
7	Instrument	Avance NEO 400
8	Author	
9	Solvent	CDCl3
10	Temperature	295. 8
11	Pulse Sequence	zgpg30
12	Experiment	1D
13	Probe	Z163739_0511 (PI HR-BBO400S1-BBF/ H/ D-5. 0-Z SP)
14	Number of Scans	700
15	Receiver Gain	16. 2
16	Relaxation Delay	2. 0000
17	Pulse Width	8. 0000
18	Presaturation Frequency	
19	Acquisition Time	1. 3763
20	Acquisition Date	2022-10-21T05: 13:39
21	Modification Date	2022-10-21T05: 13:44
22	Class	
23	Spectrometer Frequency	100. 63
24	Spectral Width	23809. 5
25	Lowest Frequency	-1798. 8
26	Nucleus	13C
27	Acquired Size	32768
28	Spectral Size	65536

7-Hydroxy-2-iodo-9-phenyl-6*H*-benzo[*c*]chromen-6-one (product 3e)

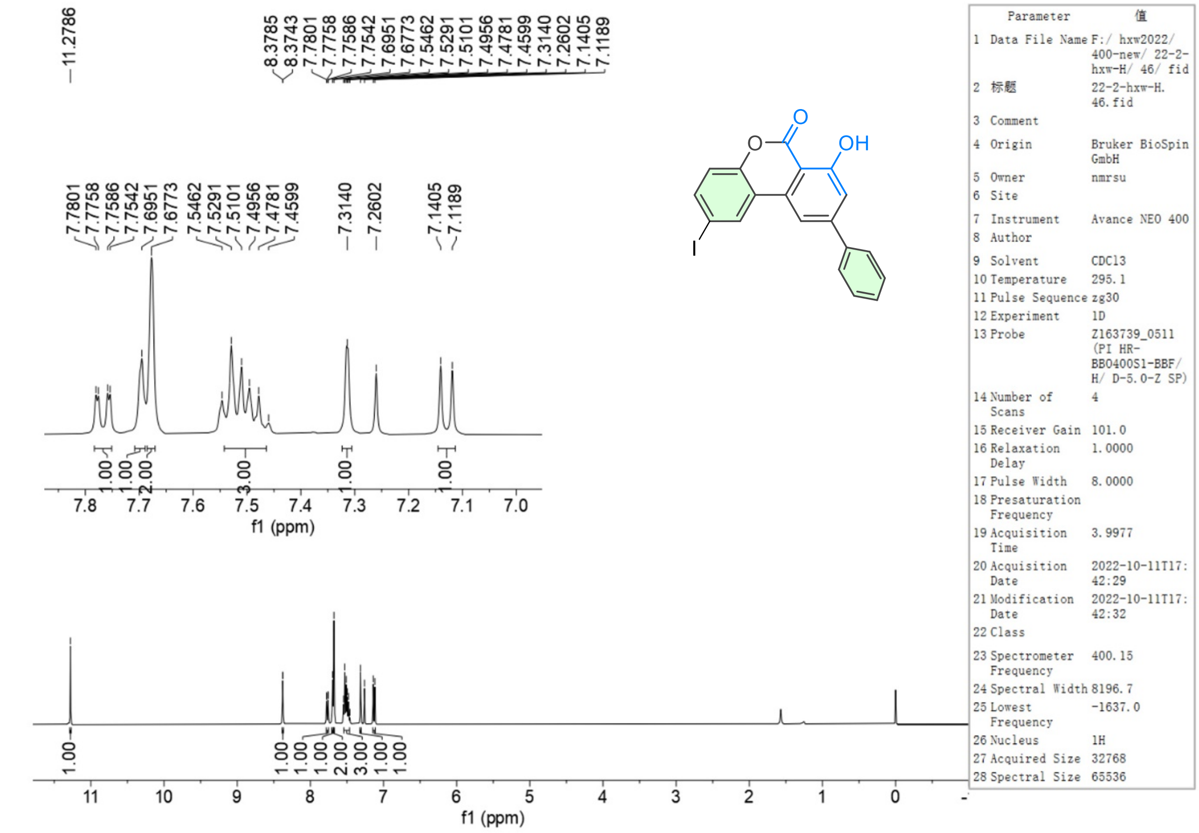

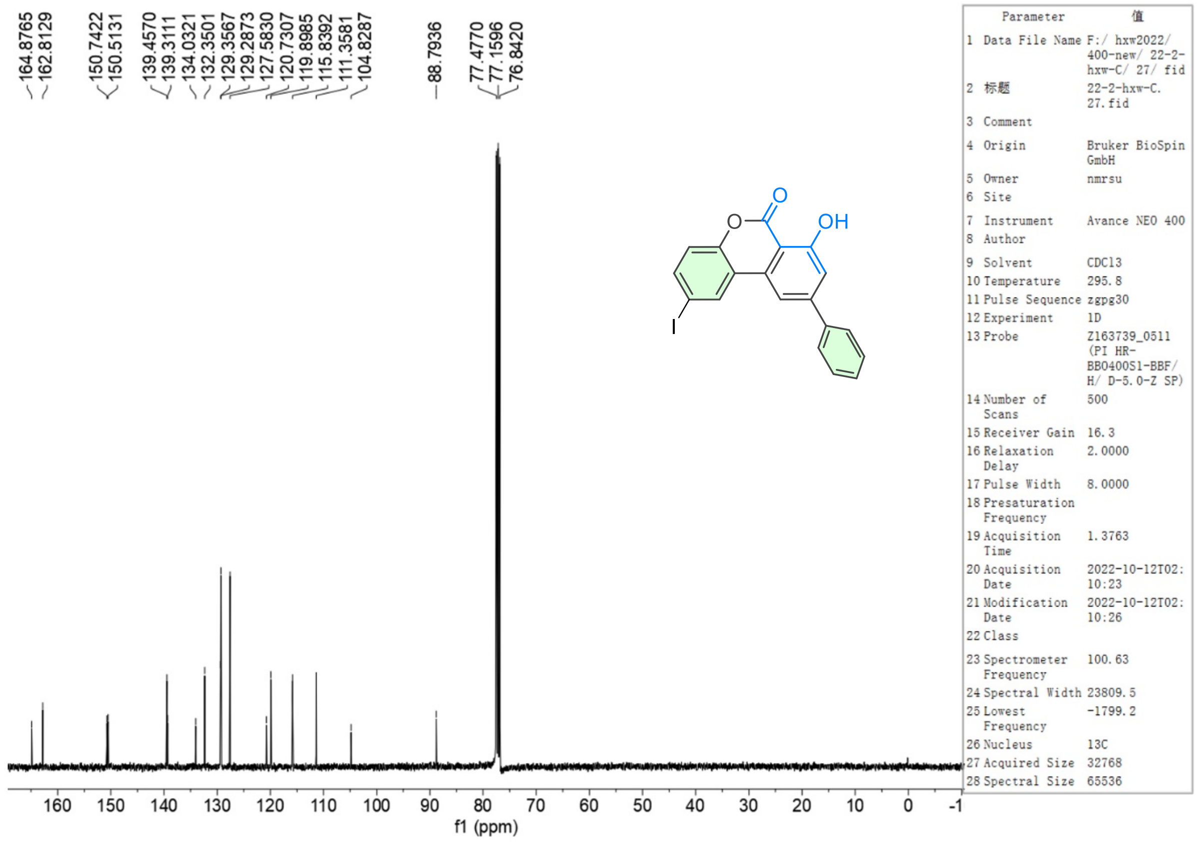

7-Hydroxy-2-methyl-9-(*p*-tolyl)-6*H*-benzo[*c*]chromen-6-one (product 3f)

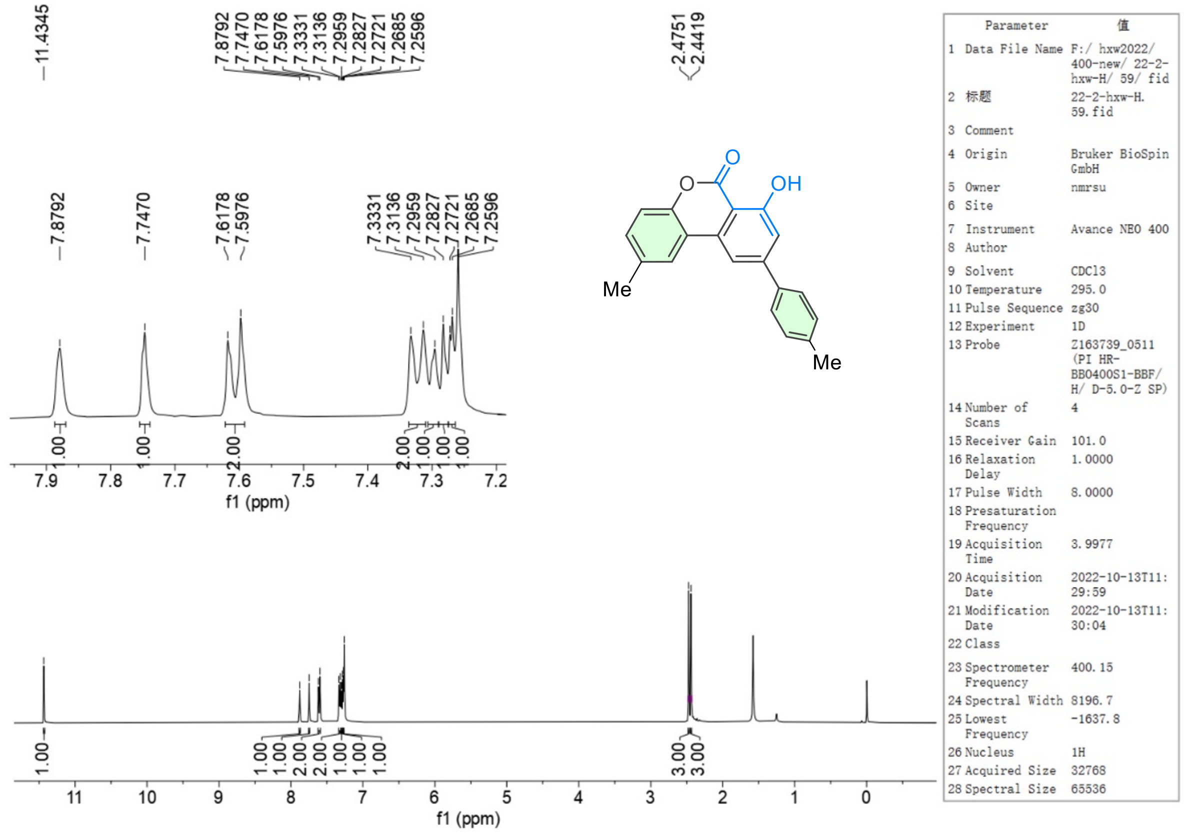

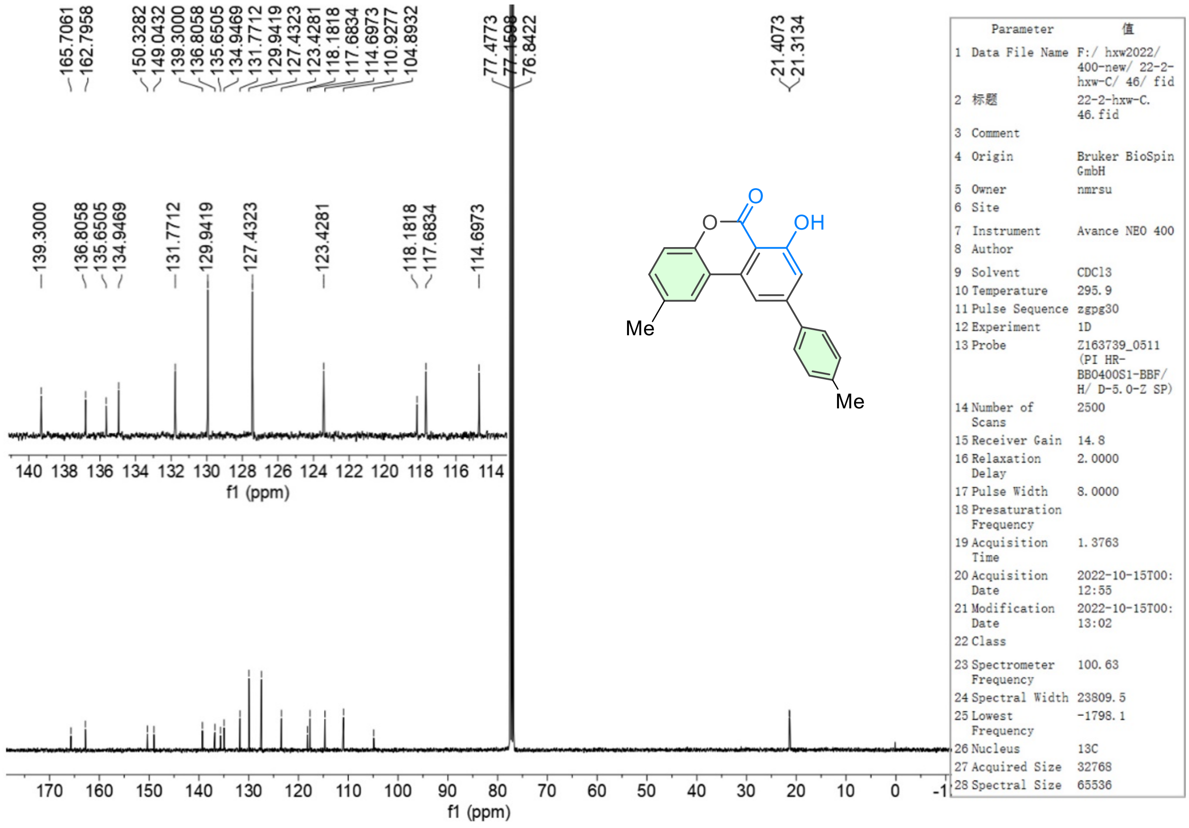

2-Chloro-7-hydroxy-9-(*p*-tolyl)-6*H*-benzo[*c*]chromen-6-one (product 3g)

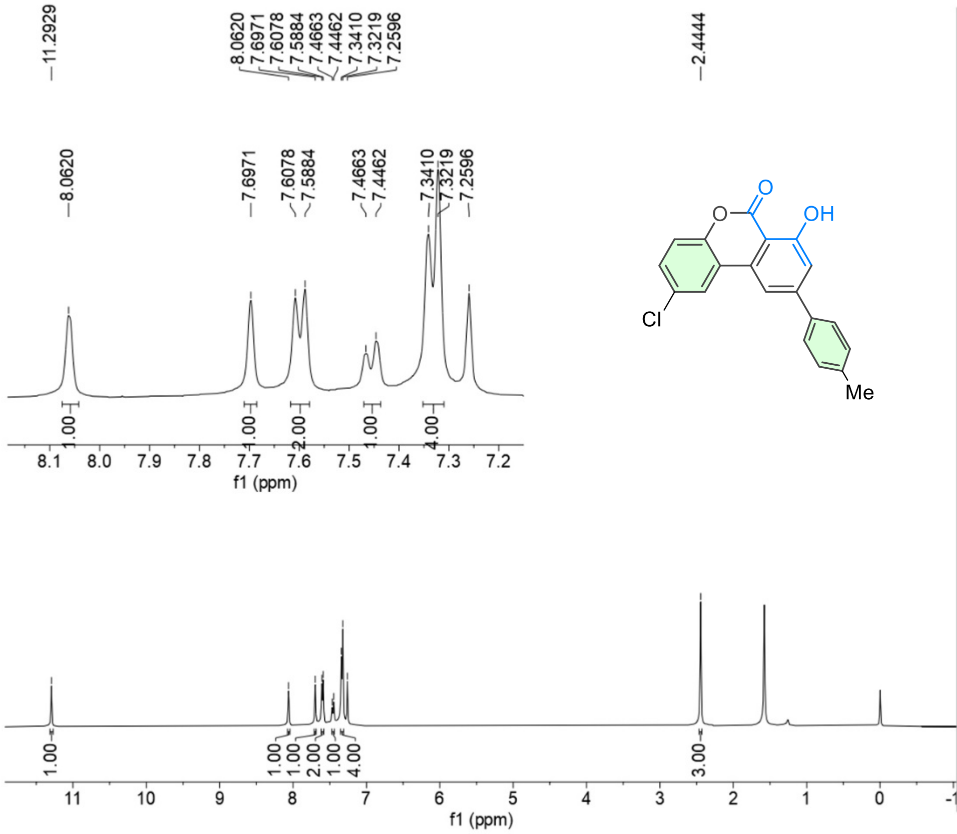

Parameter	值
1 Data File Name	F:/ hxw2022/ 400-new/ 22-2-hxw-H/ 148/ fid
2 标题	22-2-hxw-H. 148. fid
3 Comment	
4 Origin	Bruker BioSpin GmbH
5 Owner	nmrsu
6 Site	
7 Instrument	Avance NEO 400
8 Author	
9 Solvent	CDCl3
10 Temperature	295. 2
11 Pulse Sequence	zg30
12 Experiment	1D
13 Probe	Z163739_0511 (PI HR-BB0400S1-BBF/ H/ D-5. 0-Z SP)
14 Number of Scans	4
15 Receiver Gain	101. 0
16 Relaxation Delay	1. 0000
17 Pulse Width	8. 0000
18 Presaturation Frequency	
19 Acquisition Time	3. 9977
20 Acquisition Date	2022-11-09T21: 41:54
21 Modification Date	2022-11-09T21: 42:06
22 Class	
23 Spectrometer Frequency	400. 15
24 Spectral Width	8196. 7
25 Lowest Frequency	-1637. 0
26 Nucleus	1H
27 Acquired Size	32768
28 Spectral Size	65536

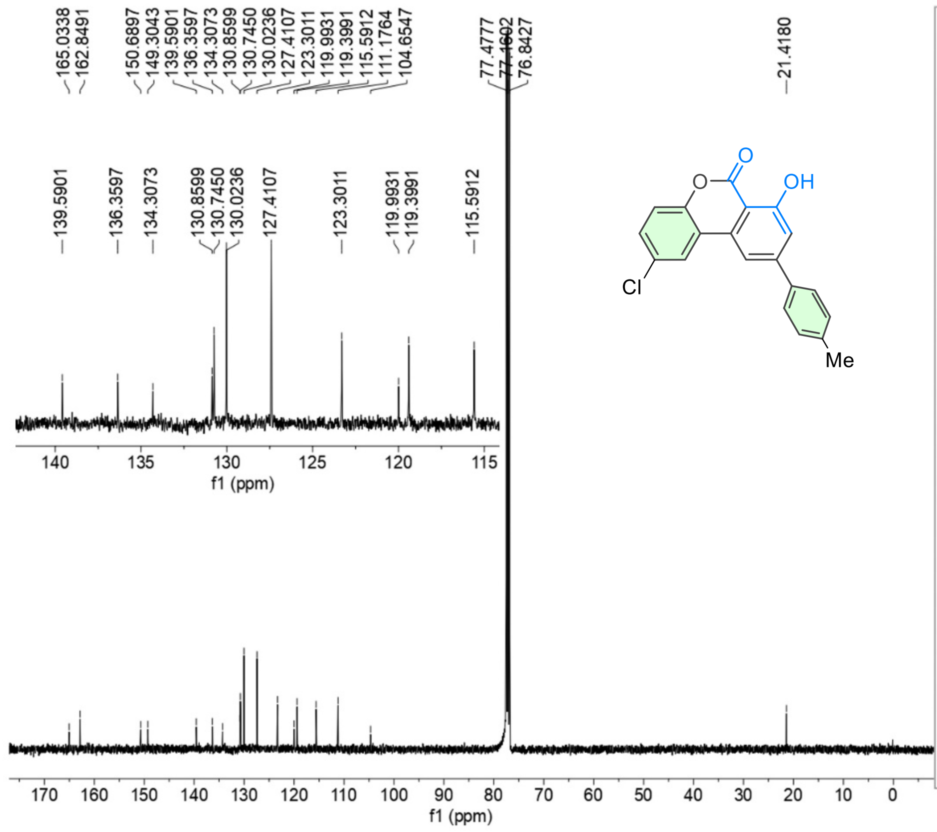

Parameter	值
1 Data File Name	F:/ hxw2022/ 400-new/ 22-2-hxw-C/ 98/ fid
2 标题	22-2-hxw-C. 98. fid
3 Comment	
4 Origin	Bruker BioSpin GmbH
5 Owner	nmrsu
6 Site	
7 Instrument	Avance NEO 400
8 Author	
9 Solvent	CDCl3
10 Temperature	296. 1
11 Pulse Sequence	zgpg30
12 Experiment	1D
13 Probe	Z163739_0511 (PI HR-BB0400S1-BBF/ H/ D-5. 0-Z SP)
14 Number of Scans	1024
15 Receiver Gain	16. 3
16 Relaxation Delay	2. 0000
17 Pulse Width	8. 0000
18 Presaturation Frequency	
19 Acquisition Time	1. 3763
20 Acquisition Date	2022-11-10T01: 29:21
21 Modification Date	2022-11-10T01: 29:36
22 Class	
23 Spectrometer Frequency	100. 63
24 Spectral Width	23809. 5
25 Lowest Frequency	-1798. 3
26 Nucleus	13C
27 Acquired Size	32768
28 Spectral Size	65536

7-Hydroxy-4-methoxy-9-(*p*-tolyl)-6*H*-benzo[*c*]chromen-6-one (product 3h)

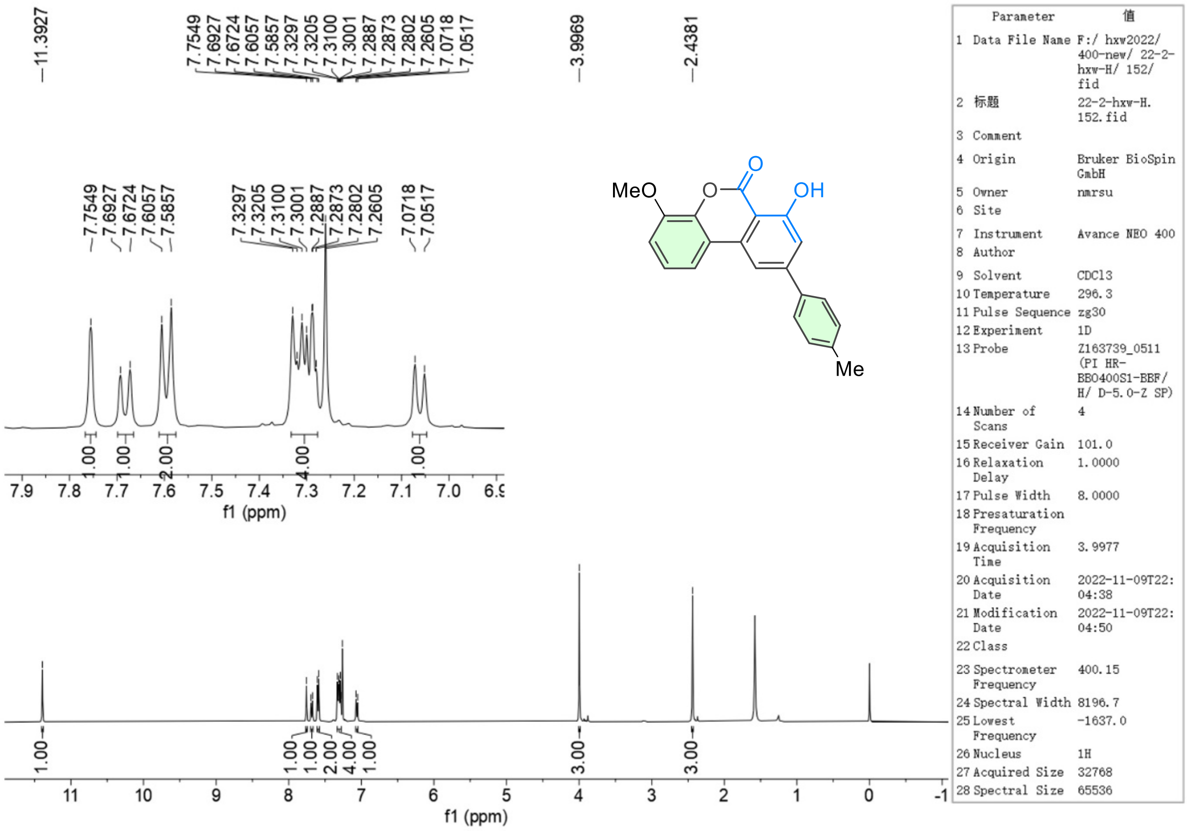

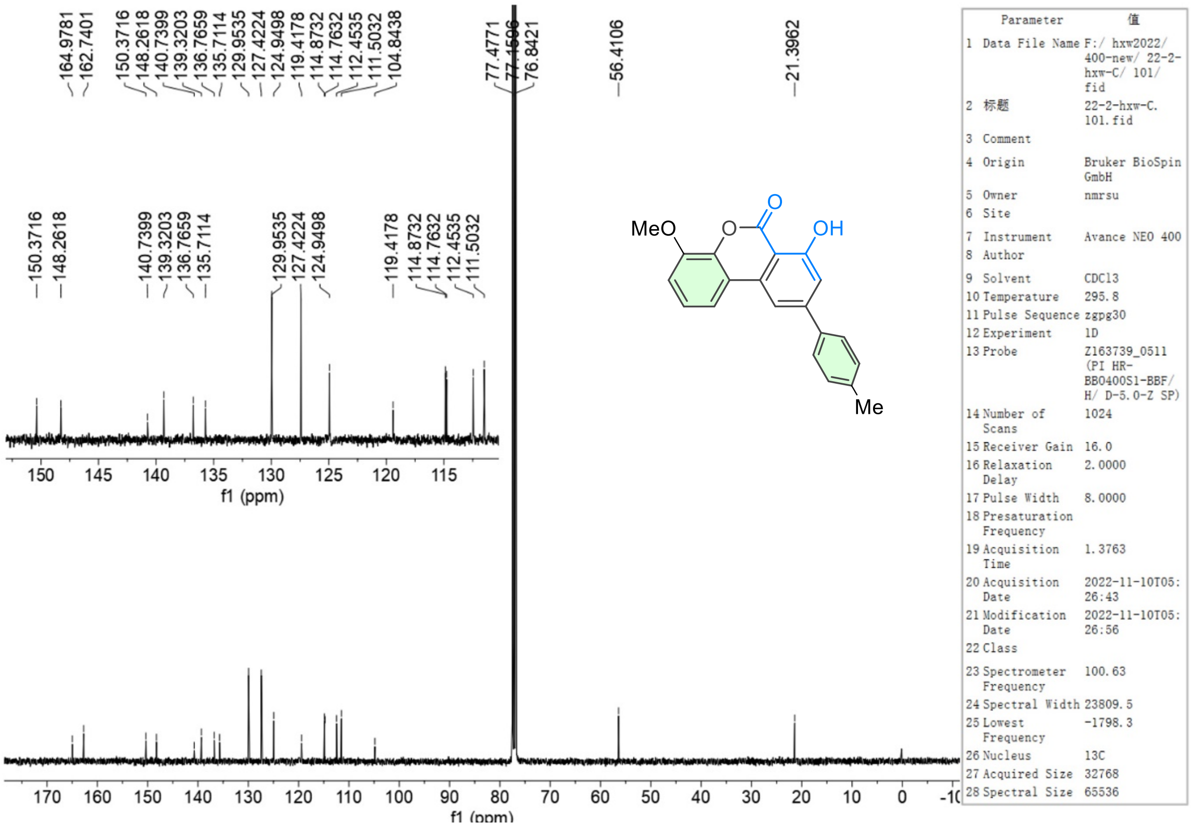

2,4-Dichloro-7-hydroxy-9-phenyl-6*H*-benzo[*c*]chromen-6-one (product 3i)

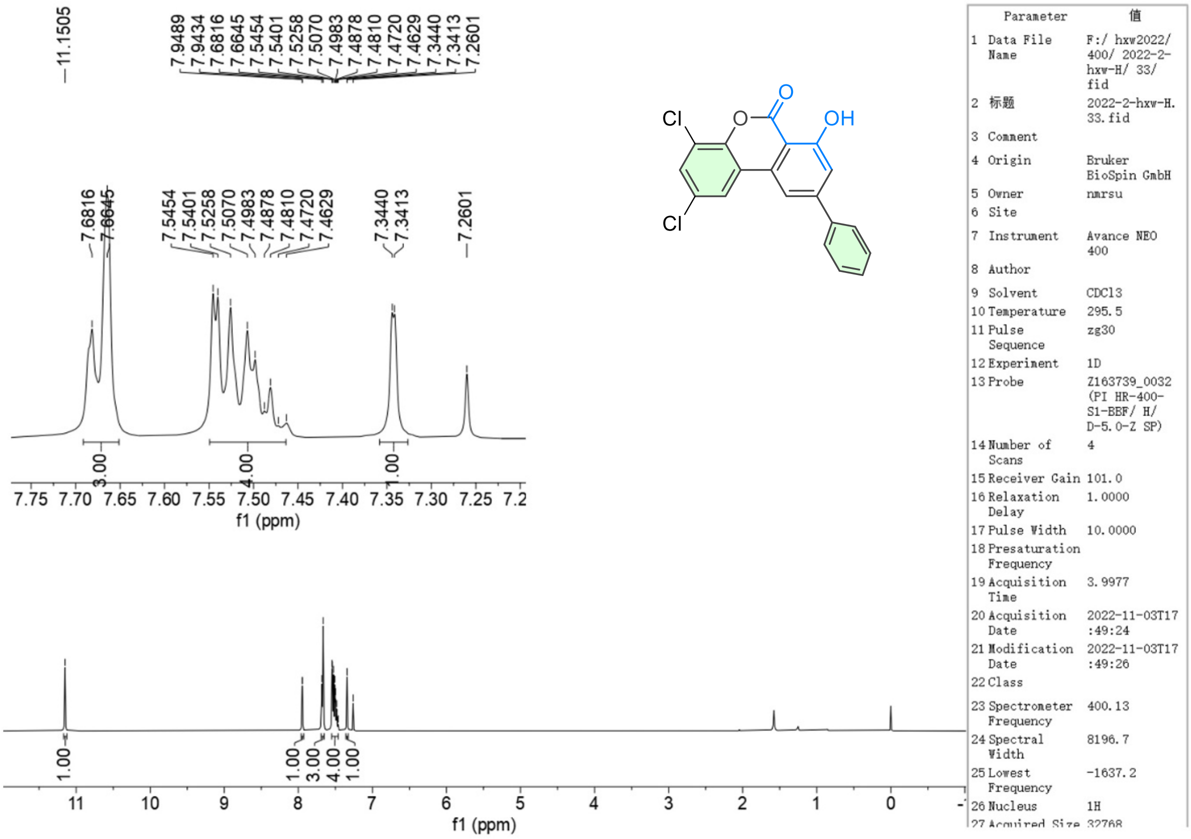

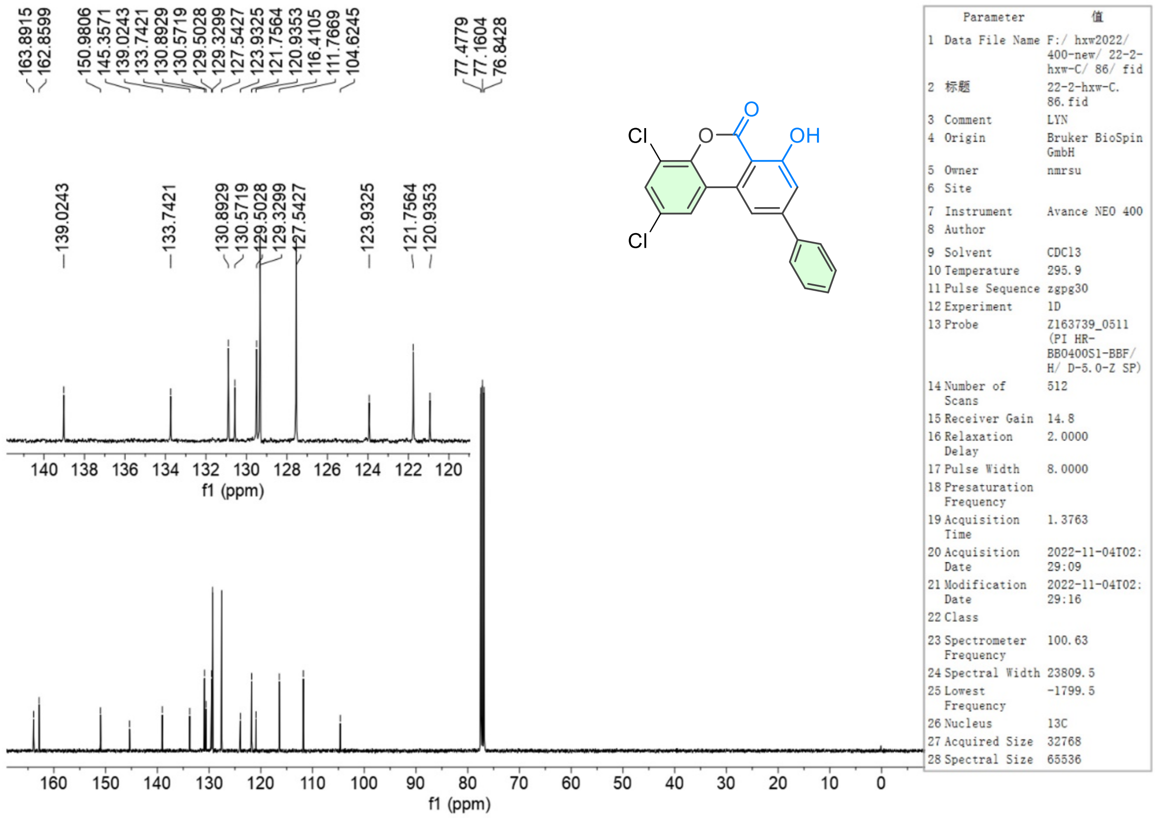

2,4-Dibromo-7-hydroxy-9-phenyl-6*H*-benzo[*c*]chromen-6-one (product 3j)

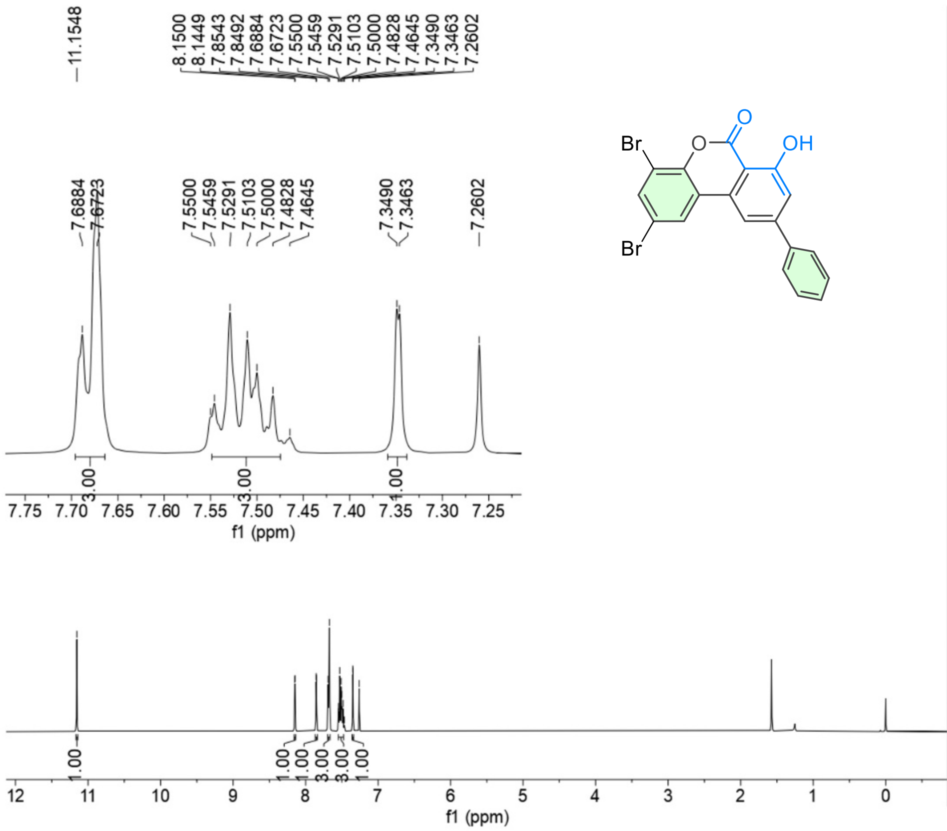

Parameter	值
1 Data File Name	F:/ hxw2022/ 400-new/ 22-2-hxw-H/ 122/ fid
2 标题	22-2-hxw-H. 122. fid
3 Comment	
4 Origin	Bruker BioSpin GmbH
5 Owner	nmrsu
6 Site	
7 Instrument	Avance NEO 400
8 Author	
9 Solvent	CDCl3
10 Temperature	295. 1
11 Pulse Sequence	zg30
12 Experiment	1D
13 Probe	Z163739_0511 (PI HR-BB0400S1-BBF/ H/ D-5. 0-Z SP)
14 Number of Scans	4
15 Receiver Gain	101. 0
16 Relaxation Delay	1. 0000
17 Pulse Width	8. 0000
18 Presaturation Frequency	
19 Acquisition Time	3. 9977
20 Acquisition Date	2022-11-01T15: 15:32
21 Modification Date	2022-11-01T15: 15:38
22 Class	
23 Spectrometer Frequency	400. 15
24 Spectral Width	8196. 7
25 Lowest Frequency	-1637. 0
26 Nucleus	1H
27 Acquired Size	32768
28 Spectral Size	65536

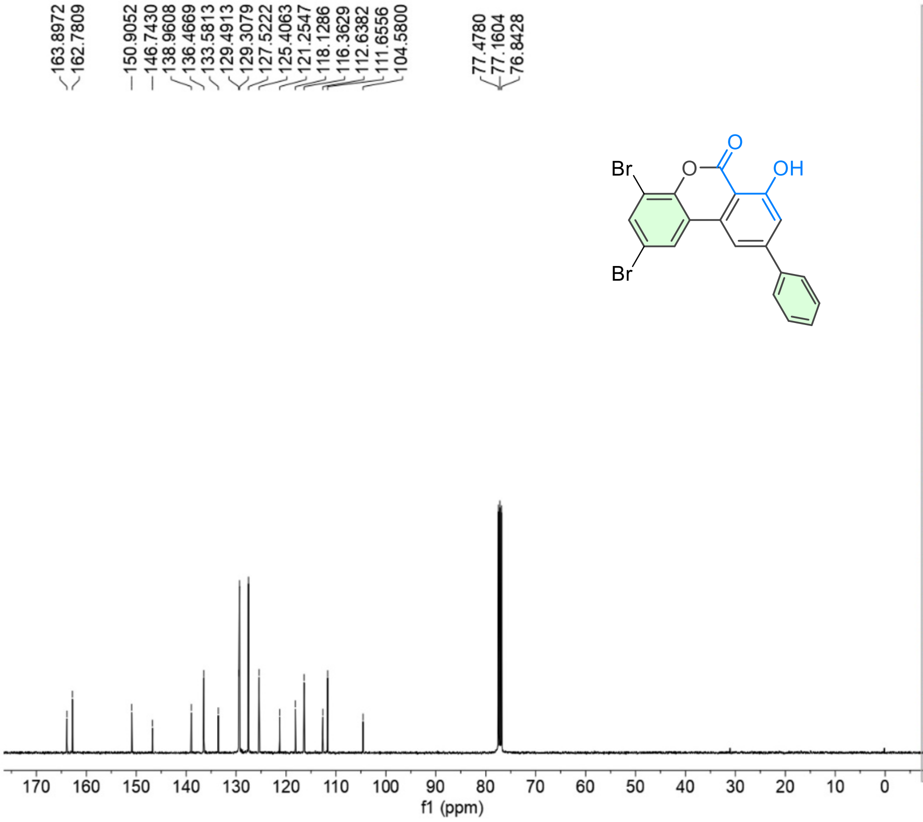

Parameter	值
1 Data File Name	F:/ hxw2022/ 400-new/ 22-2-hxw-C/ 78/ fid
2 标题	22-2-hxw-C. 78. fid
3 Comment	
4 Origin	Bruker BioSpin GmbH
5 Owner	nmrsu
6 Site	
7 Instrument	Avance NEO 400
8 Author	
9 Solvent	CDCl3
10 Temperature	295. 8
11 Pulse Sequence	zgpg30
12 Experiment	1D
13 Probe	Z163739_0511 (PI HR-BB0400S1-BBF/ H/ D-5. 0-Z SP)
14 Number of Scans	1024
15 Receiver Gain	16. 0
16 Relaxation Delay	2. 0000
17 Pulse Width	8. 0000
18 Presaturation Frequency	
19 Acquisition Time	1. 3763
20 Acquisition Date	2022-10-28T02: 45:01
21 Modification Date	2022-10-28T02: 45:04
22 Class	
23 Spectrometer Frequency	100. 63
24 Spectral Width	23809. 5
25 Lowest Frequency	-1800. 8
26 Nucleus	13C
27 Acquired Size	32768
28 Spectral Size	65536

3-Chloro-7-hydroxy-9-phenyl-6*H*-benzo[*c*]chromen-6-one (product 3k)

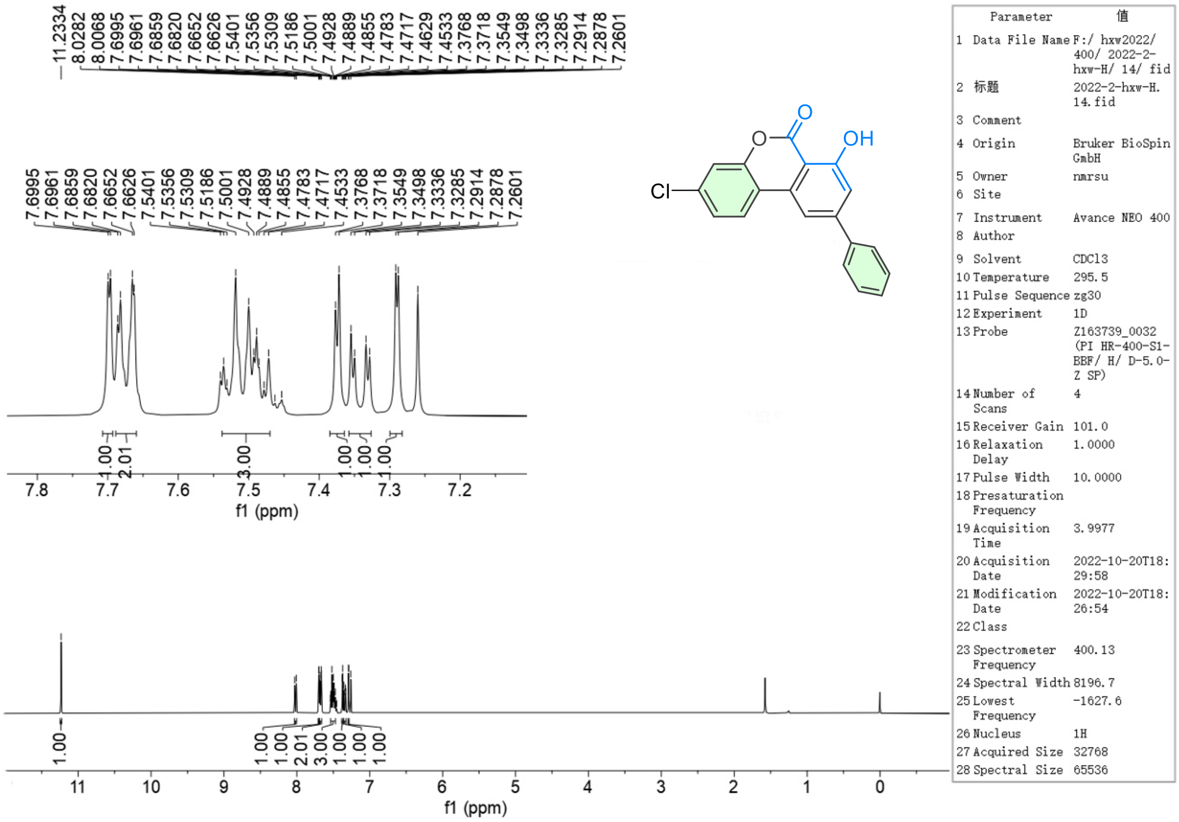

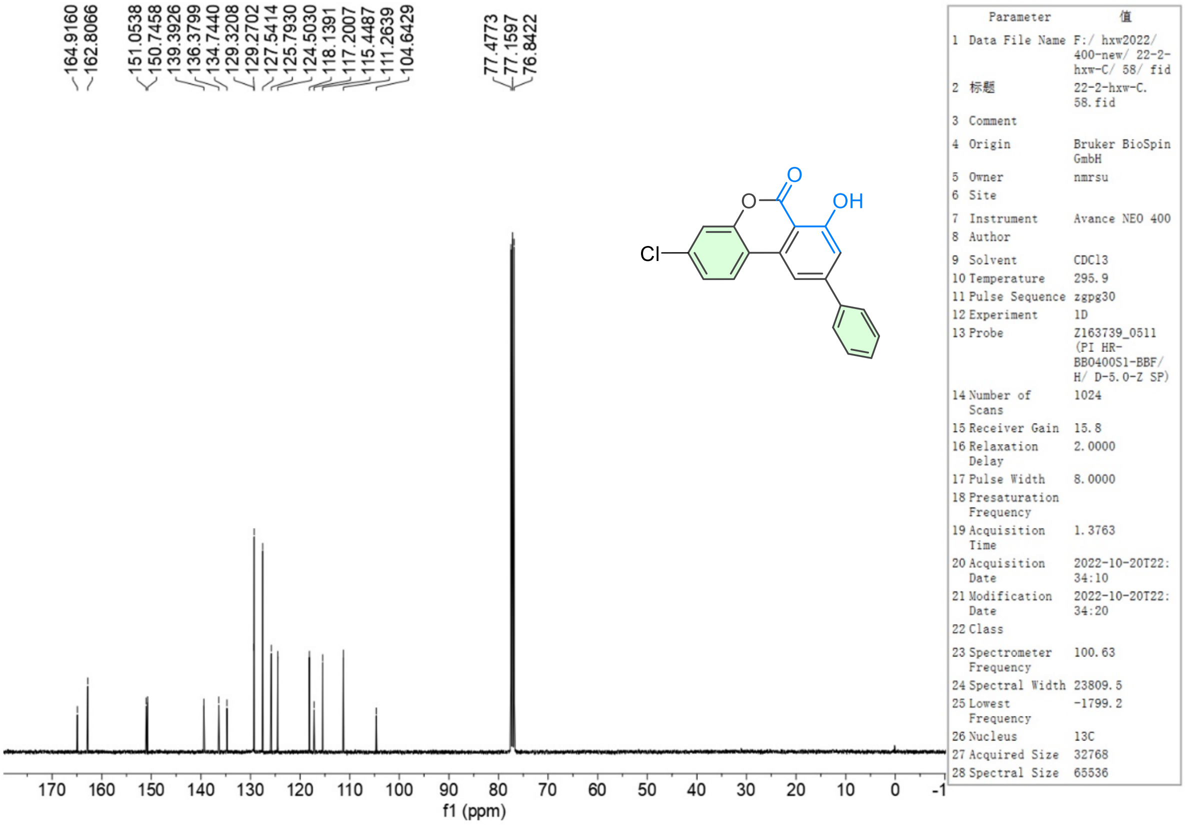

2-Chloro-7-hydroxy-9-(4-methoxyphenyl)-6*H*-benzo[*c*]chromen-6-one (product 3l)

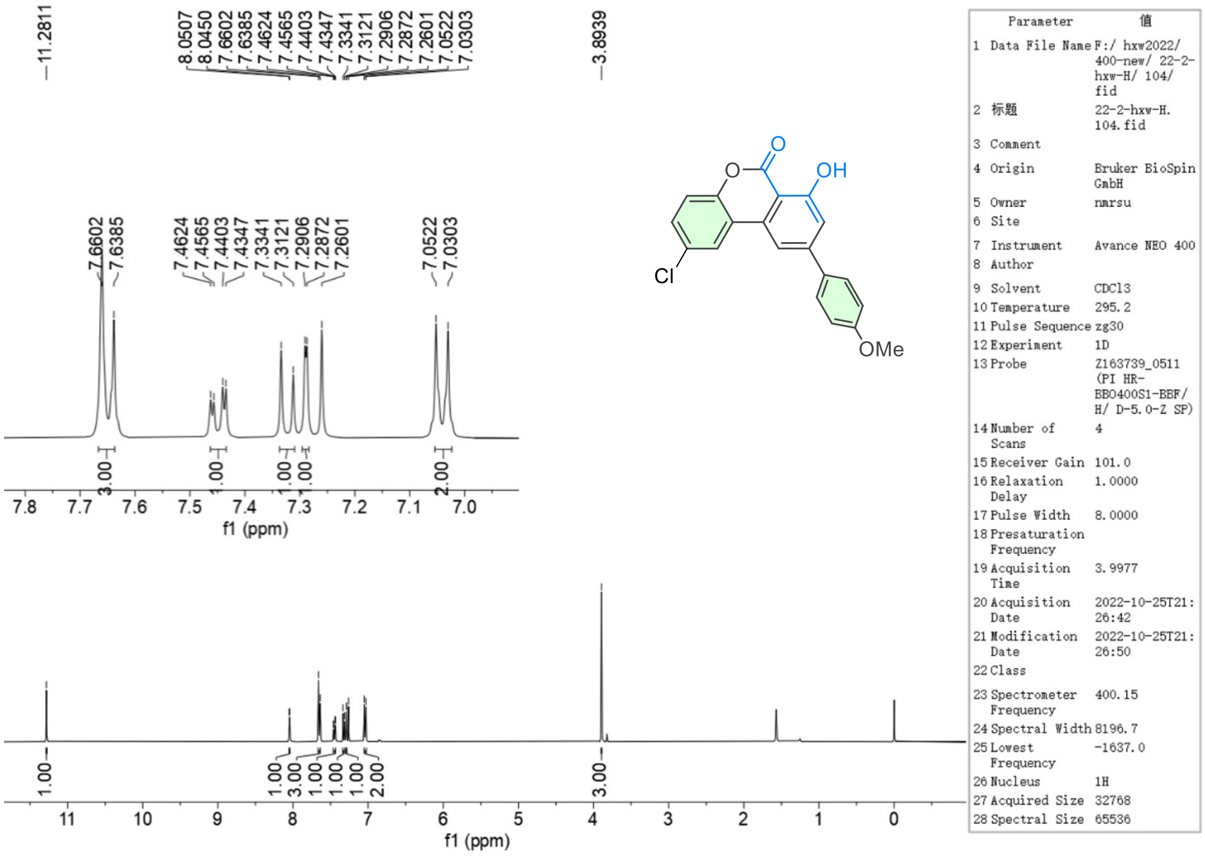

	Parameter	值
1	Data File Name	F:/ hxw2022/ 400-new/ 22-2-hxw-H/ 104/ fid
2	标题	22-2-hxw-H. 104. fid
3	Comment	
4	Origin	Bruker BioSpin GmbH
5	Owner	nmrsu
6	Site	
7	Instrument	Avance NEO 400
8	Author	
9	Solvent	CDCl3
10	Temperature	295. 2
11	Pulse Sequence	zg30
12	Experiment	1D
13	Probe	Z163739_0511 (PI HR-BB0400S1-BBF/ H/ D-5. 0-Z SP)
14	Number of Scans	4
15	Receiver Gain	101. 0
16	Relaxation Delay	1. 0000
17	Pulse Width	8. 0000
18	Presaturation Frequency	
19	Acquisition Time	3. 9977
20	Acquisition Date	2022-10-25T21: 26:42
21	Modification Date	2022-10-25T21: 26:50
22	Class	
23	Spectrometer Frequency	400. 15
24	Spectral Width	8196. 7
25	Lowest Frequency	-1637. 0
26	Nucleus	1H
27	Acquired Size	32768
28	Spectral Size	65536

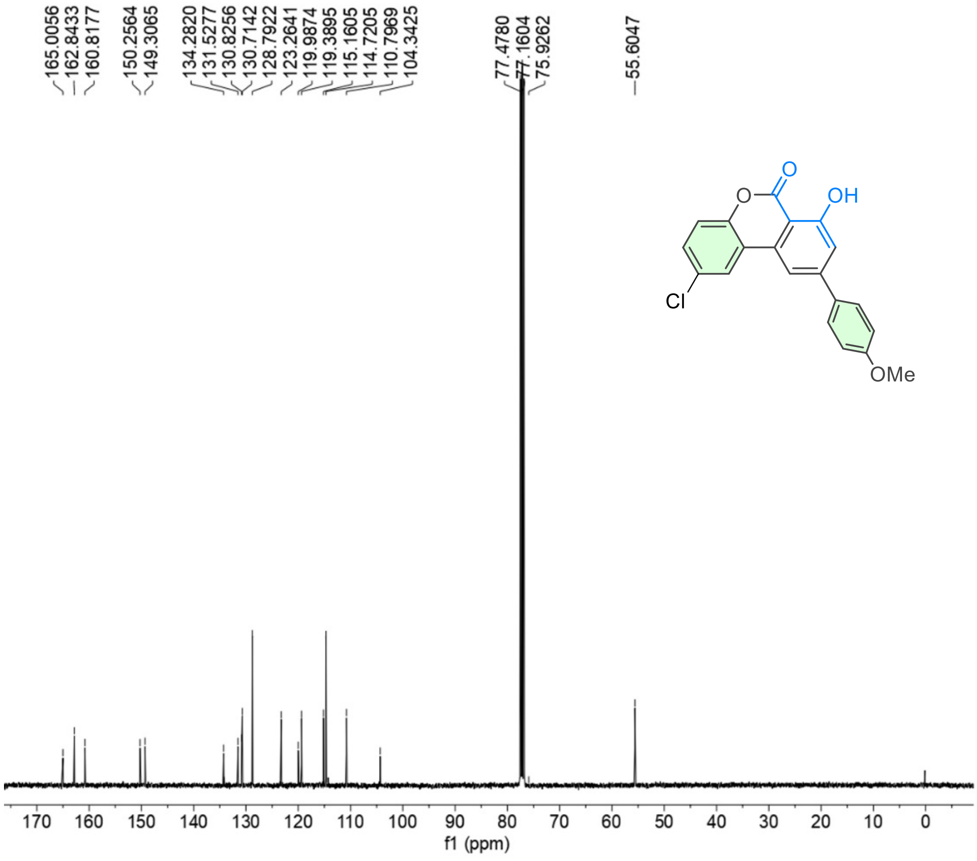

	Parameter	值
1	Data File Name	F:/ hxw2022/ 400-new/ 22-2-hxw-C/ 74/ fid
2	标题	22-2-hxw-C. 74. fid
3	Comment	
4	Origin	Bruker BioSpin GmbH
5	Owner	nmrsu
6	Site	
7	Instrument	Avance NEO 400
8	Author	
9	Solvent	CDCl3
10	Temperature	295. 9
11	Pulse Sequence	zgpg30
12	Experiment	1D
13	Probe	Z163739_0511 (PI HR-BB0400S1-BBF/ H/ D-5. 0-Z SP)
14	Number of Scans	1024
15	Receiver Gain	14. 8
16	Relaxation Delay	2. 0000
17	Pulse Width	8. 0000
18	Presaturation Frequency	
19	Acquisition Time	1. 3763
20	Acquisition Date	2022-10-26T0 0:51:23
21	Modification Date	2022-10-26T0 0:51:32
22	Class	
23	Spectrometer Frequency	100. 63
24	Spectral Width	23809. 5
25	Lowest Frequency	-1798. 5
26	Nucleus	13C

9-(4-Fluorophenyl)-7-hydroxy-6*H*-benzo[*c*]chromen-6-one (product 3m)

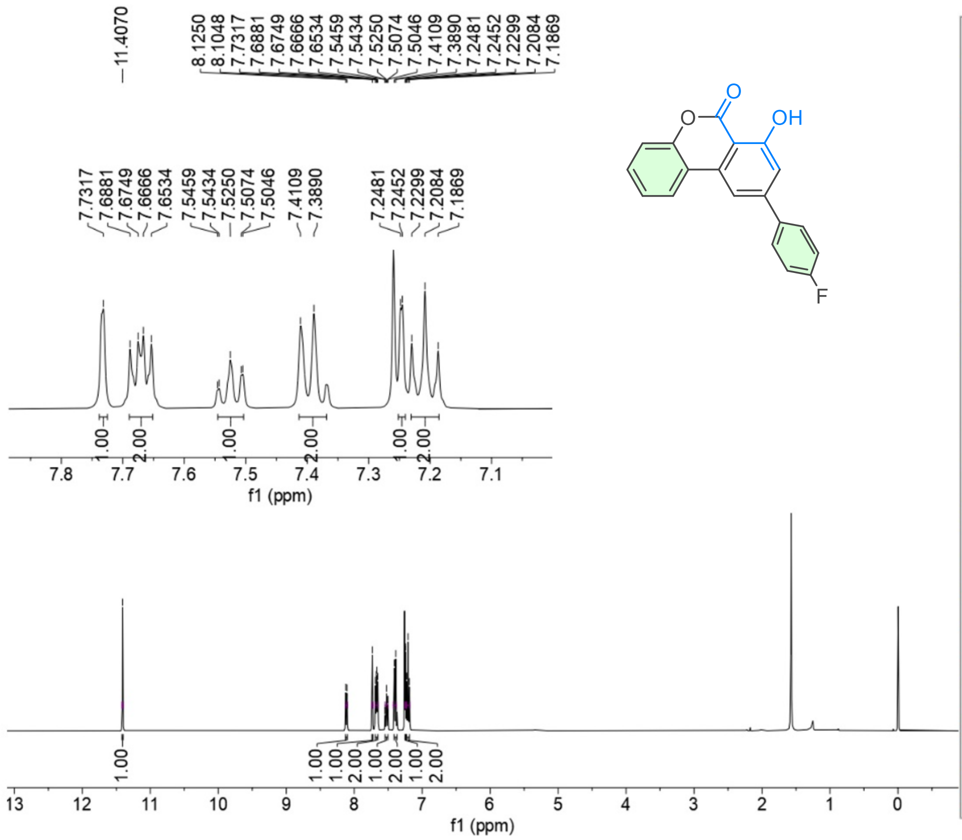

Parameter	值
1 Data File Name	F:/ hxw2022/ 400-new/ 22-2-hxw-H/ 105/ fid
2 标题	22-2-hxw-H. 105. fid
3 Comment	1
4 Origin	Bruker BioSpin GmbH
5 Owner	nmrsu
6 Site	
7 Instrument	Avance NEO 400
8 Author	
9 Solvent	CDCl3
10 Temperature	295. 2
11 Pulse Sequence	zg30
12 Experiment	1D
13 Probe	Z163739_0511 (PI HR-BB0400S1-BBF/ H/ D-5. 0-Z SP)
14 Number of Scans	4
15 Receiver Gain	101. 0
16 Relaxation Delay	1. 0000
17 Pulse Width	8. 0000
18 Presaturation Frequency	
19 Acquisition Time	3. 9977
20 Acquisition Date	2022-10-27T21:49:25
21 Modification Date	2022-10-27T21:49:28
22 Class	
23 Spectrometer Frequency	400. 15
24 Spectral Width	8196. 7
25 Lowest Frequency	-1637. 4
26 Nucleus	1H
27 Acquired Size	32768
28 Spectral Size	65536

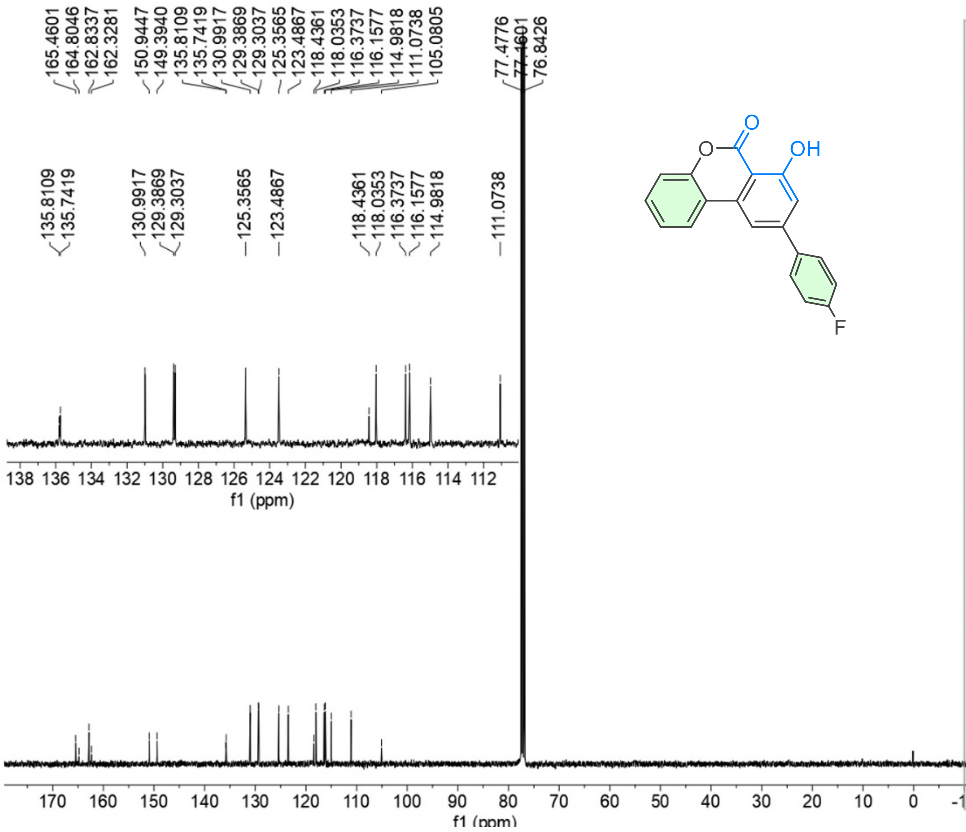

Parameter	值
1 Data File Name	F:/ hxw2022/ 400-new/ 22-2-hxw-C/ 77/ fid
2 标题	22-2-hxw-C. 77. fid
3 Comment	
4 Origin	Bruker BioSpin GmbH
5 Owner	nmrsu
6 Site	
7 Instrument	Avance NEO 400
8 Author	
9 Solvent	CDCl3
10 Temperature	295. 9
11 Pulse Sequence	zgpg30
12 Experiment	1D
13 Probe	Z163739_0511 (PI HR-BB0400S1-BBF/ H/ D-5. 0-Z SP)
14 Number of Scans	1024
15 Receiver Gain	15. 1
16 Relaxation Delay	2. 0000
17 Pulse Width	8. 0000
18 Presaturation Frequency	
19 Acquisition Time	1. 3763
20 Acquisition Date	2022-10-28T01:38:54
21 Modification Date	2022-10-28T01:39:00
22 Class	
23 Spectrometer Frequency	100. 63
24 Spectral Width	23809. 5
25 Lowest Frequency	-1798. 4
26 Nucleus	13C
27 Acquired Size	32768
28 Spectral Size	65536

9-(4-Fluorophenyl)-7-hydroxy-6*H*-benzo[*c*]chromen-6-one (product 3m)

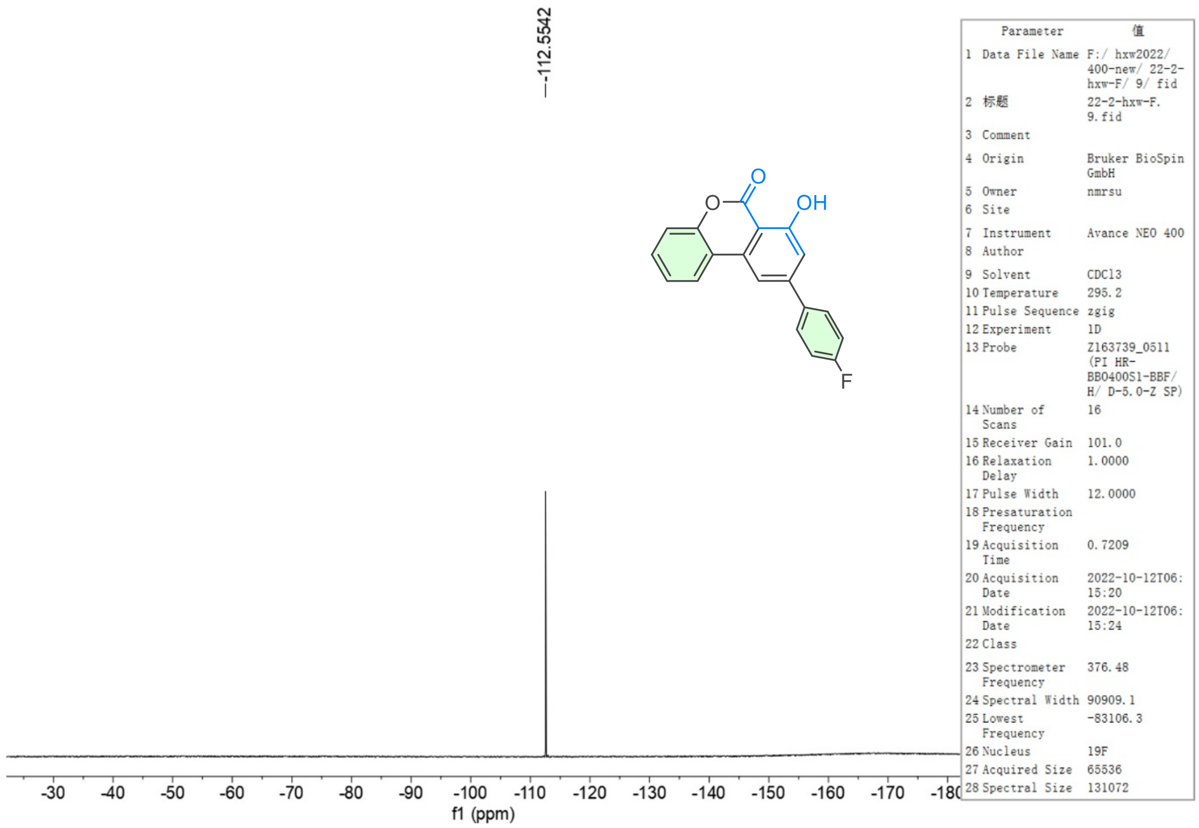

Parameter	值
1 Data File Name	F:/ hxw2022/ 400-new/ 22-2-hxw-F/ 9/ fid
2 标题	22-2-hxw-F. 9. fid
3 Comment	
4 Origin	Bruker BioSpin GmbH
5 Owner	nmrsu
6 Site	
7 Instrument	Avance NEO 400
8 Author	
9 Solvent	CDCl3
10 Temperature	295. 2
11 Pulse Sequence	zgig
12 Experiment	1D
13 Probe	Z163739_0511 (PI HR-BB0400S1-BBF/ H/ D-5. 0-Z SP)
14 Number of Scans	16
15 Receiver Gain	101. 0
16 Relaxation Delay	1. 0000
17 Pulse Width	12. 0000
18 Presaturation Frequency	
19 Acquisition Time	0. 7209
20 Acquisition Date	2022-10-12T06: 15:20
21 Modification Date	2022-10-12T06: 15:24
22 Class	
23 Spectrometer Frequency	376. 48
24 Spectral Width	90909. 1
25 Lowest Frequency	-83106. 3
26 Nucleus	19F
27 Acquired Size	65536
28 Spectral Size	131072

9-(4-Chlorophenyl)-7-hydroxy-6*H*-benzo[*c*]chromen-6-one (product 3n)

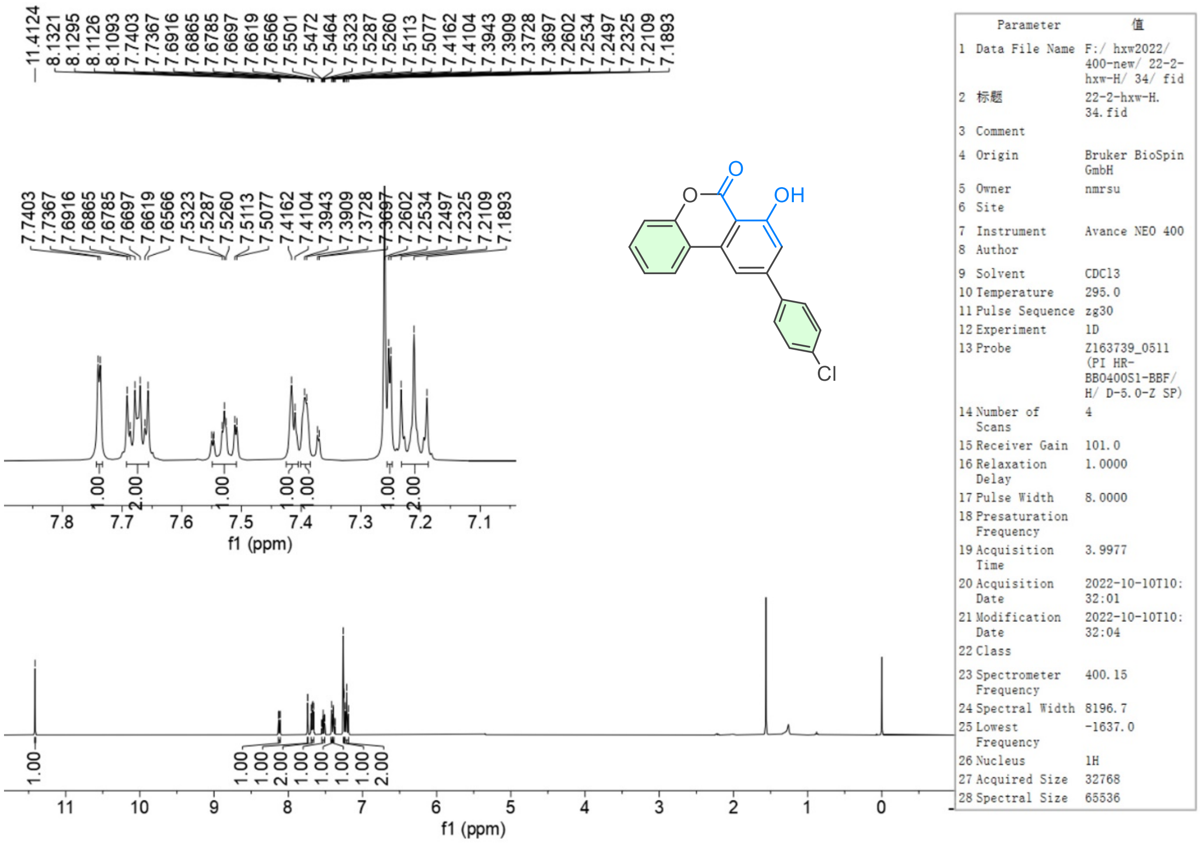

Parameter	值
1 Data File Name	F:/ hxw2022/ 400-new/ 22-2-hxw-H/ 34/ fid
2 标题	22-2-hxw-H. 34. fid
3 Comment	
4 Origin	Bruker BioSpin GmbH
5 Owner	nmrsu
6 Site	
7 Instrument	Avance NEO 400
8 Author	
9 Solvent	CDCl3
10 Temperature	295. 0
11 Pulse Sequence	zg30
12 Experiment	1D
13 Probe	Z163739_0511 (PI HR-BB0400S1-BBF/ H/ D-5. 0-Z SP)
14 Number of Scans	4
15 Receiver Gain	101. 0
16 Relaxation Delay	1. 0000
17 Pulse Width	8. 0000
18 Presaturation Frequency	
19 Acquisition Time	3. 9977
20 Acquisition Date	2022-10-10T10:32:01
21 Modification Date	2022-10-10T10:32:04
22 Class	
23 Spectrometer Frequency	400. 15
24 Spectral Width	8196. 7
25 Lowest Frequency	-1637. 0
26 Nucleus	1H
27 Acquired Size	32768
28 Spectral Size	65536

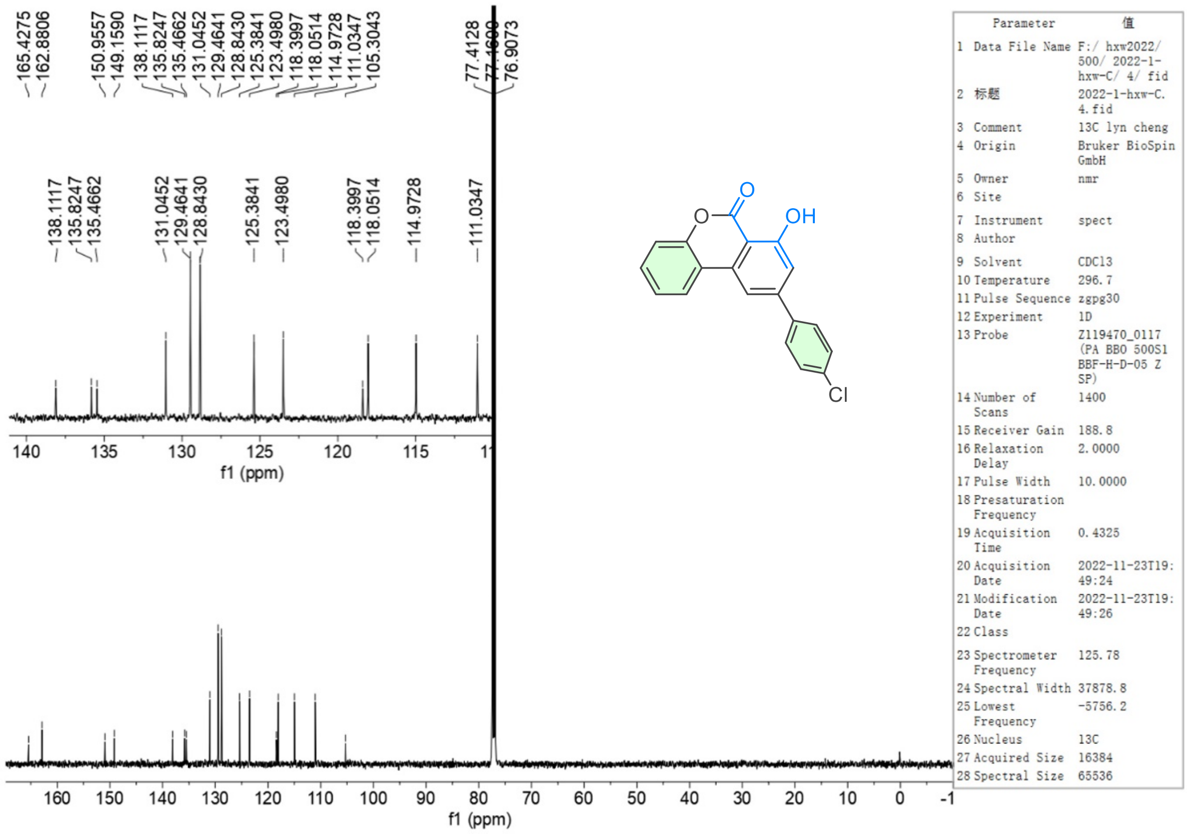

Parameter	值
1 Data File Name	F:/ hxw2022/ 500/ 2022-1-hxw-C/ 4/ fid
2 标题	2022-1-hxw-C. 4. fid
3 Comment	13C lyn cheng
4 Origin	Bruker BioSpin GmbH
5 Owner	nmr
6 Site	
7 Instrument	spect
8 Author	
9 Solvent	CDCl3
10 Temperature	296. 7
11 Pulse Sequence	zgpg30
12 Experiment	1D
13 Probe	Z119470_0117 (PA BBO 500S1 BBF-H-D-05 Z SP)
14 Number of Scans	1400
15 Receiver Gain	188. 8
16 Relaxation Delay	2. 0000
17 Pulse Width	10. 0000
18 Presaturation Frequency	
19 Acquisition Time	0. 4325
20 Acquisition Date	2022-11-23T19:49:24
21 Modification Date	2022-11-23T19:49:26
22 Class	
23 Spectrometer Frequency	125. 78
24 Spectral Width	37878. 8
25 Lowest Frequency	-5756. 2
26 Nucleus	13C
27 Acquired Size	16384
28 Spectral Size	65536

9-(4-Bromophenyl)-7-hydroxy-6*H*-benzo[*c*]chromen-6-one (product 3o)

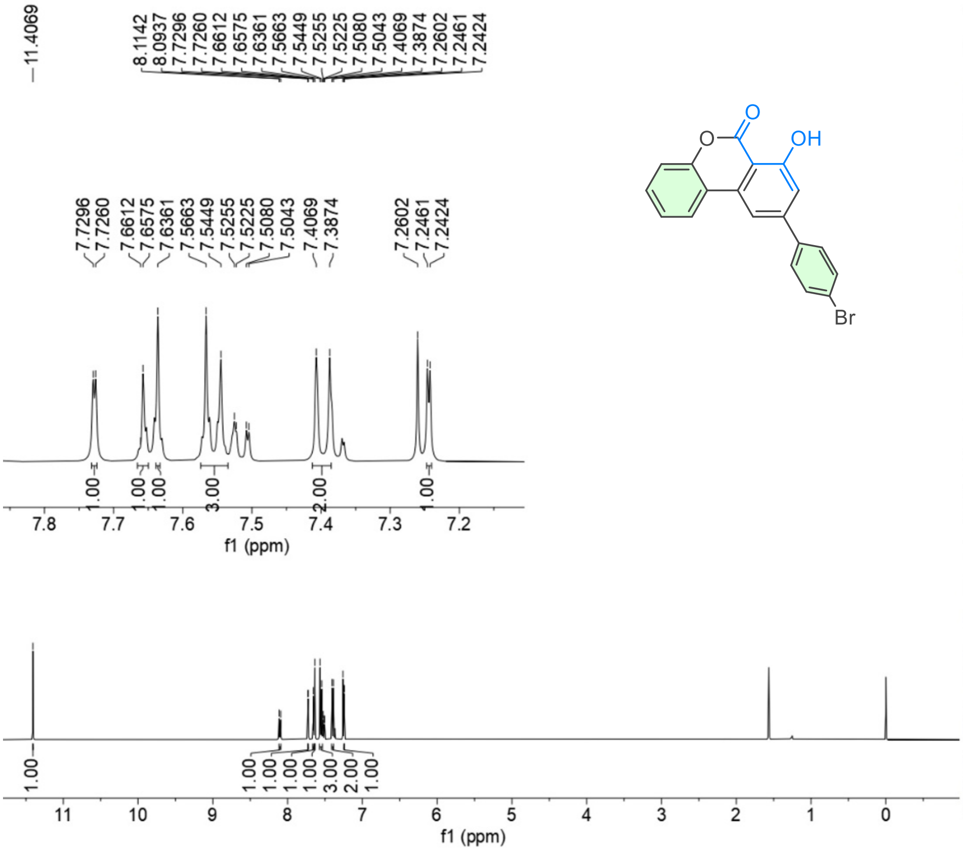

Parameter	值
1 Data File Name	F:/ hxw2022/ 400/ 2022-2-hxw-H/ 25/ fid
2 标题	2022-2-hxw-H. 25. fid
3 Comment	
4 Origin	Bruker BioSpin GmbH
5 Owner	nmrsu
6 Site	
7 Instrument	Avance NEO 400
8 Author	
9 Solvent	CDCl3
10 Temperature	295. 5
11 Pulse Sequence	zg30
12 Experiment	1D
13 Probe	Z163739_0032 (PI HR-400-S1-BBF/ H/ D-5. 0-Z SP)
14 Number of Scans	4
15 Receiver Gain	101. 0
16 Relaxation Delay	1. 0000
17 Pulse Width	10. 0000
18 Presaturation Frequency	
19 Acquisition Time	3. 9977
20 Acquisition Date	2022-10-25T20: 17:56
21 Modification Date	2022-10-25T20: 17:40
22 Class	
23 Spectrometer Frequency	400. 13
24 Spectral Width	8196. 7
25 Lowest Frequency	-1637. 2
26 Nucleus	1H
27 Acquired Size	32768
28 Spectral Size	65536

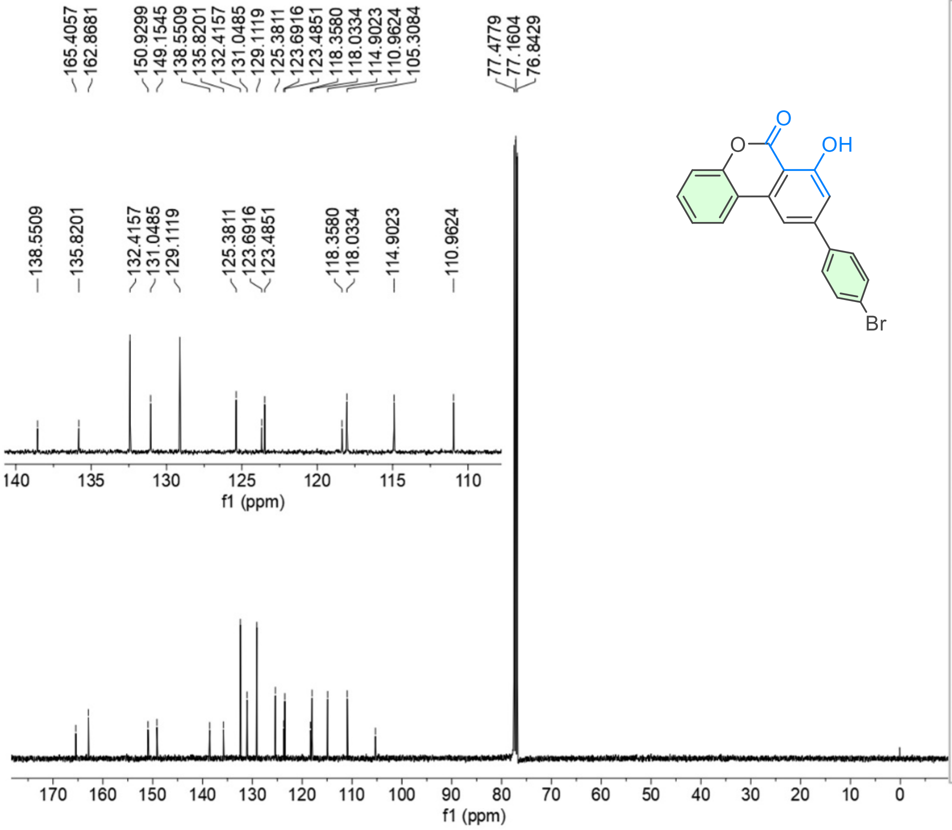

Parameter	值
1 Data File Name	F:/ hxw2022/ 400-new/ 22-2-hxw-C/ 70/ fid
2 标题	22-2-hxw-C. 70. fid
3 Comment	
4 Origin	Bruker BioSpin GmbH
5 Owner	nmrsu
6 Site	
7 Instrument	Avance NEO 400
8 Author	
9 Solvent	CDCl3
10 Temperature	295. 9
11 Pulse Sequence	zgpg30
12 Experiment	1D
13 Probe	Z163739_0511 (PI HR-BBO400S1-BBF/ H/ D-5. 0-Z SP)
14 Number of Scans	600
15 Receiver Gain	11. 8
16 Relaxation Delay	2. 0000
17 Pulse Width	8. 0000
18 Presaturation Frequency	
19 Acquisition Time	1. 3763
20 Acquisition Date	2022-10-26T06: 36:35
21 Modification Date	2022-10-26T06: 36:44
22 Class	
23 Spectrometer Frequency	100. 63
24 Spectral Width	23809. 5
25 Lowest Frequency	-1798. 6
26 Nucleus	13C
27 Acquired Size	32768
28 Spectral Size	65536

7-Hydroxy-9-(4-(trifluoromethyl)phenyl)-6*H*-benzo[*c*]chromen-6-one (product 3p)

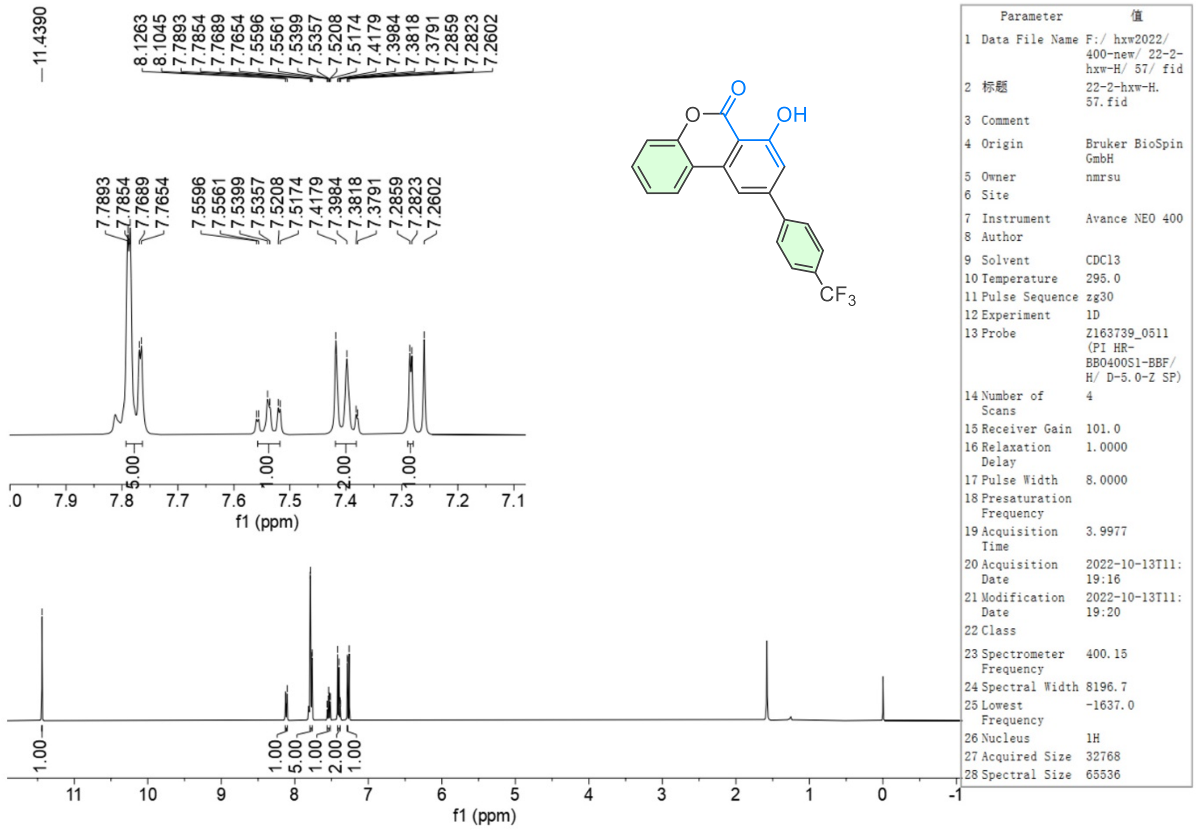

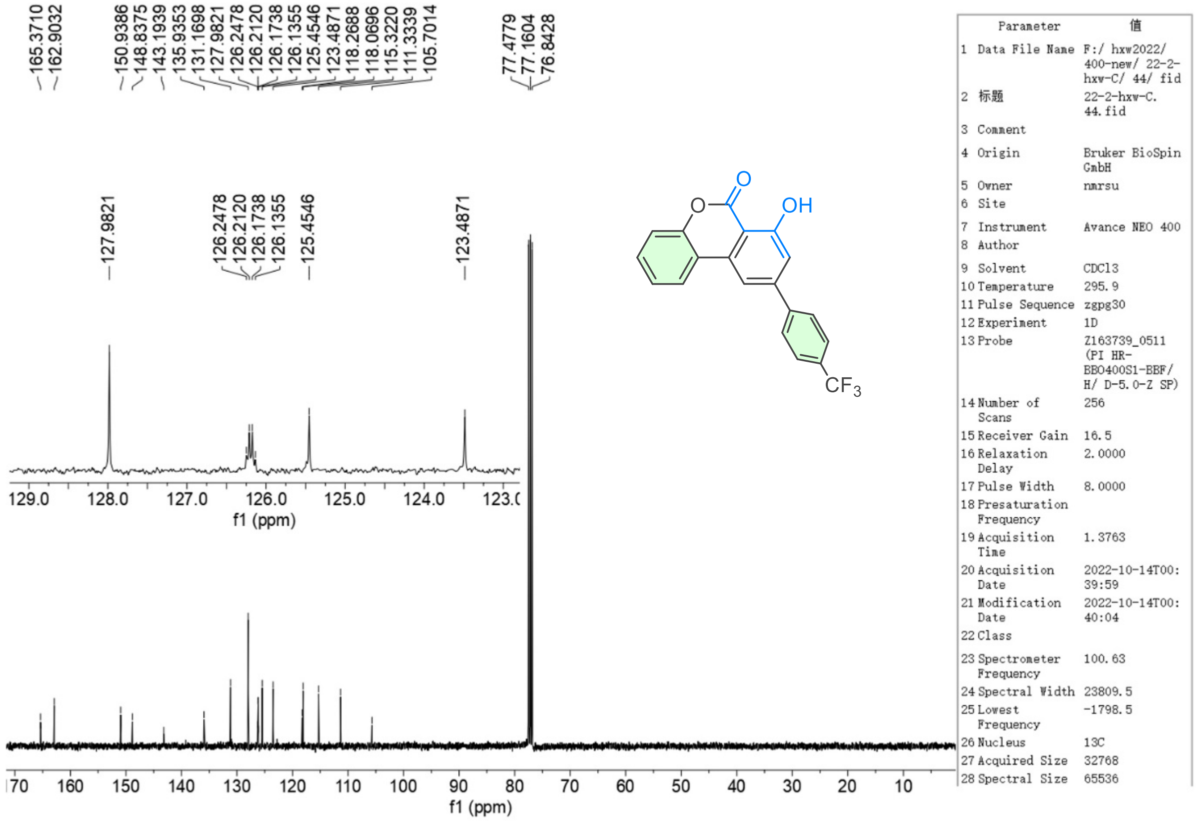

7-Hydroxy-9-(4-(trifluoromethyl)phenyl)-6*H*-benzo[*c*]chromen-6-one (product 3p)

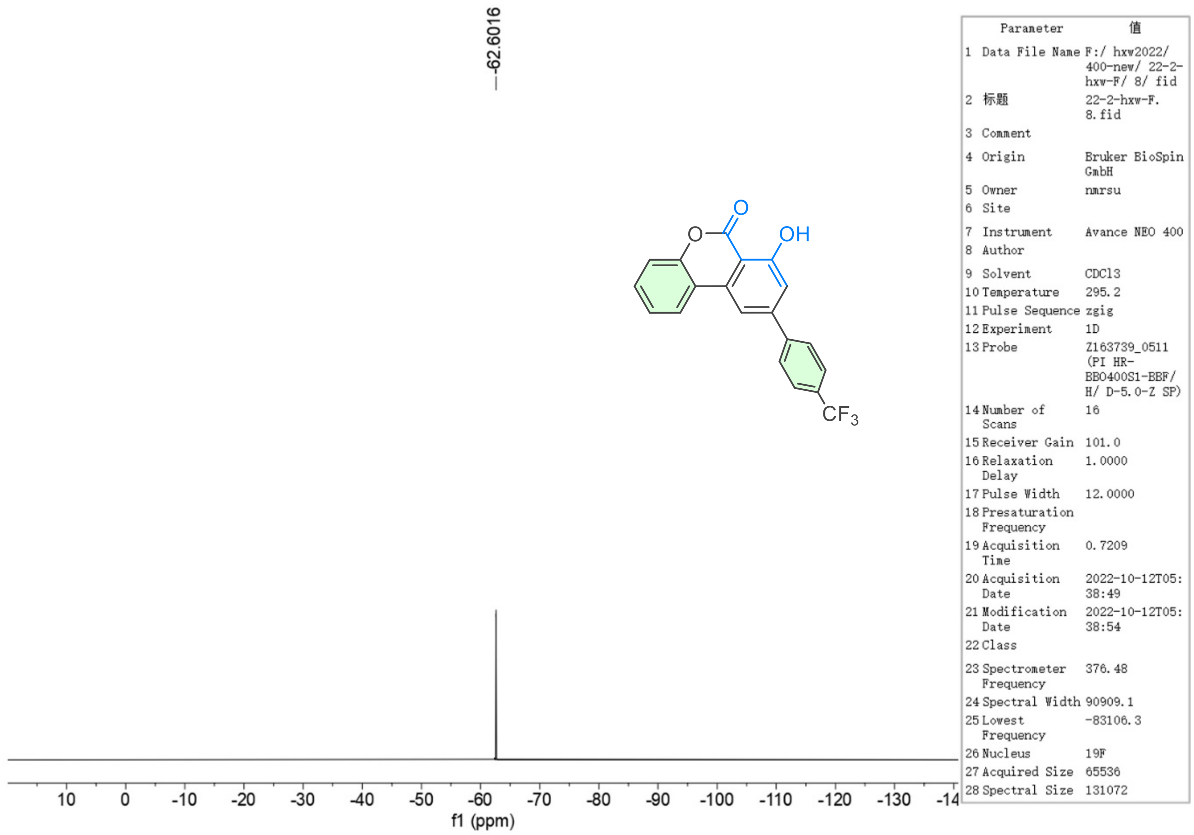

	Parameter	值
1	Data File Name	F:/ hxw2022/ 400-new/ 22-2-hxw-F/ 8/ fid
2	标题	22-2-hxw-F. 8. fid
3	Comment	
4	Origin	Bruker BioSpin GmbH
5	Owner	nmrsu
6	Site	
7	Instrument	Avance NEO 400
8	Author	
9	Solvent	CDCl3
10	Temperature	295. 2
11	Pulse Sequence	zgig
12	Experiment	1D
13	Probe	Z163739_0511 (PI HR-BBO400S1-BBF/ H/ D-5. 0-Z SP)
14	Number of Scans	16
15	Receiver Gain	101. 0
16	Relaxation Delay	1. 0000
17	Pulse Width	12. 0000
18	Presaturation Frequency	
19	Acquisition Time	0. 7209
20	Acquisition Date	2022-10-12T05: 38:49
21	Modification Date	2022-10-12T05: 38:54
22	Class	
23	Spectrometer Frequency	376. 48
24	Spectral Width	90909. 1
25	Lowest Frequency	-83106. 3
26	Nucleus	19F
27	Acquired Size	65536
28	Spectral Size	131072

2-Bromo-9-(4-bromophenyl)-7-hydroxy-6*H*-benzo[*c*]chromen-6-one (product 3q)

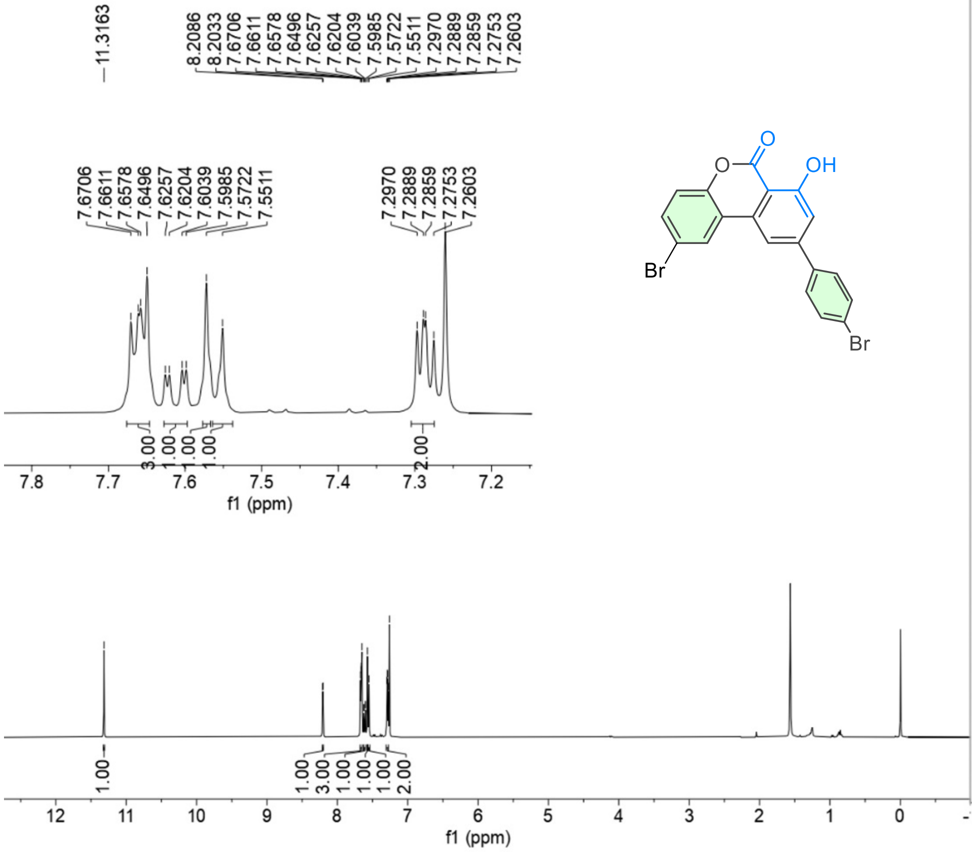

	Parameter	値
1	Data File Name	F:/ hxw2022/ 22-2hxw-H/ 170/ fid
2	标题	22-2-hxw-H. 170.fid
3	Comment	
4	Origin	Bruker BioSpin GmbH
5	Owner	nmrsu
6	Site	
7	Instrument	Avance NEO 400
8	Author	
9	Solvent	CDCl3
10	Temperature	295.0
11	Pulse Sequence	zg30
12	Experiment	1D
13	Probe	Z163739_0511 (PI HR-BB0400S1-BBF/ H/ D-5.0-Z SP)
14	Number of Scans	4
15	Receiver Gain	101.0
16	Relaxation Delay	1.0000
17	Pulse Width	8.0000
18	Presaturation Frequency	
19	Acquisition Time	3.9977
20	Acquisition Date	2022-11-15T21:26:40
21	Modification Date	2022-11-15T21:26:56
22	Class	
23	Spectrometer Frequency	400.15
24	Spectral Width	8196.7
25	Lowest Frequency	-1637.0
26	Nucleus	1H
27	Acquired Size	32768
28	Spectral Size	65536

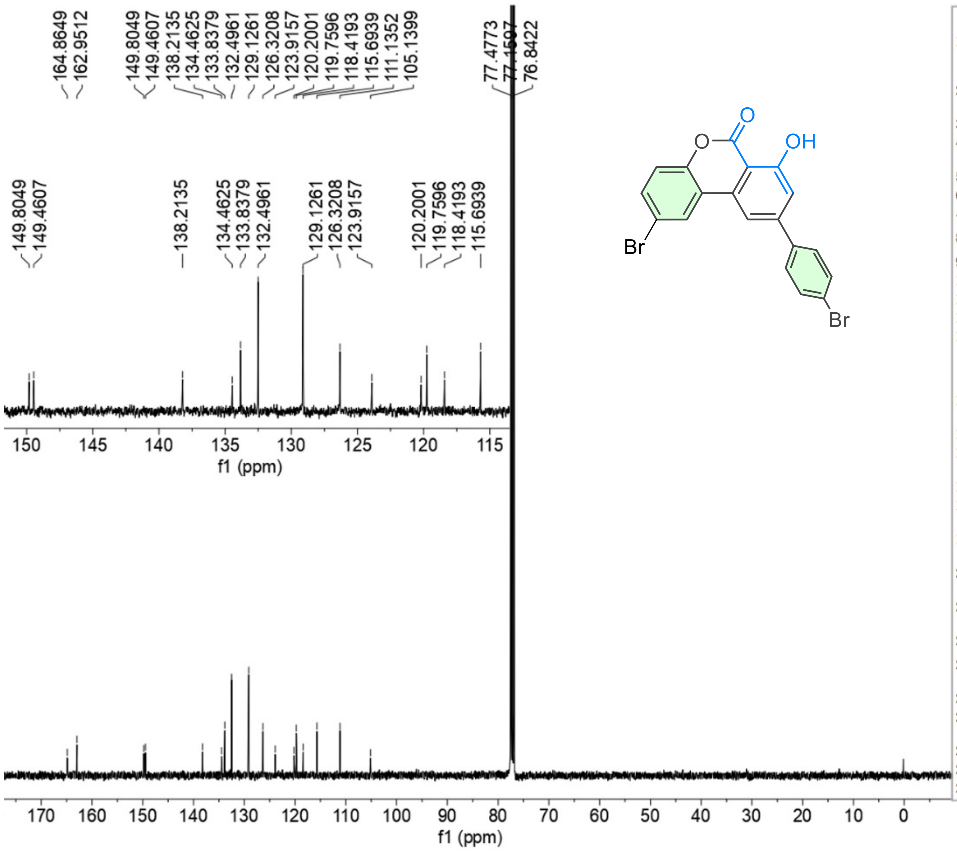

	Parameter	値
1	Data File Name	F:/ hxw2022/ 22-2HXWC/ 117/ fid
2	标题	22-2-hxw-C. 117.fid
3	Comment	lyn-bai
4	Origin	Bruker BioSpin GmbH
5	Owner	nmrsu
6	Site	
7	Instrument	Avance NEO 400
8	Author	
9	Solvent	CDCl3
10	Temperature	295.9
11	Pulse Sequence	zgpg30
12	Experiment	1D
13	Probe	Z163739_0511 (PI HR-BB0400S1-BBF/ H/ D-5.0-Z SP)
14	Number of Scans	1024
15	Receiver Gain	16.2
16	Relaxation Delay	2.0000
17	Pulse Width	8.0000
18	Presaturation Frequency	
19	Acquisition Time	1.3763
20	Acquisition Date	2022-11-16T06:15:23
21	Modification Date	2022-11-16T06:15:42
22	Class	
23	Spectrometer Frequency	100.63
24	Spectral Width	23809.5
25	Lowest Frequency	-1798.1
26	Nucleus	13C
27	Acquired Size	32768
28	Spectral Size	65536

2-Bromo-7-hydroxy-9-(*p*-tolyl)-6*H*-benzo[*c*]chromen-6-one (product 3r)

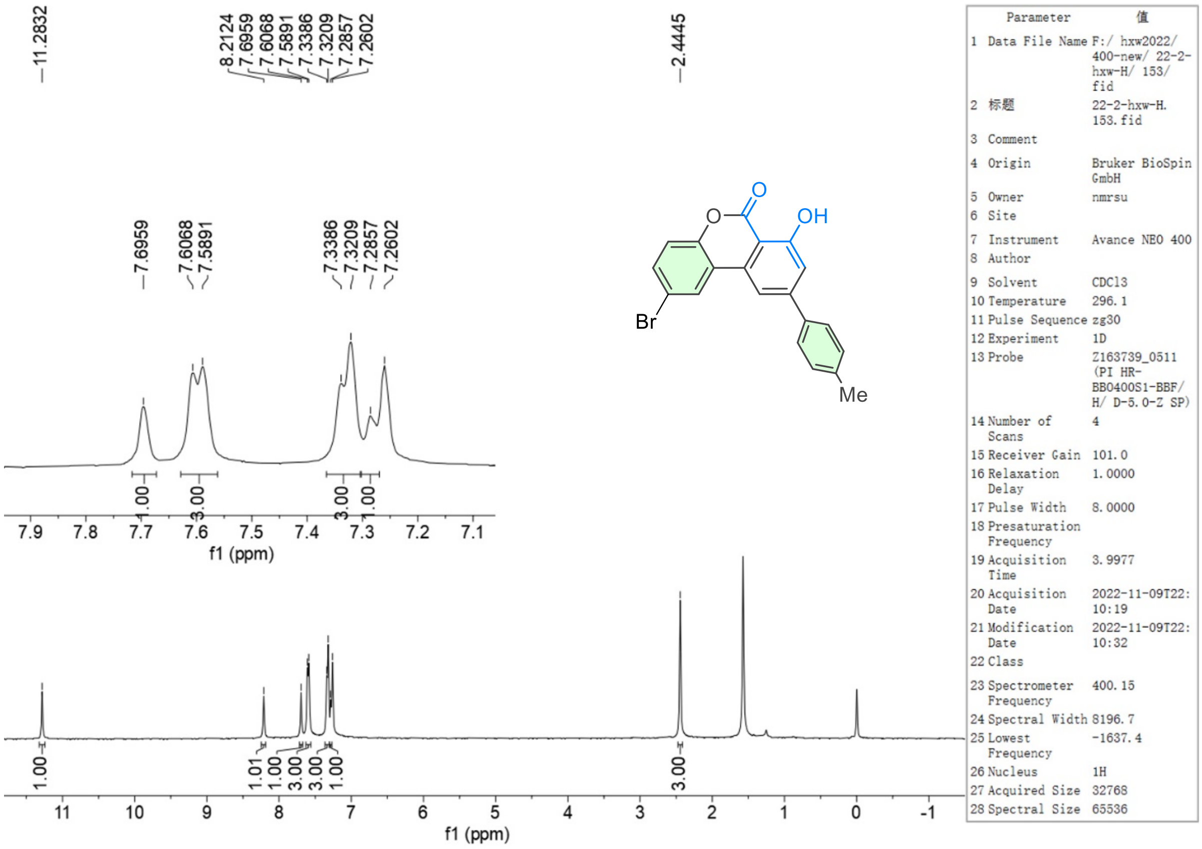

Parameter	值
1 Data File Name	F:/ hxw2022/ 400-new/ 22-2-hxw-H/ 153/ fid
2 标题	22-2-hxw-H. 153. fid
3 Comment	
4 Origin	Bruker BioSpin GmbH
5 Owner	nmrsu
6 Site	
7 Instrument	Avance NEO 400
8 Author	
9 Solvent	CDCl3
10 Temperature	296. 1
11 Pulse Sequence	zg30
12 Experiment	1D
13 Probe	Z163739_0511 (PI HR-BB0400S1-BBF/ H/ D-5. 0-Z SP)
14 Number of Scans	4
15 Receiver Gain	101. 0
16 Relaxation Delay	1. 0000
17 Pulse Width	8. 0000
18 Presaturation Frequency	
19 Acquisition Time	3. 9977
20 Acquisition Date	2022-11-09T22: 10:19
21 Modification Date	2022-11-09T22: 10:32
22 Class	
23 Spectrometer Frequency	400. 15
24 Spectral Width	8196. 7
25 Lowest Frequency	-1637. 4
26 Nucleus	1H
27 Acquired Size	32768
28 Spectral Size	65536

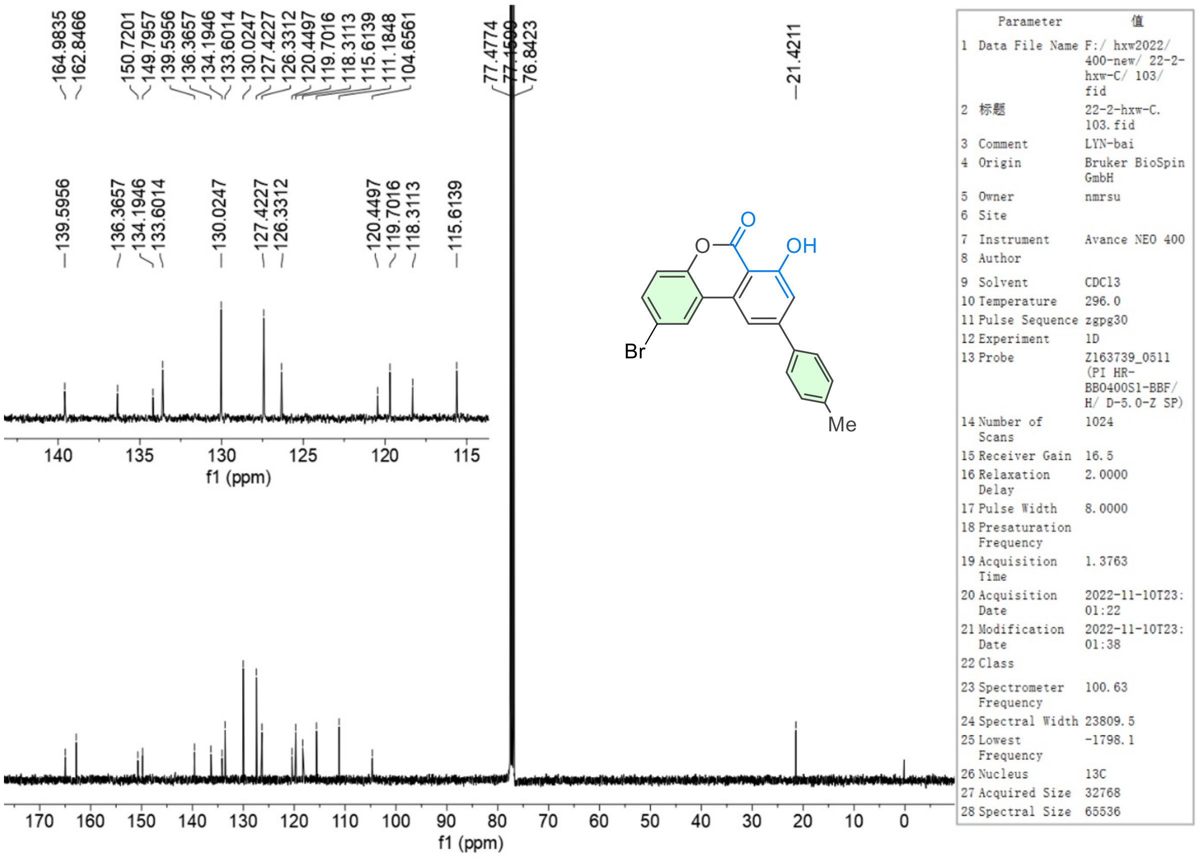

Parameter	值
1 Data File Name	F:/ hxw2022/ 400-new/ 22-2-hxw-C/ 103/ fid
2 标题	22-2-hxw-C. 103. fid
3 Comment	LYN-bai
4 Origin	Bruker BioSpin GmbH
5 Owner	nmrsu
6 Site	
7 Instrument	Avance NEO 400
8 Author	
9 Solvent	CDCl3
10 Temperature	296. 0
11 Pulse Sequence	zgpg30
12 Experiment	1D
13 Probe	Z163739_0511 (PI HR-BB0400S1-BBF/ H/ D-5. 0-Z SP)
14 Number of Scans	1024
15 Receiver Gain	16. 5
16 Relaxation Delay	2. 0000
17 Pulse Width	8. 0000
18 Presaturation Frequency	
19 Acquisition Time	1. 3763
20 Acquisition Date	2022-11-10T23: 01:22
21 Modification Date	2022-11-10T23: 01:38
22 Class	
23 Spectrometer Frequency	100. 63
24 Spectral Width	23809. 5
25 Lowest Frequency	-1798. 1
26 Nucleus	13C
27 Acquired Size	32768
28 Spectral Size	65536

2-Bromo-9-(4-ethylphenyl)-7-hydroxy-6*H*-benzo[*c*]chromen-6-one (product 3s)

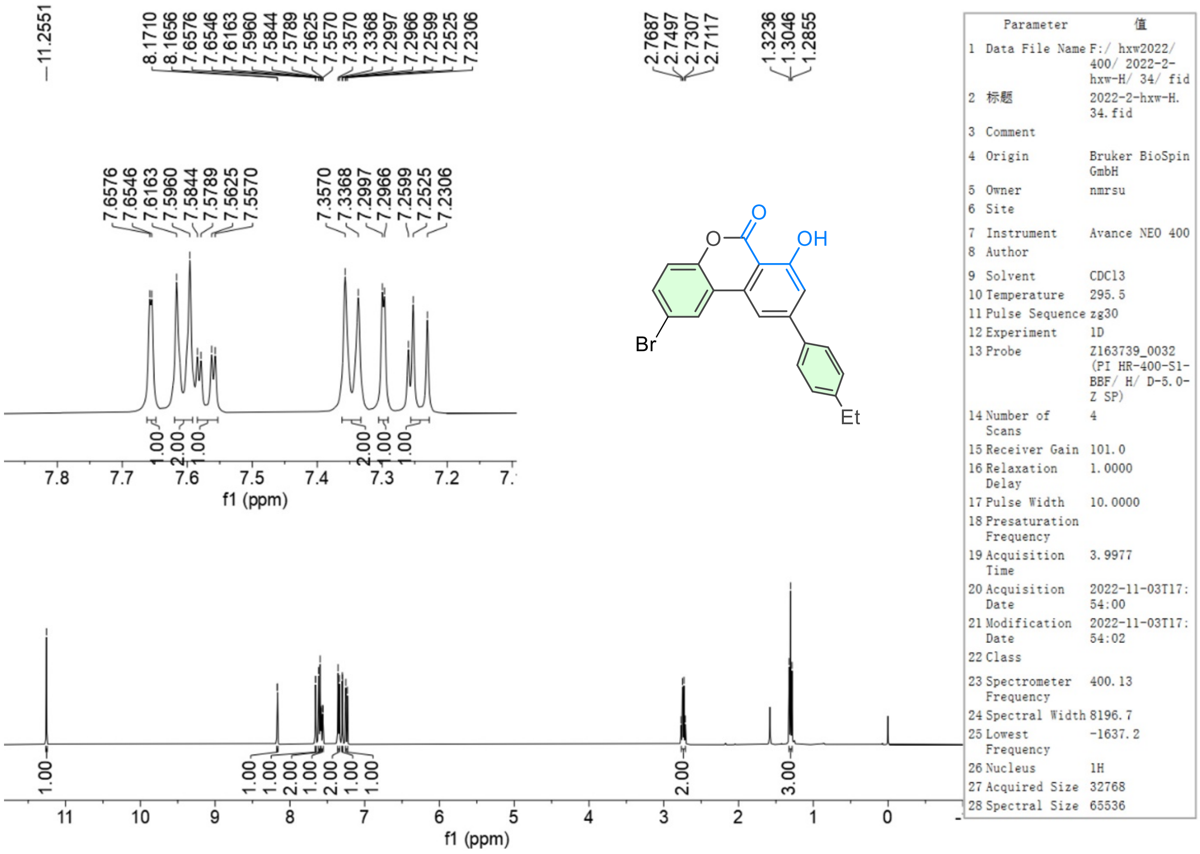

Parameter	值
1 Data File Name	F:/ hxw2022/ 400/ 2022-2-hxw-H/ 34/ fid
2 标题	2022-2-hxw-H. 34. fid
3 Comment	
4 Origin	Bruker BioSpin GmbH
5 Owner	nmrsu
6 Site	
7 Instrument	Avance NEO 400
8 Author	
9 Solvent	CDCl3
10 Temperature	295. 5
11 Pulse Sequence	zg30
12 Experiment	1D
13 Probe	Z163739_0032 (PI HR-400-S1-BBF/ H/ D-5. 0-Z SP)
14 Number of Scans	4
15 Receiver Gain	101. 0
16 Relaxation Delay	1. 0000
17 Pulse Width	10. 0000
18 Presaturation Frequency	
19 Acquisition Time	3. 9977
20 Acquisition Date	2022-11-03T17: 54:00
21 Modification Date	2022-11-03T17: 54:02
22 Class	
23 Spectrometer Frequency	400. 13
24 Spectral Width	8196. 7
25 Lowest Frequency	-1637. 2
26 Nucleus	1H
27 Acquired Size	32768
28 Spectral Size	65536

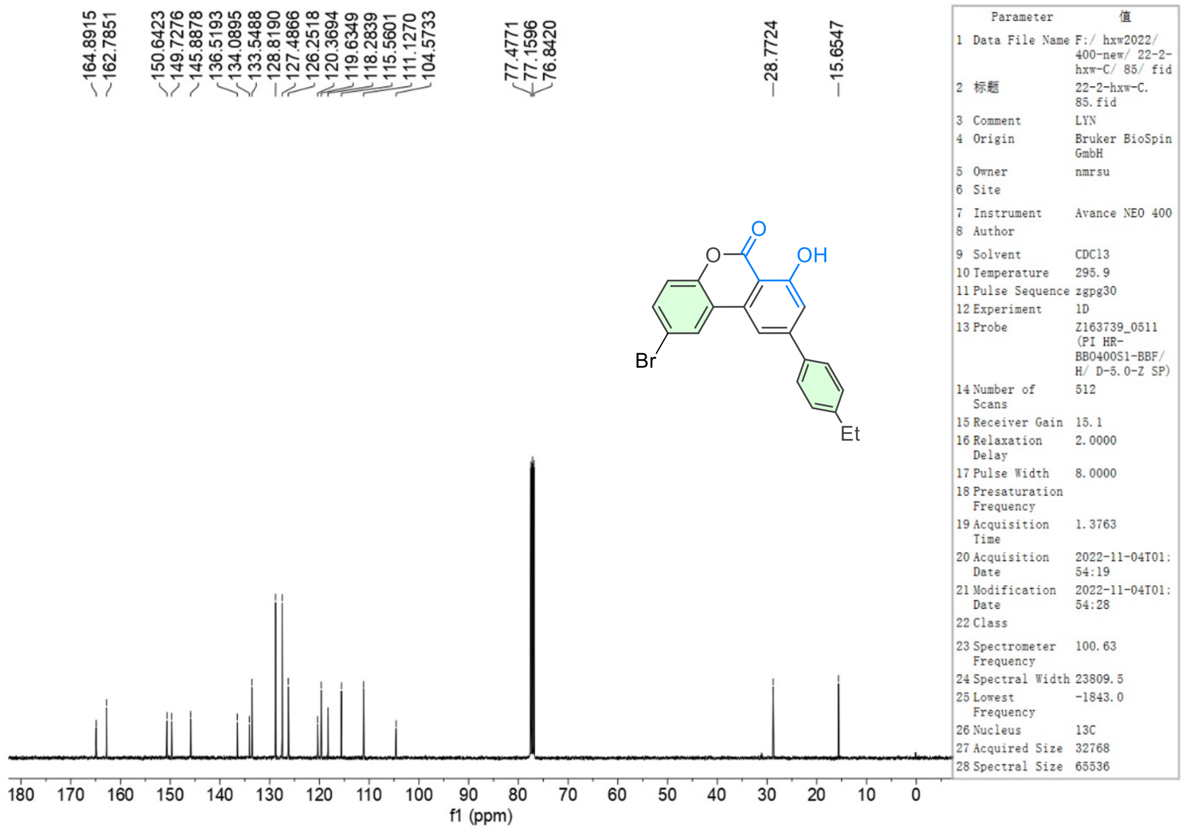

Parameter	值
1 Data File Name	F:/ hxw2022/ 400-new/ 22-2-hxw-C/ 85/ fid
2 标题	22-2-hxw-C. 85. fid
3 Comment	LYN
4 Origin	Bruker BioSpin GmbH
5 Owner	nmrsu
6 Site	
7 Instrument	Avance NEO 400
8 Author	
9 Solvent	CDCl3
10 Temperature	295. 9
11 Pulse Sequence	zgpg30
12 Experiment	1D
13 Probe	Z163739_0511 (PI HR-BB0400S1-BBF/ H/ D-5. 0-Z SP)
14 Number of Scans	512
15 Receiver Gain	15. 1
16 Relaxation Delay	2. 0000
17 Pulse Width	8. 0000
18 Presaturation Frequency	
19 Acquisition Time	1. 3763
20 Acquisition Date	2022-11-04T01: 54:19
21 Modification Date	2022-11-04T01: 54:28
22 Class	
23 Spectrometer Frequency	100. 63
24 Spectral Width	23809. 5
25 Lowest Frequency	-1843. 0
26 Nucleus	13C
27 Acquired Size	32768
28 Spectral Size	65536

2-Bromo-9-(4-chlorophenyl)-7-hydroxy-6*H*-benzo[*c*]chromen-6-one (product 3t)

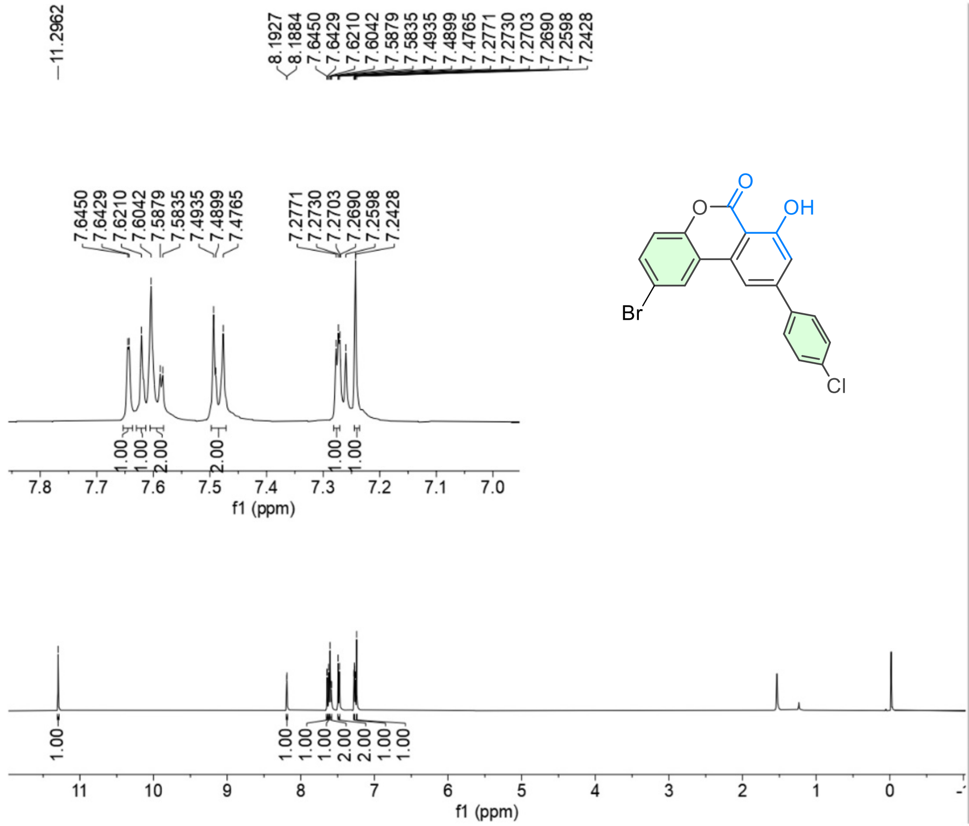

	Parameter	值
1	Data File Name	F:/ hxw2022/ 500/ 2022-1-hxw-H/ 37/ fid
2	标题	2022-1-hxw-H. 37. fid
3	Comment	1H lyn zi
4	Origin	Bruker BioSpin GmbH
5	Owner	nmr
6	Site	
7	Instrument	spect
8	Author	
9	Solvent	CDCl3
10	Temperature	297.1
11	Pulse Sequence	zg30
12	Experiment	1D
13	Probe	Z119470_0117 (PA BBO 500S1 BBF-H-D-05 Z SP)
14	Number of Scans	4
15	Receiver Gain	188.8
16	Relaxation Delay	2.0000
17	Pulse Width	12.0000
18	Presaturation Frequency	
19	Acquisition Time	1.8175
20	Acquisition Date	2022-10-18T21:23:12
21	Modification Date	2022-10-18T21:23:14
22	Class	
23	Spectrometer Frequency	500.16
24	Spectral Width	9014.4
25	Lowest Frequency	-1532.4
26	Nucleus	1H
27	Acquired Size	16384
28	Spectral Size	65536

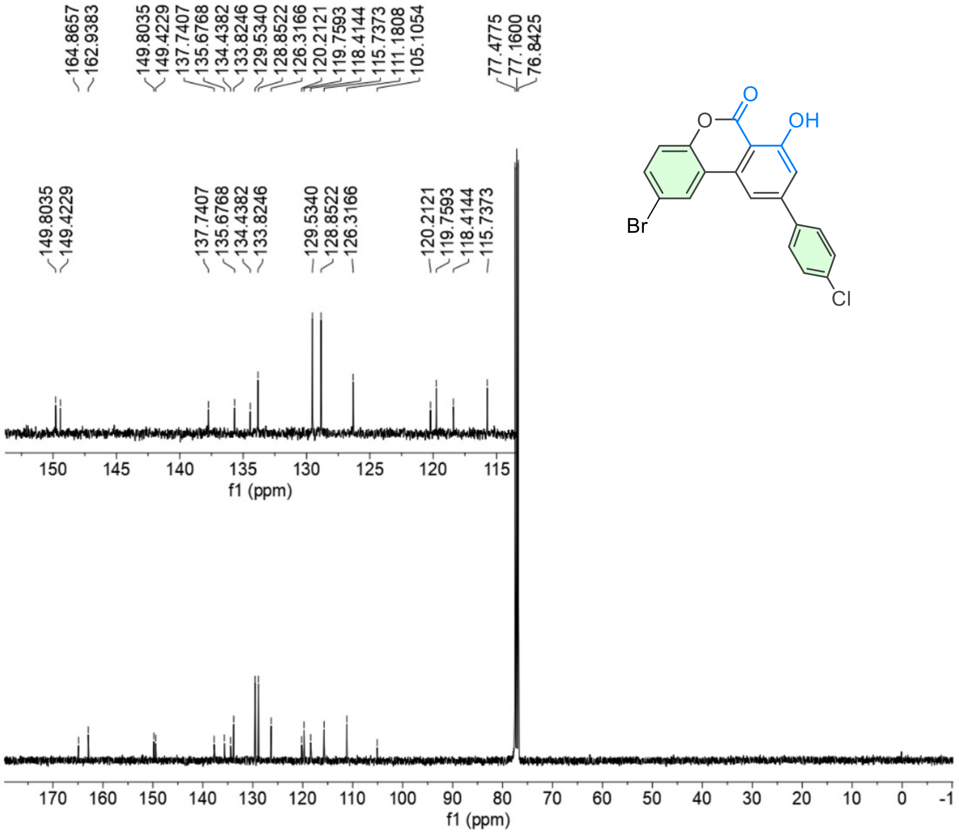

	Parameter	值
1	Data File Name	F:/ hxw2022/ 400-new/ 22-2-hxw-C/ 52/ fid
2	标题	22-2-hxw-C. 52. fid
3	Comment	
4	Origin	Bruker BioSpin GmbH
5	Owner	nmrsu
6	Site	
7	Instrument	Avance NEO 400
8	Author	
9	Solvent	CDCl3
10	Temperature	295.9
11	Pulse Sequence	zgpg30
12	Experiment	1D
13	Probe	Z163739_0511 (PI HR-BBO400S1-BBF/ H/ D-5.0-Z SP)
14	Number of Scans	512
15	Receiver Gain	15.1
16	Relaxation Delay	2.0000
17	Pulse Width	8.0000
18	Presaturation Frequency	
19	Acquisition Time	1.3763
20	Acquisition Date	2022-10-19T00:14:39
21	Modification Date	2022-10-19T00:14:42
22	Class	
23	Spectrometer Frequency	100.63
24	Spectral Width	23809.5
25	Lowest Frequency	-1843.0
26	Nucleus	13C
27	Acquired Size	32768
28	Spectral Size	65536

9-(4-Bromophenyl)-2-chloro-7-hydroxy-6*H*-benzo[*c*]chromen-6-one (product 3u)

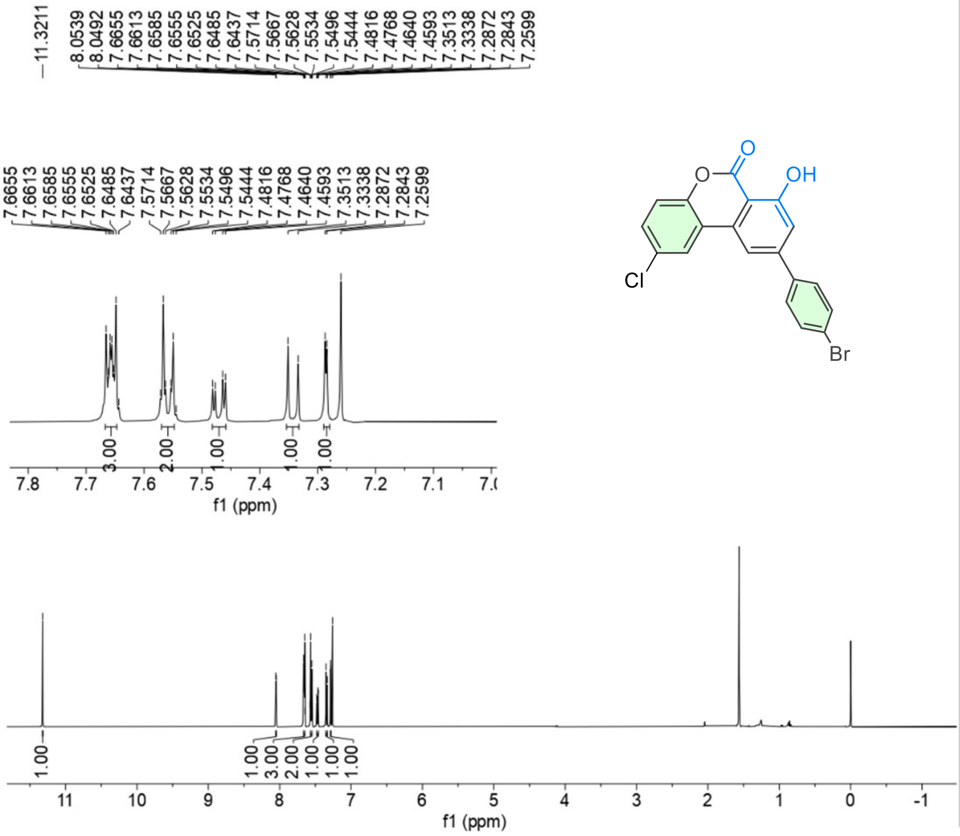

Parameter	值
1 Data File Name	F:/ hxw2022/ 500/ 2022-1-hxw-H/ 44/ fid
2 标题	2022-1-hxw-H. 44. fid
3 Comment	1H 1v
4 Origin	Bruker BioSpin GmbH
5 Owner	nmr
6 Site	
7 Instrument	spect
8 Author	
9 Solvent	CDCl3
10 Temperature	296. 9
11 Pulse Sequence	zg30
12 Experiment	1D
13 Probe	Z119470_0117 (PA BBO 500S1 BBF-H-D-05 Z SP)
14 Number of Scans	2
15 Receiver Gain	188. 8
16 Relaxation Delay	2. 0000
17 Pulse Width	12. 0000
18 Presaturation Frequency	
19 Acquisition Time	1. 8175
20 Acquisition Date	2022-11-16T21 :48:54
21 Modification Date	2022-11-16T21 :48:56
22 Class	
23 Spectrometer Frequency	500. 16
24 Spectral Width	9014. 4
25 Lowest Frequency	-1518. 4
26 Nucleus	1H
27 Acquired Size	16384
28 Spectral Size	65536

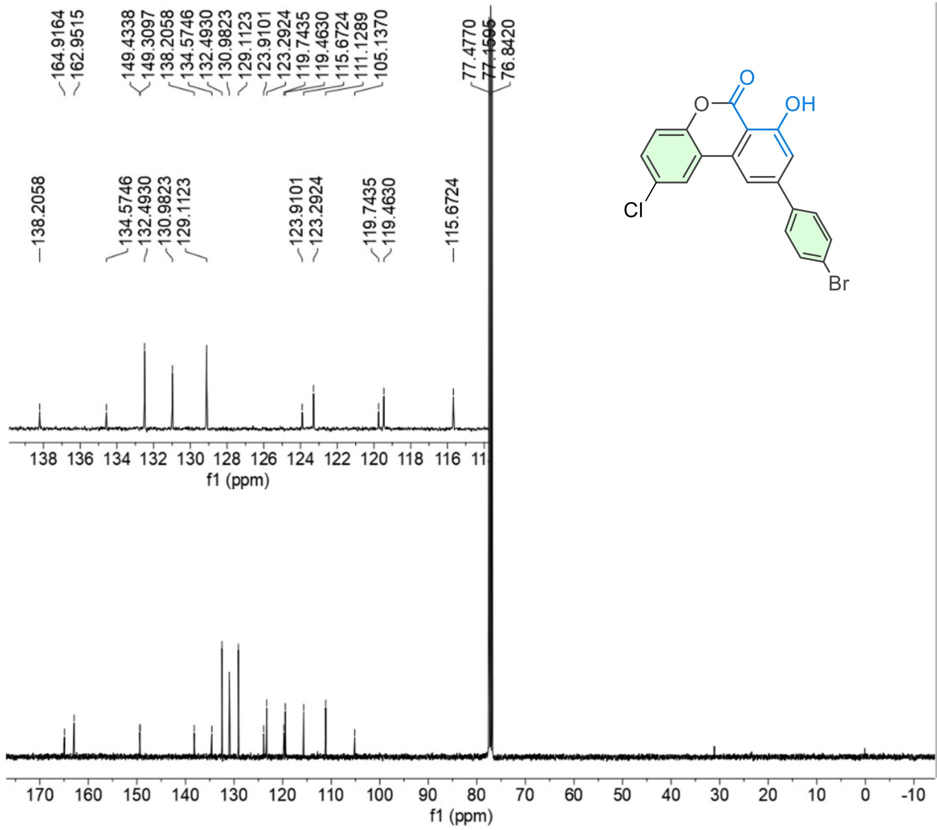

Parameter	值
1 Data File Name	F:/ hxw2022/ 22-2HXWC/ 124/ fid
2 标题	22-2-hxw-C. 124. fid
3 Comment	LYN-LV
4 Origin	Bruker BioSpin GmbH
5 Owner	nmrsu
6 Site	
7 Instrument	Avance NEO 400
8 Author	
9 Solvent	CDCl3
10 Temperature	295. 8
11 Pulse Sequence	zgpg30
12 Experiment	1D
13 Probe	Z163739_0511 (PI HR-BBO400S1-BBF/ H/ D-5. 0-Z SP)
14 Number of Scans	1024
15 Receiver Gain	14. 6
16 Relaxation Delay	2. 0000
17 Pulse Width	8. 0000
18 Presaturation Frequency	
19 Acquisition Time	1. 3763
20 Acquisition Date	2022-11-18T02: 19:19
21 Modification Date	2022-11-18T02: 19:36
22 Class	
23 Spectrometer Frequency	100. 63
24 Spectral Width	23809. 5
25 Lowest Frequency	-1798. 3
26 Nucleus	13C
27 Acquired Size	32768
28 Spectral Size	65536

9-(3-Bromophenyl)-7-hydroxy-6*H*-benzo[*c*]chromen-6-one (product 3v)

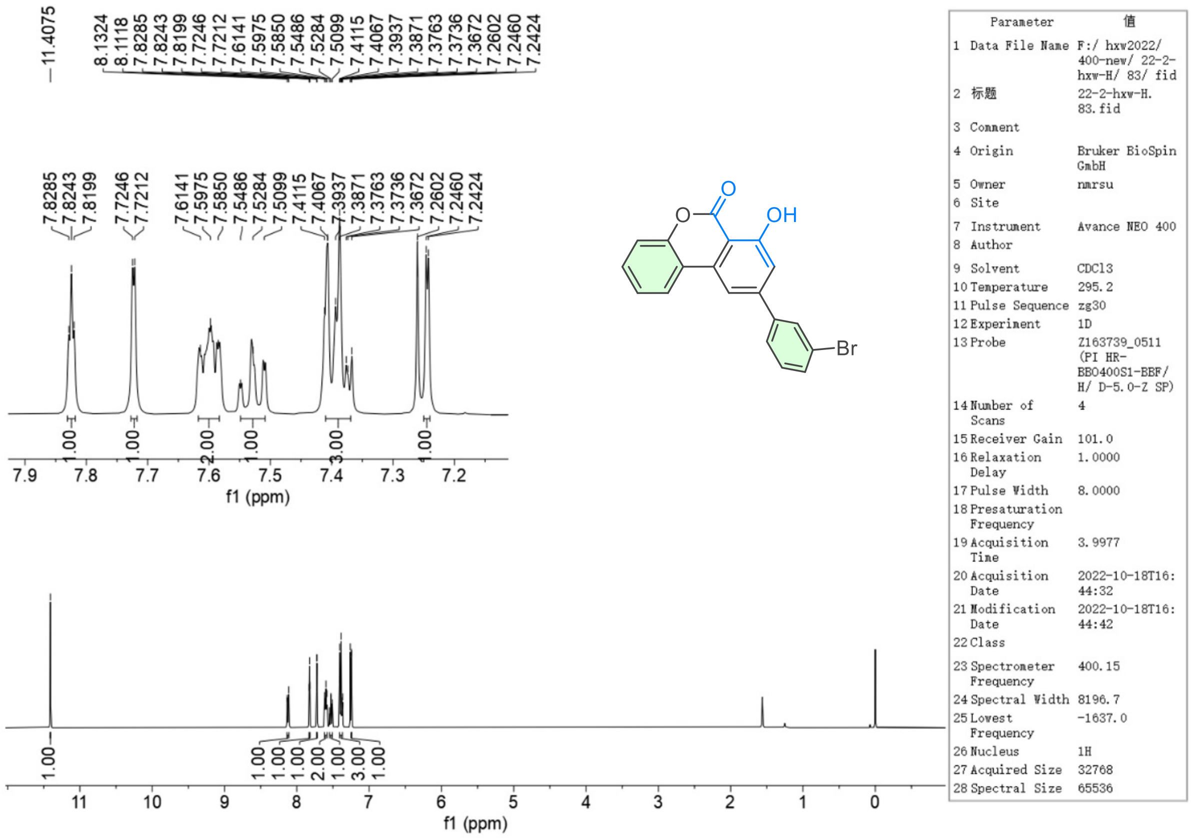

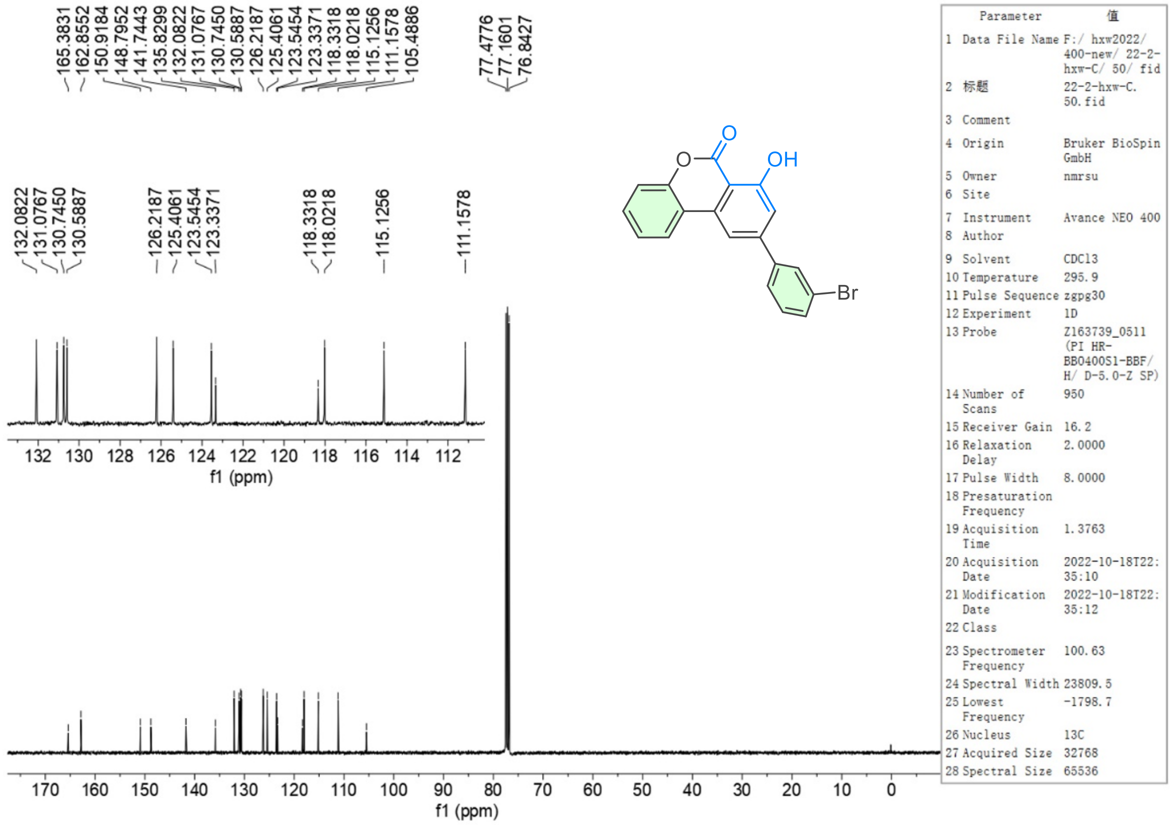

9-(3-Fluorophenyl)-7-hydroxy-6*H*-benzo[*c*]chromen-6-one (product 3w)

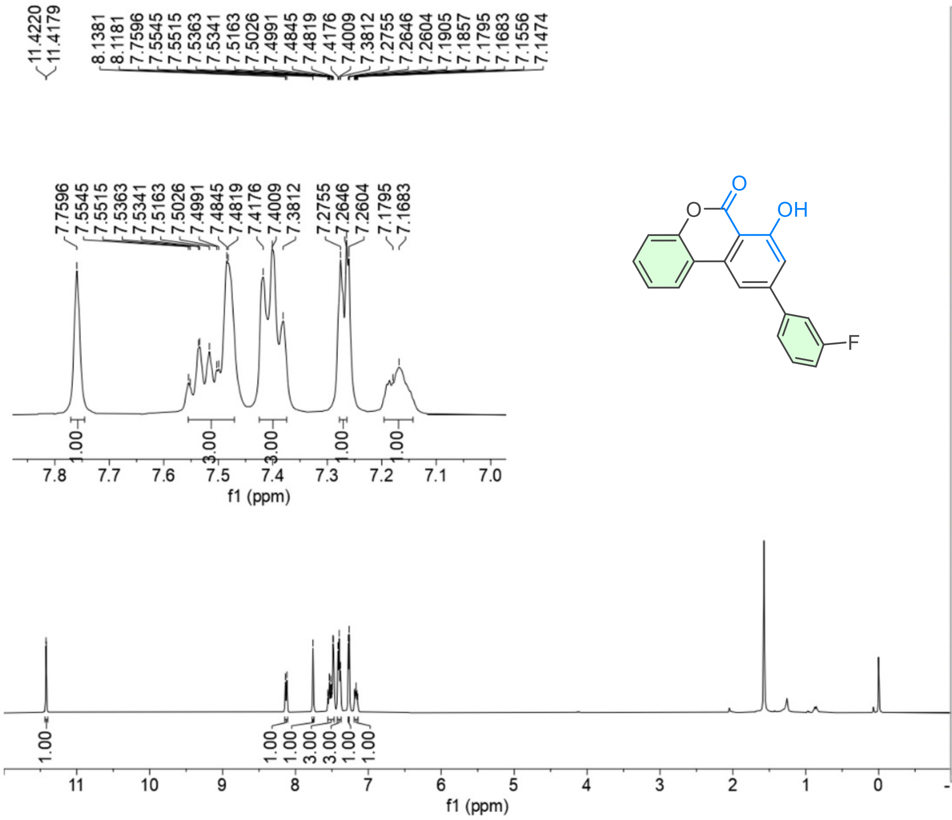

Parameter	值
1 Data File Name	F:/ hxw2022/ 22-2hxw-H/ 192/ fid
2 标题	22-2-hxw-H. 192. fid
3 Comment	
4 Origin	Bruker BioSpin GmbH
5 Owner	nmrsu
6 Site	
7 Instrument	Avance NEO 400
8 Author	
9 Solvent	CDCl3
10 Temperature	295. 3
11 Pulse Sequence	zg30
12 Experiment	1D
13 Probe	Z163739_0511 (PI HR-BB0400S1-BBF/ H/ D-5. 0-Z SP)
14 Number of Scans	4
15 Receiver Gain	101. 0
16 Relaxation Delay	1. 0000
17 Pulse Width	8. 0000
18 Presaturation Frequency	
19 Acquisition Time	3. 9977
20 Acquisition Date	2022-11-22T22: 10:54
21 Modification Date	2022-11-22T22: 11:14
22 Class	
23 Spectrometer Frequency	400. 15
24 Spectral Width	8196. 7
25 Lowest Frequency	-1636. 6
26 Nucleus	1H
27 Acquired Size	32768
28 Spectral Size	65536

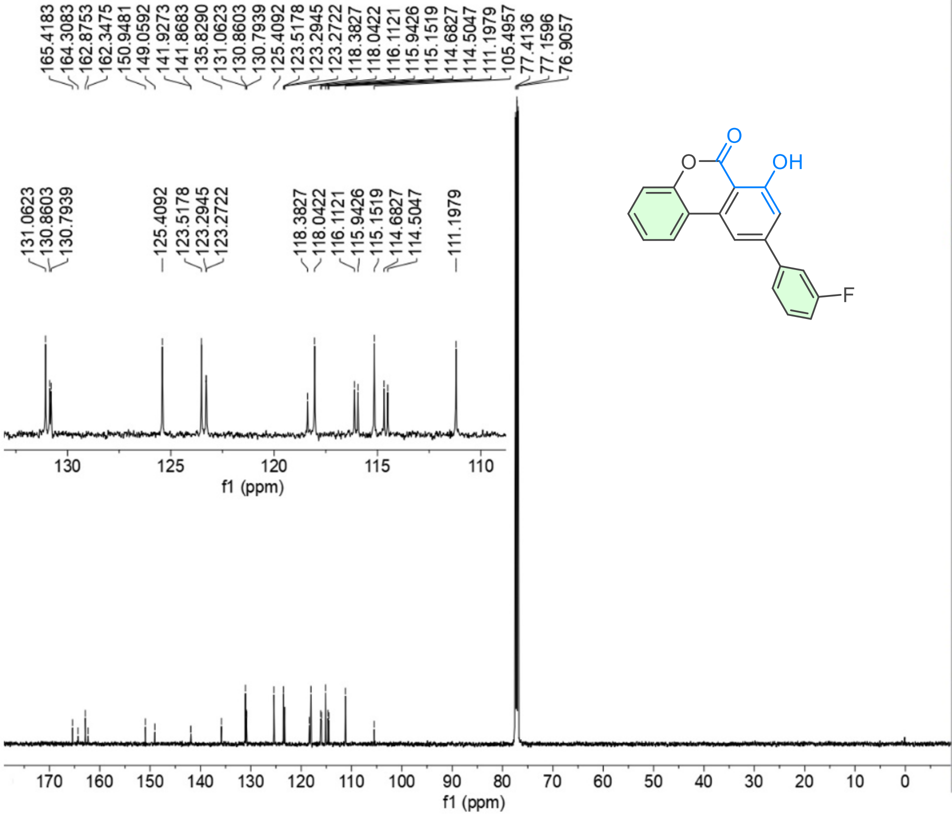

Parameter	值
1 Data File Name	F:/ hxw2022/ 500/ 2022-1-hxw-C/ 5/ fid
2 标题	2022-1-hxw-C. 5. fid
3 Comment	13C lyn red
4 Origin	Bruker BioSpin GmbH
5 Owner	nmr
6 Site	
7 Instrument	spect
8 Author	
9 Solvent	CDCl3
10 Temperature	296. 8
11 Pulse Sequence	zgpg30
12 Experiment	1D
13 Probe	Z119470_0117 (PA BBO 500S1 BBF-H-D-05 Z SP)
14 Number of Scans	1250
15 Receiver Gain	188. 8
16 Relaxation Delay	2. 0000
17 Pulse Width	10. 0000
18 Presaturation Frequency	
19 Acquisition Time	0. 4325
20 Acquisition Date	2022-11-25T20 :57:17
21 Modification Date	2022-11-25T20 :57:20
22 Class	
23 Spectrometer Frequency	125. 78
24 Spectral Width	37878. 8
25 Lowest Frequency	-5756. 4
26 Nucleus	13C
27 Acquired Size	16384
28 Spectral Size	65536

9-(3-Fluorophenyl)-7-hydroxy-6*H*-benzo[*c*]chromen-6-one (product 3w)

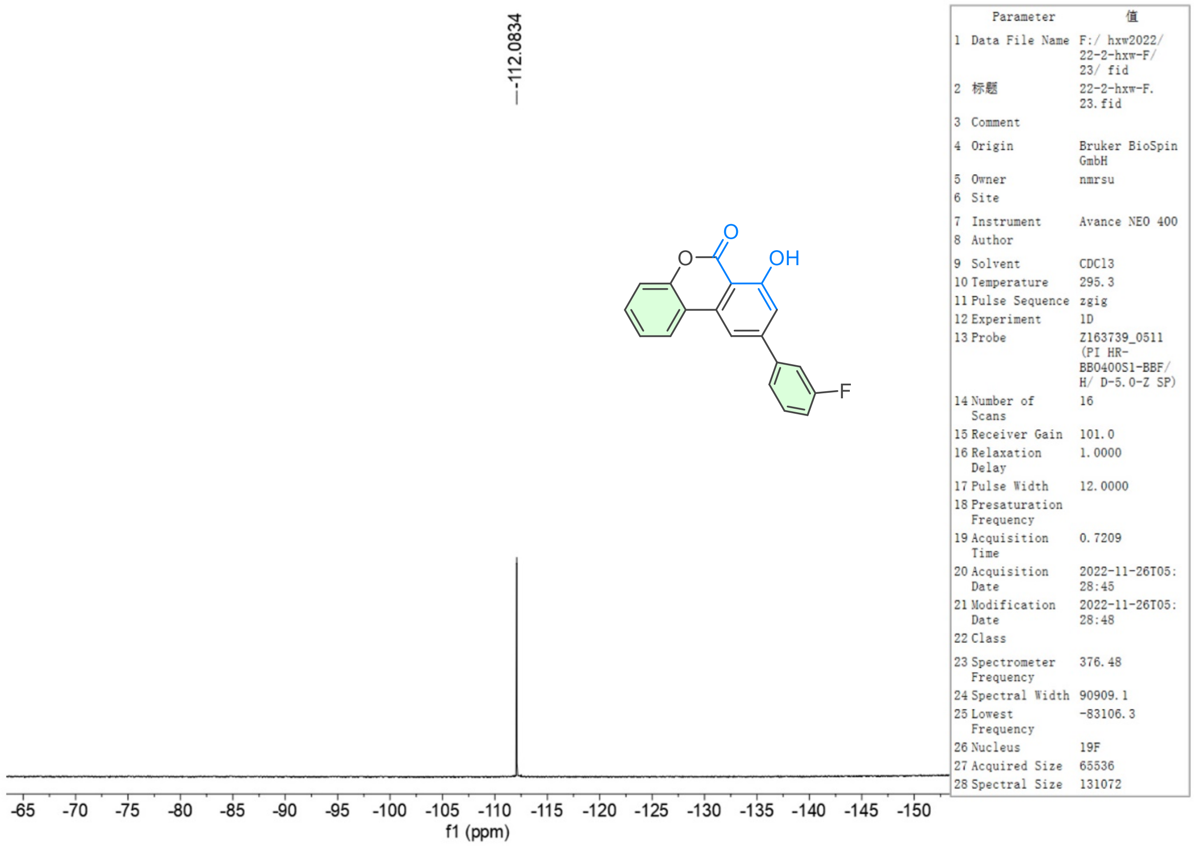

	Parameter	值
1	Data File Name	F:/ hxw2022/ 22-2-hxw-F/ 23/ fid
2	标题	22-2-hxw-F. 23.fid
3	Comment	
4	Origin	Bruker BioSpin GmbH
5	Owner	nmrsu
6	Site	
7	Instrument	Avance NEO 400
8	Author	
9	Solvent	CDCl3
10	Temperature	295.3
11	Pulse Sequence	zgig
12	Experiment	1D
13	Probe	Z163739_0511 (PI HR-BB0400S1-BBF/ H/ D-5.0-Z SP)
14	Number of Scans	16
15	Receiver Gain	101.0
16	Relaxation Delay	1.0000
17	Pulse Width	12.0000
18	Presaturation Frequency	
19	Acquisition Time	0.7209
20	Acquisition Date	2022-11-26T05:28:45
21	Modification Date	2022-11-26T05:28:48
22	Class	
23	Spectrometer Frequency	376.48
24	Spectral Width	90909.1
25	Lowest Frequency	-83106.3
26	Nucleus	19F
27	Acquired Size	65536
28	Spectral Size	131072

9-(3-Chlorophenyl)-7-hydroxy-6*H*-benzo[*c*]chromen-6-one (product 3x)

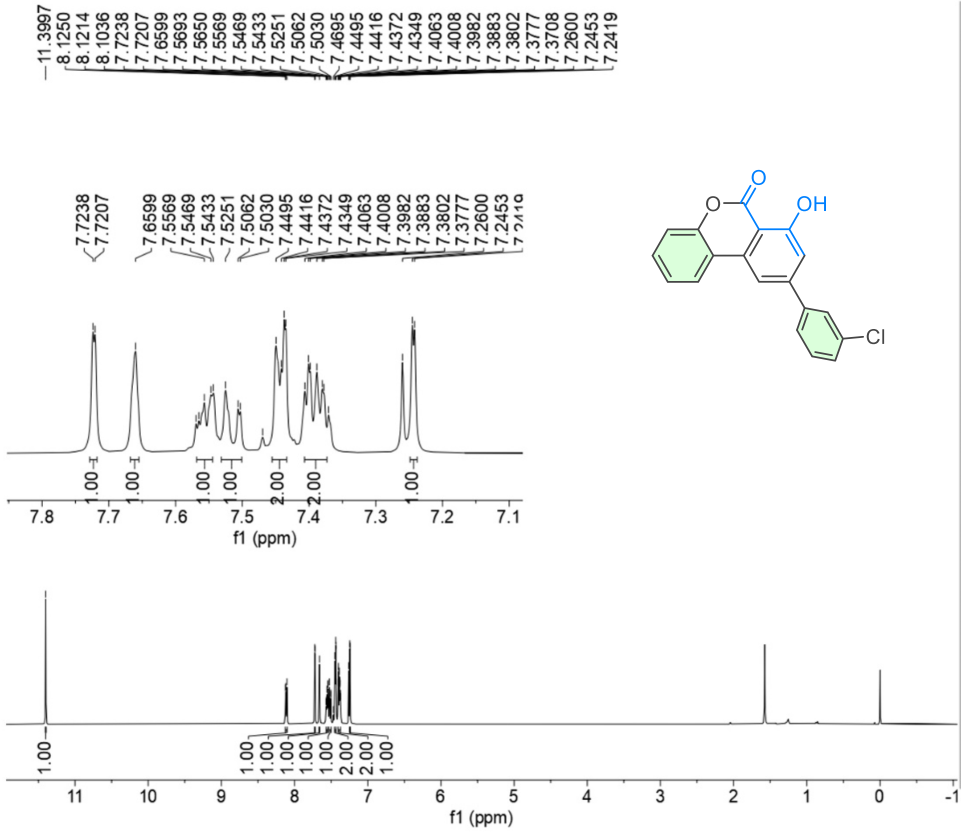

Parameter	值
1 Data File Name	F:/ hxw2022/ 400/ 2022-2-hxw-H/ 32/ fid
2 标题	2022-2-hxw-H. 32. fid
3 Comment	
4 Origin	Bruker BioSpin GmbH
5 Owner	nmrsu
6 Site	
7 Instrument	Avance NEO 400
8 Author	
9 Solvent	CDCl3
10 Temperature	295.6
11 Pulse Sequence	zg30
12 Experiment	1D
13 Probe	Z163739_0032 (PI HR-400-S1-BBF/ H/ D-5.0-Z SP)
14 Number of Scans	4
15 Receiver Gain	101.0
16 Relaxation Delay	1.0000
17 Pulse Width	10.0000
18 Presaturation Frequency	
19 Acquisition Time	3.9977
20 Acquisition Date	2022-11-03T17:44:52
21 Modification Date	2022-11-03T17:44:52
22 Class	
23 Spectrometer Frequency	400.13
24 Spectral Width	8196.7
25 Lowest Frequency	-1637.2
26 Nucleus	1H
27 Acquired Size	32768
28 Spectral Size	65536

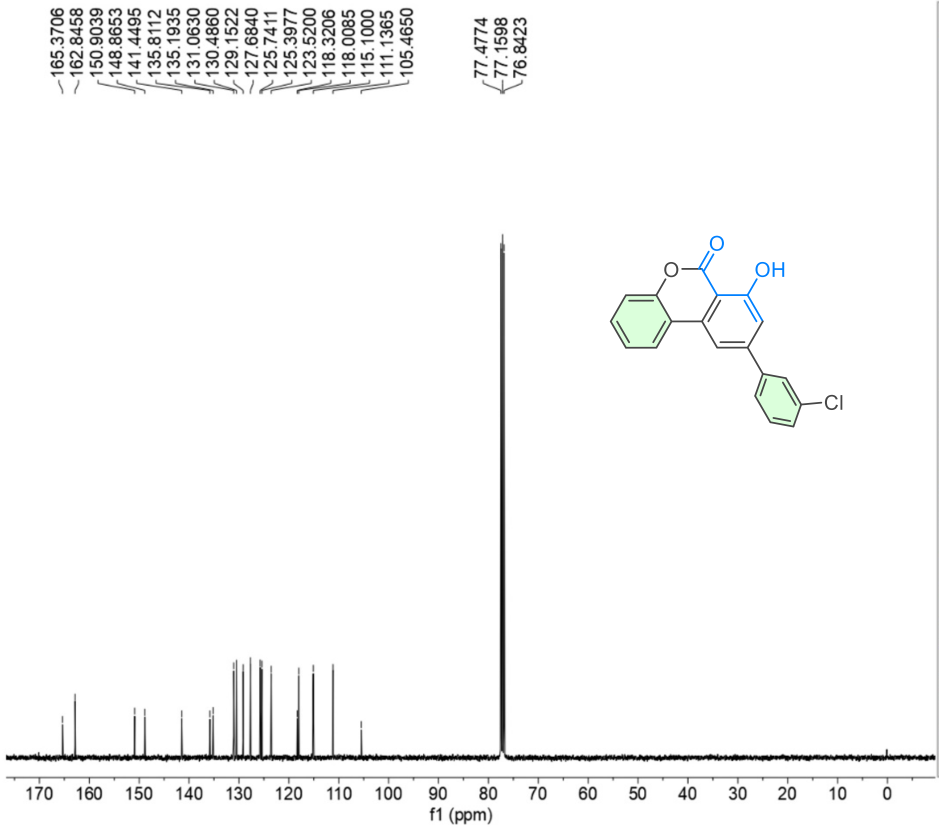

Parameter	值
1 Data File Name	F:/ hxw2022/ 400-new/ 22-2-hxw-C/ 88/ fid
2 标题	22-2-hxw-C. 88. fid
3 Comment	LYN
4 Origin	Bruker BioSpin GmbH
5 Owner	nmrsu
6 Site	
7 Instrument	Avance NEO 400
8 Author	
9 Solvent	CDCl3
10 Temperature	295.9
11 Pulse Sequence	zgpg30
12 Experiment	1D
13 Probe	Z163739_0511 (PI HR-BB0400S1-BBF/ H/ D-5.0-Z SP)
14 Number of Scans	512
15 Receiver Gain	15.1
16 Relaxation Delay	2.0000
17 Pulse Width	8.0000
18 Presaturation Frequency	
19 Acquisition Time	1.3763
20 Acquisition Date	2022-11-04T03:38:49
21 Modification Date	2022-11-04T03:38:56
22 Class	
23 Spectrometer Frequency	100.63
24 Spectral Width	23809.5
25 Lowest Frequency	-1799.0
26 Nucleus	13C
27 Acquired Size	32768
28 Spectral Size	65536

7-Hydroxy-9-(*m*-tolyl)-6*H*-benzo[*c*]chromen-6-one (product 3y)

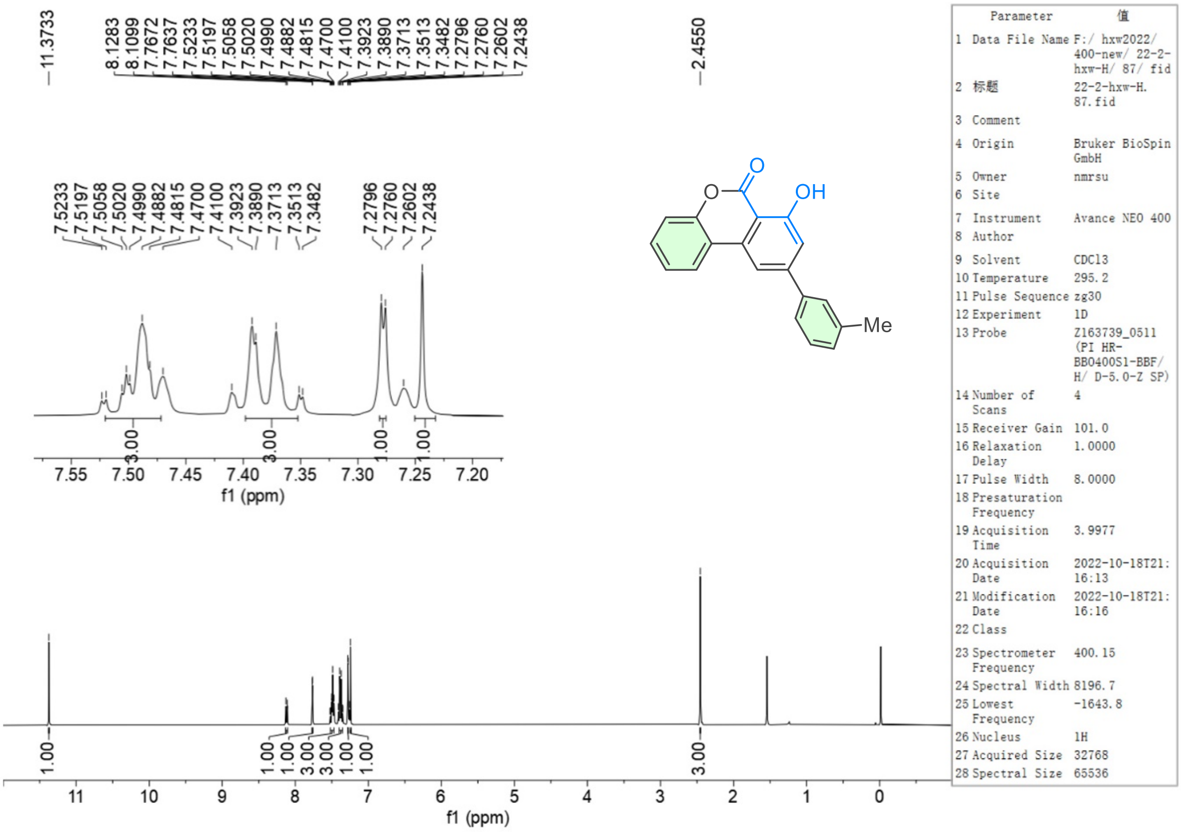

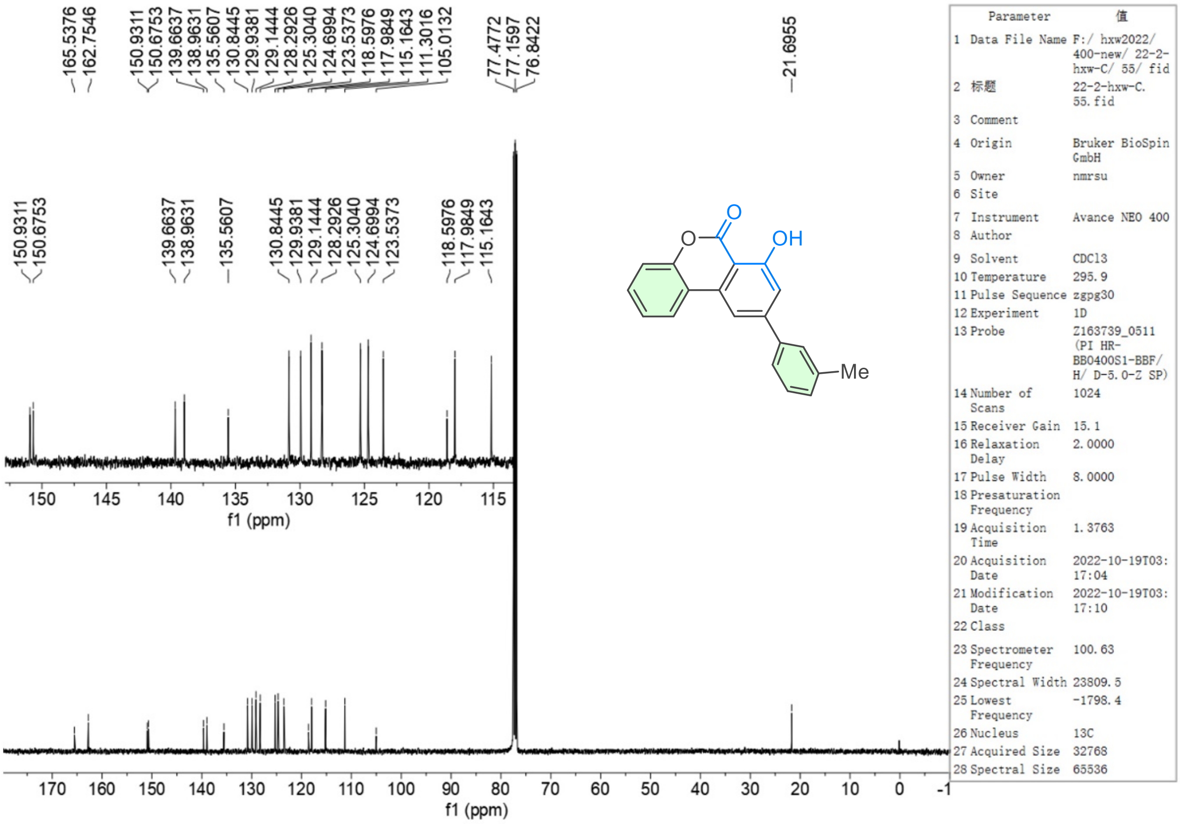

9-(2-Fluorophenyl)-7-hydroxy-6*H*-benzo[*c*]chromen-6-one (product 3z)

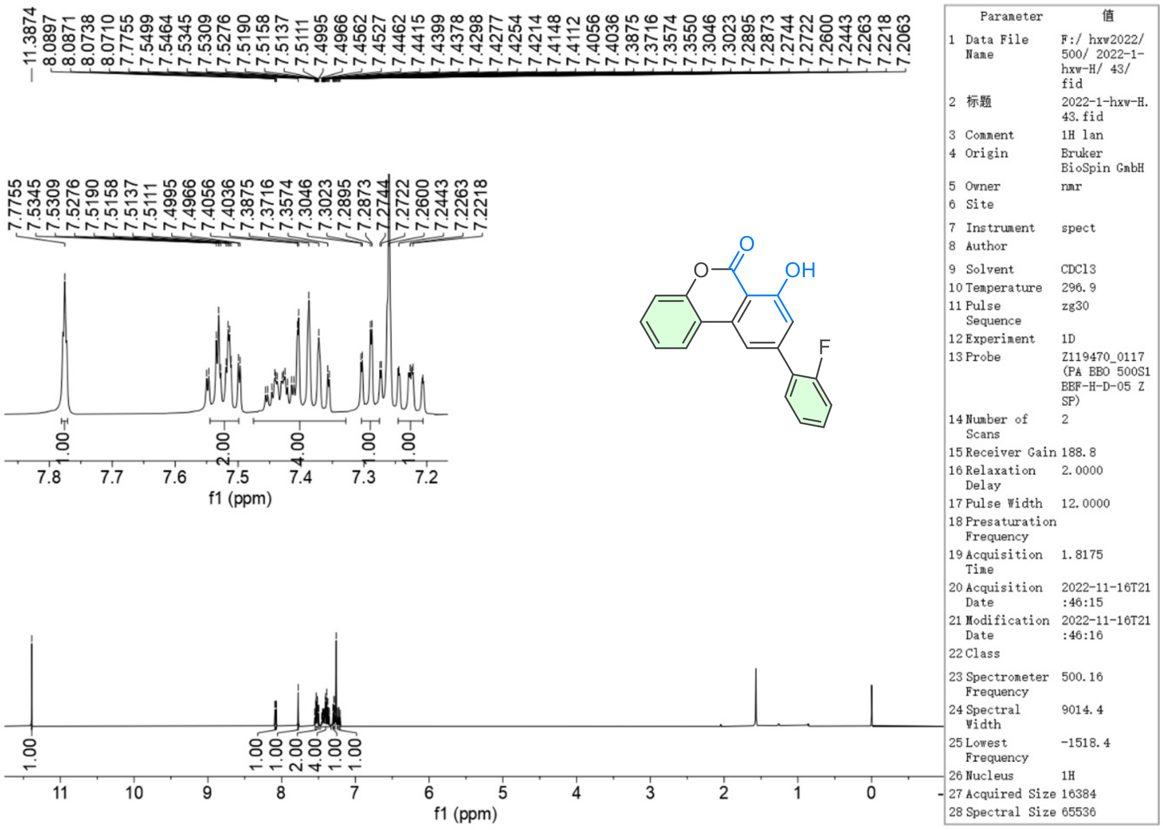

Parameter	值
1 Data File Name	F:/ hxw2022/ 500/ 2022-1-hxw-H/ 43/ fid
2 标题	2022-1-hxw-H. 43. fid
3 Comment	1H lan
4 Origin	Bruker BioSpin GmbH
5 Owner	nmr
6 Site	
7 Instrument	spect
8 Author	
9 Solvent	CDCl3
10 Temperature	296. 9
11 Pulse Sequence	zg30
12 Experiment	1D
13 Probe	Z119470_0117 (PA BBO 500S1 BBF-H-D-05 Z SP)
14 Number of Scans	2
15 Receiver Gain	188. 8
16 Relaxation Delay	2. 0000
17 Pulse Width	12. 0000
18 Presaturation Frequency	
19 Acquisition Time	1. 8175
20 Acquisition Date	2022-11-16T21 :46:15
21 Modification Date	2022-11-16T21 :46:16
22 Class	
23 Spectrometer Frequency	500. 16
24 Spectral Width	9014. 4
25 Lowest Frequency	-1518. 4
26 Nucleus	1H
27 Acquired Size	16384
28 Spectral Size	65536

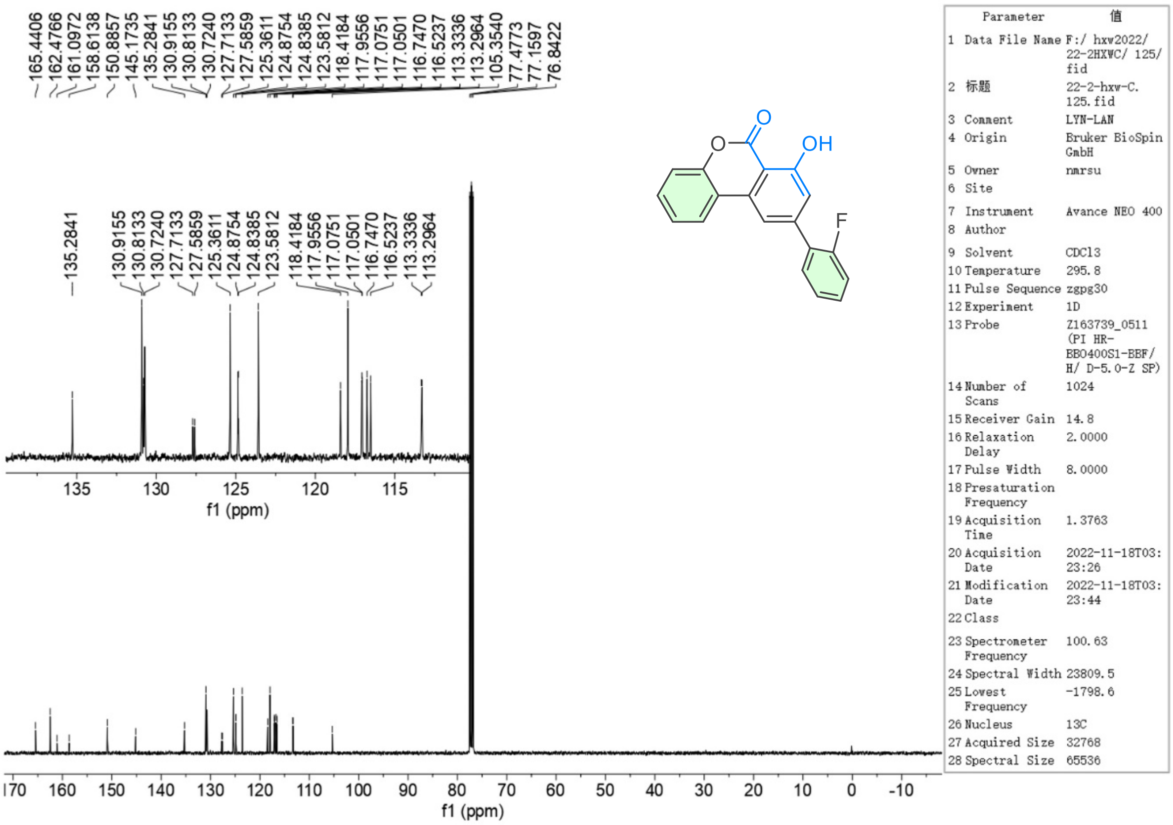

Parameter	值
1 Data File Name	F:/ hxw2022/ 22-2HXWC/ 125/ fid
2 标题	22-2-hxw-C. 125. fid
3 Comment	LYN-LAN
4 Origin	Bruker BioSpin GmbH
5 Owner	nmrsu
6 Site	
7 Instrument	Avance NEO 400
8 Author	
9 Solvent	CDCl3
10 Temperature	295. 8
11 Pulse Sequence	zgpg30
12 Experiment	1D
13 Probe	Z163739_0511 (PI HR-BBO400S1-BBF/ H/ D-5. 0-Z SP)
14 Number of Scans	1024
15 Receiver Gain	14. 8
16 Relaxation Delay	2. 0000
17 Pulse Width	8. 0000
18 Presaturation Frequency	
19 Acquisition Time	1. 3763
20 Acquisition Date	2022-11-18T03: 23:26
21 Modification Date	2022-11-18T03: 23:44
22 Class	
23 Spectrometer Frequency	100. 63
24 Spectral Width	23809. 5
25 Lowest Frequency	-1798. 6
26 Nucleus	13C
27 Acquired Size	32768
28 Spectral Size	65536

9-(2-Fluorophenyl)-7-hydroxy-6*H*-benzo[*c*]chromen-6-one (product 3z)

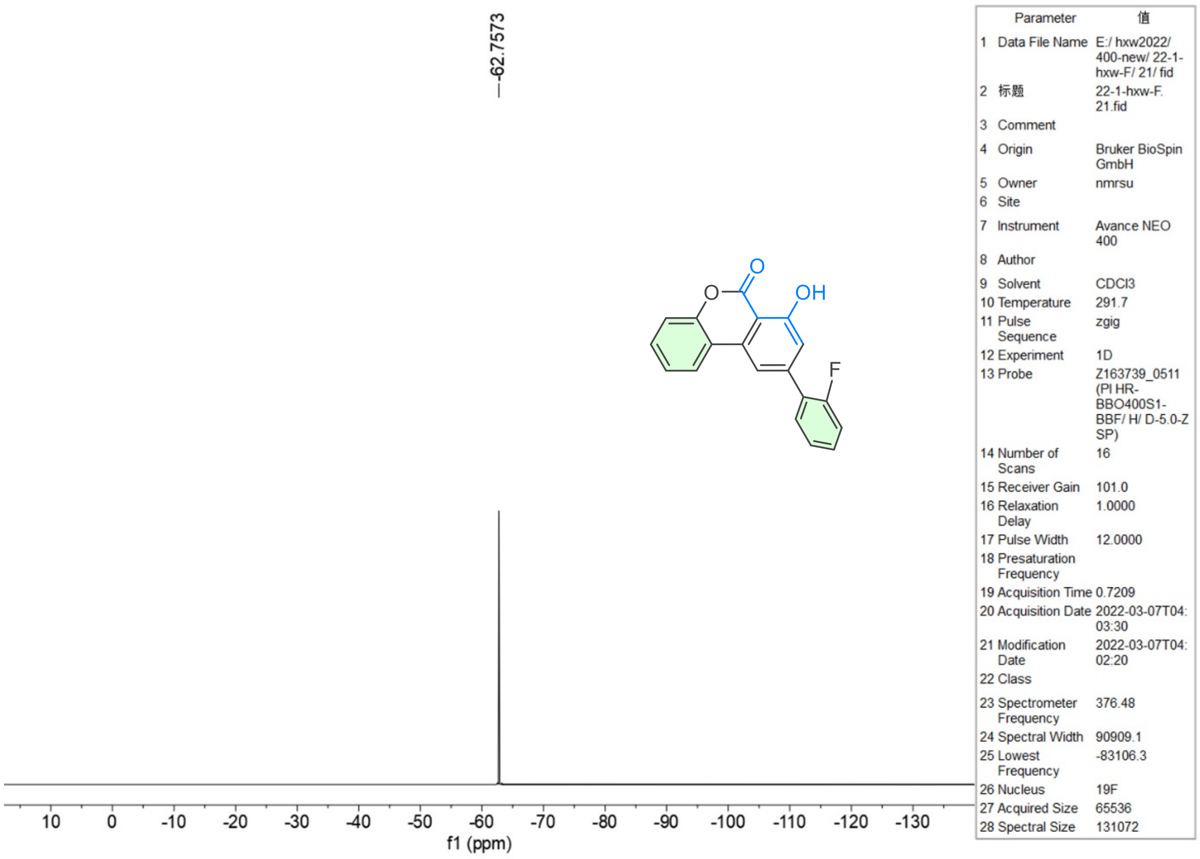

	Parameter	值
1	Data File Name	E:/ hxw2022/ 400-new/ 22-1-hxw-F/ 21/ fid
2	标题	22-1-hxw-F. 21.fid
3	Comment	
4	Origin	Bruker BioSpin GmbH
5	Owner	nmrsu
6	Site	
7	Instrument	Avance NEO 400
8	Author	
9	Solvent	CDCl3
10	Temperature	291.7
11	Pulse Sequence	zgig
12	Experiment	1D
13	Probe	Z163739_0511 (PI HR-BBO400S1-BBF/ H/ D-5.0-Z SP)
14	Number of Scans	16
15	Receiver Gain	101.0
16	Relaxation Delay	1.0000
17	Pulse Width	12.0000
18	Presaturation Frequency	
19	Acquisition Time	0.7209
20	Acquisition Date	2022-03-07T04:03:30
21	Modification Date	2022-03-07T04:02:20
22	Class	
23	Spectrometer Frequency	376.48
24	Spectral Width	90909.1
25	Lowest Frequency	-83106.3
26	Nucleus	19F
27	Acquired Size	65536
28	Spectral Size	131072

7-Hydroxy-9-(naphthalen-1-yl)-6*H*-benzo[*c*]chromen-6-one (product 3A)

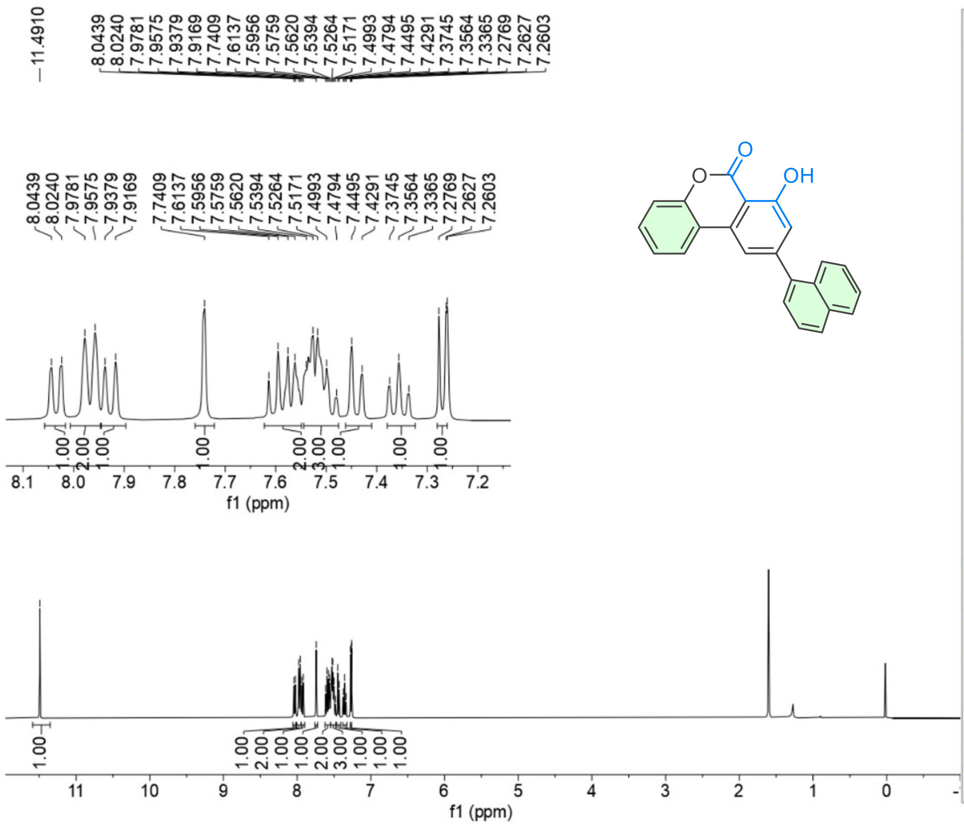

	Parameter	值
1	Data File Name	E:/ 2023 hxw0627/ 23-1-hxw-H/ 237/ fid
2	标题	23-1-hxw-H. 237.fid
3	Comment	
4	Origin	Bruker BioSpin GmbH
5	Owner	nmrsu
6	Site	
7	Instrument	Avance NEO 400
8	Author	
9	Solvent	CDCl3
10	Temperature	295.2
11	Pulse Sequence	zg30
12	Experiment	1D
13	Probe	Z163739_0511 (PI HR-BBO400S1-BBF/ H/ D-5.0-Z SP)
14	Number of Scans	4
15	Receiver Gain	101.0
16	Relaxation Delay	1.0000
17	Pulse Width	8.0000
18	Presaturation Frequency	
19	Acquisition Time	3.9977
20	Acquisition Date	2023-06-24T01:13:01
21	Modification Date	2023-06-24T01:13:18
22	Class	
23	Spectrometer Frequency	400.15
24	Spectral Width	8196.7
25	Lowest Frequency	-1630.6
26	Nucleus	1H
27	Acquired Size	32768
28	Spectral Size	65536

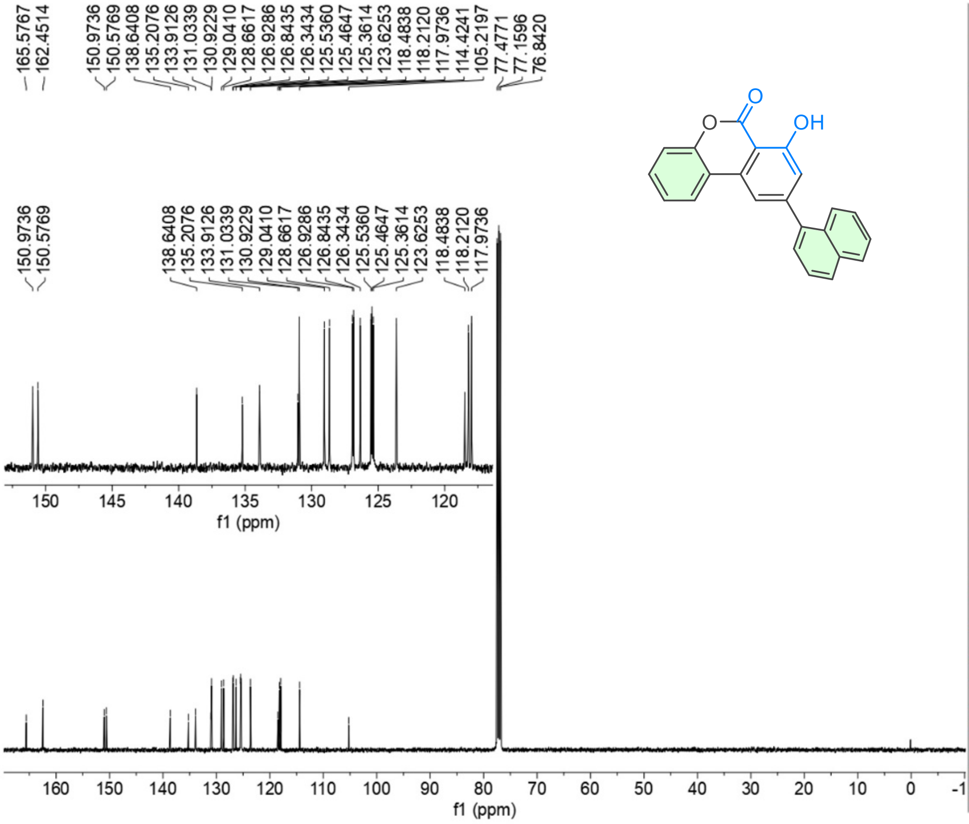

	Parameter	值
1	Data File Name	E:/ 2023 hxw0627/ 23-1-hxw-C/ 238/ fid
2	标题	23-1-hxw-H. 238.fid
3	Comment	
4	Origin	Bruker BioSpin GmbH
5	Owner	nmrsu
6	Site	
7	Instrument	Avance NEO 400
8	Author	
9	Solvent	CDCl3
10	Temperature	295.8
11	Pulse Sequence	zgpg30
12	Experiment	1D
13	Probe	Z163739_0511 (PI HR-BBO400S1-BBF/ H/ D-5.0-Z SP)
14	Number of Scans	1024
15	Receiver Gain	24.0
16	Relaxation Delay	2.0000
17	Pulse Width	8.0000
18	Presaturation Frequency	
19	Acquisition Time	1.3763
20	Acquisition Date	2023-06-24T02:13:37
21	Modification Date	2023-06-24T02:13:54
22	Class	
23	Spectrometer Frequency	100.63
24	Spectral Width	23809.5
25	Lowest Frequency	-1831.2
26	Nucleus	13C
27	Acquired Size	32768
28	Spectral Size	65536

7-Hydroxy-9-(thiophen-3-yl)-6*H*-benzo[*c*]chromen-6-one (product 3B)

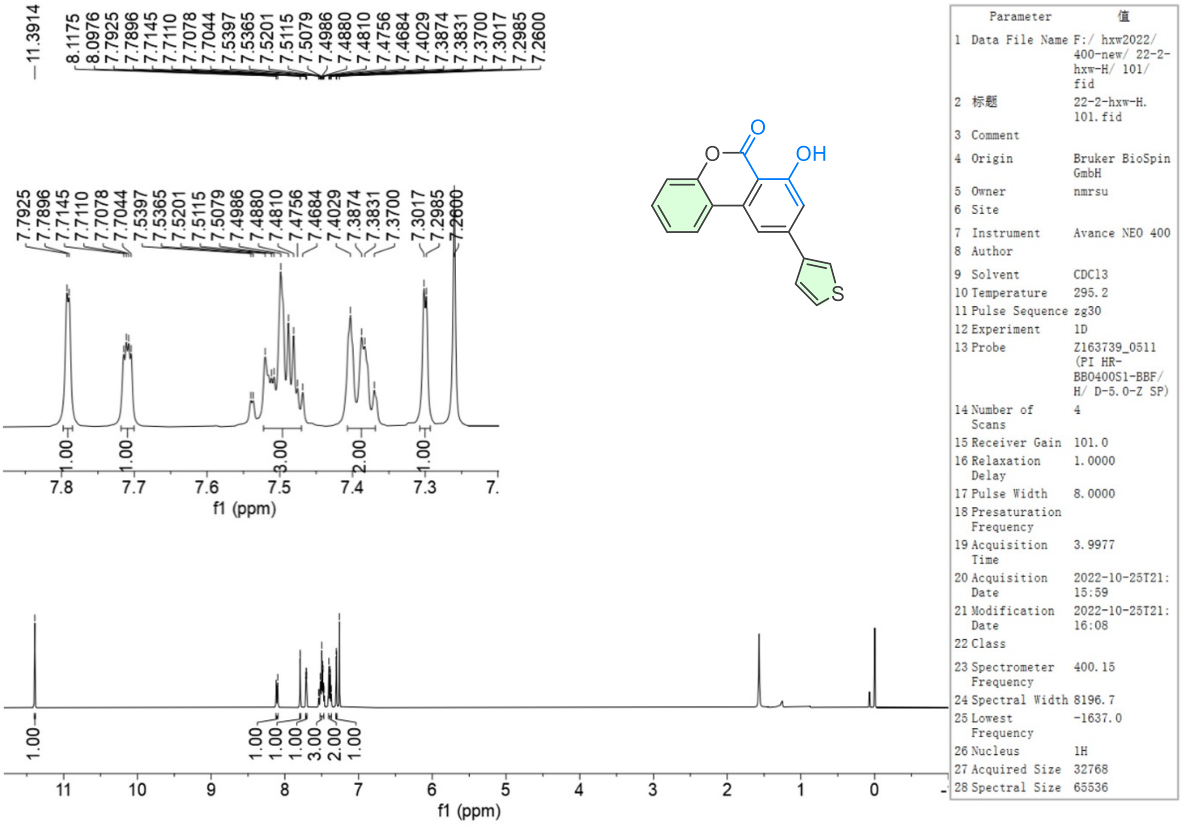

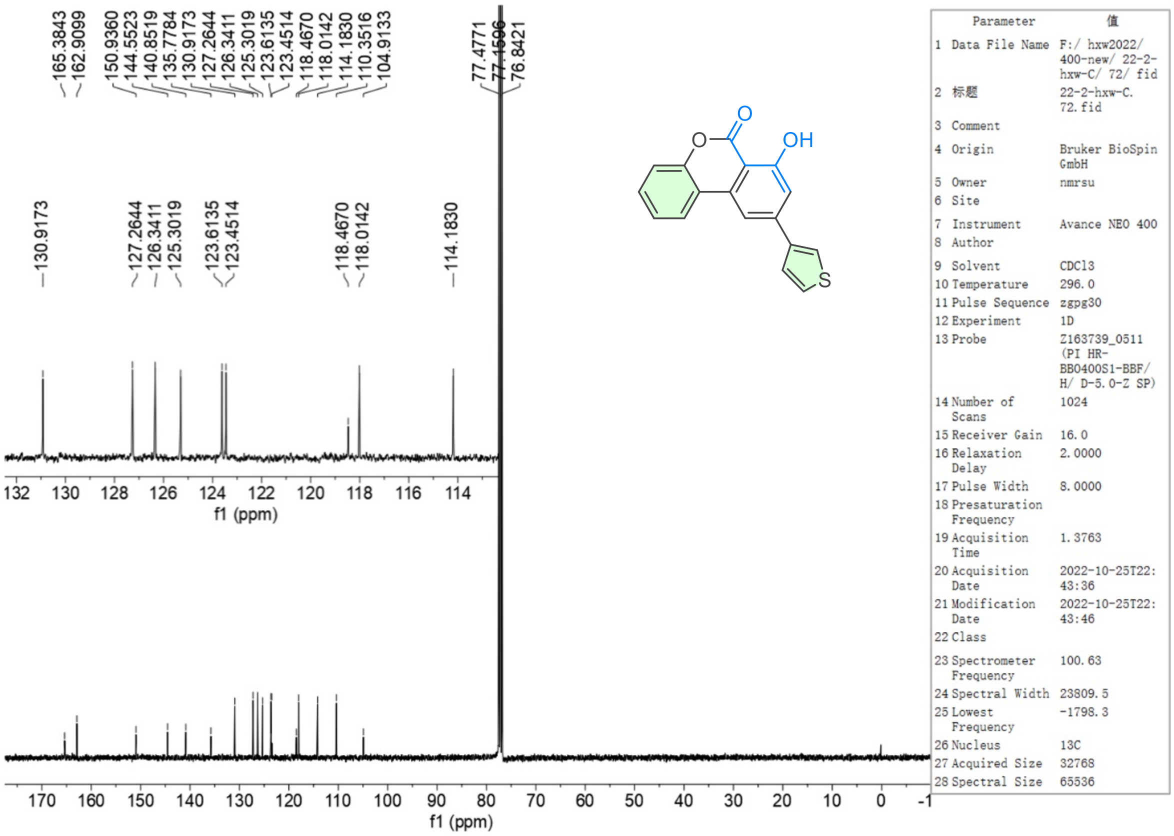

7-Hydroxy-9-(thiophen-2-yl)-6*H*-benzo[*c*]chromen-6-one (product 3C)

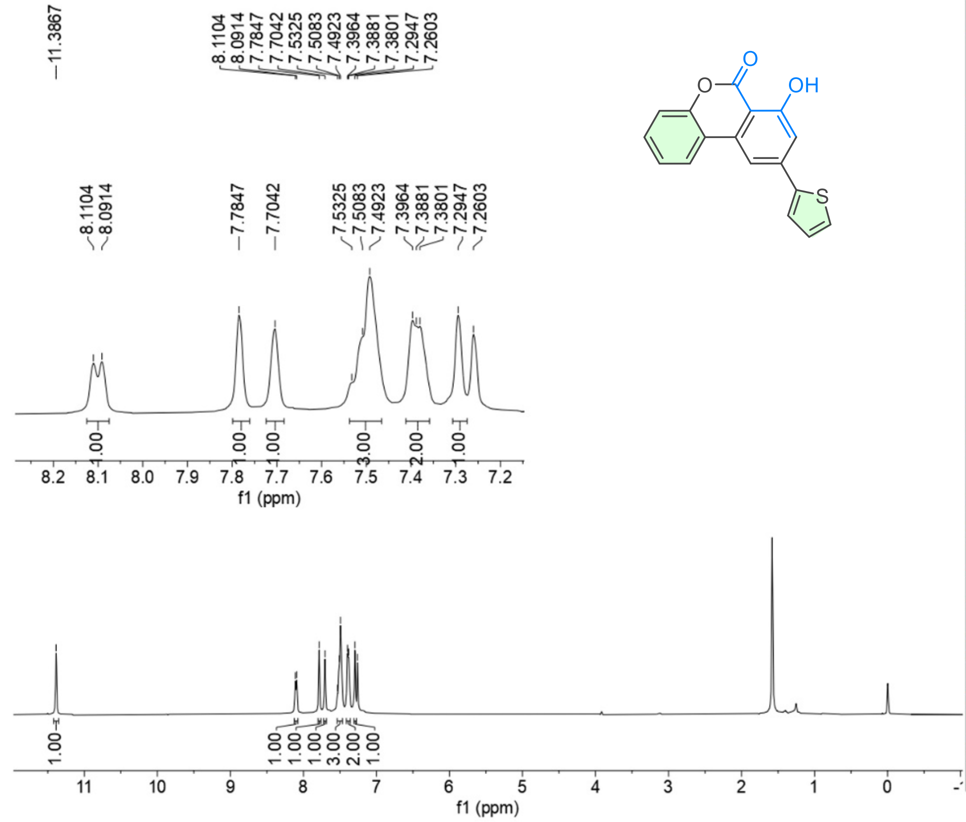

Parameter	值
1 Data File Name	F:/ hxw2022/ 400-new/ 22-2-hxw-H/ 150/ fid
2 标题	22-2-hxw-H. 150. fid
3 Comment	
4 Origin	Bruker BioSpin GmbH
5 Owner	nmrsu
6 Site	
7 Instrument	Avance NEO 400
8 Author	
9 Solvent	CDCl3
10 Temperature	295. 7
11 Pulse Sequence	zg30
12 Experiment	1D
13 Probe	Z163739_0511 (PI HR-BBO400S1-BBF/ H/ D-5. 0-Z SP)
14 Number of Scans	4
15 Receiver Gain	101. 0
16 Relaxation Delay	1. 0000
17 Pulse Width	8. 0000
18 Presaturation Frequency	
19 Acquisition Time	3. 9977
20 Acquisition Date	2022-11-09T21:54:11
21 Modification Date	2022-11-09T21:54:22
22 Class	
23 Spectrometer Frequency	400. 15
24 Spectral Width	8196. 7
25 Lowest Frequency	-1637. 4
26 Nucleus	1H
27 Acquired Size	32768
28 Spectral Size	65536

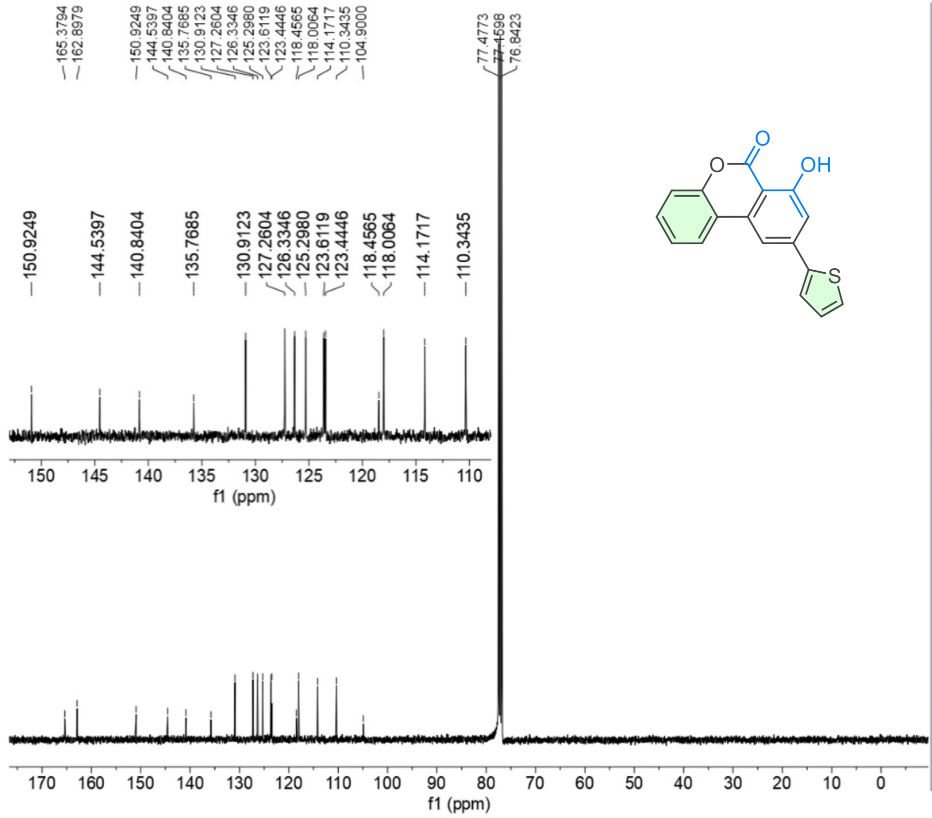

Parameter	值
1 Data File Name	F:/ hxw2022/ 400-new/ 22-2-hxw-C/ 100/ fid
2 标题	22-2-hxw-C. 100. fid
3 Comment	
4 Origin	Bruker BioSpin GmbH
5 Owner	nmrsu
6 Site	
7 Instrument	Avance NEO 400
8 Author	
9 Solvent	CDCl3
10 Temperature	295. 8
11 Pulse Sequence	zgpg30
12 Experiment	1D
13 Probe	Z163739_0511 (PI HR-BBO400S1-BBF/ H/ D-5. 0-Z SP)
14 Number of Scans	1024
15 Receiver Gain	16. 2
16 Relaxation Delay	2. 0000
17 Pulse Width	8. 0000
18 Presaturation Frequency	
19 Acquisition Time	1. 3763
20 Acquisition Date	2022-11-10T04:22:41
21 Modification Date	2022-11-10T04:22:54
22 Class	
23 Spectrometer Frequency	100. 63
24 Spectral Width	23809. 5
25 Lowest Frequency	-1798. 6
26 Nucleus	13C
27 Acquired Size	32768
28 Spectral Size	65536

7-Hydroxy-9-phenyl-6*H*-benzo[*c*]chromen-6-one-8,10-d$_2$ (product 3a-*d*$_2$)

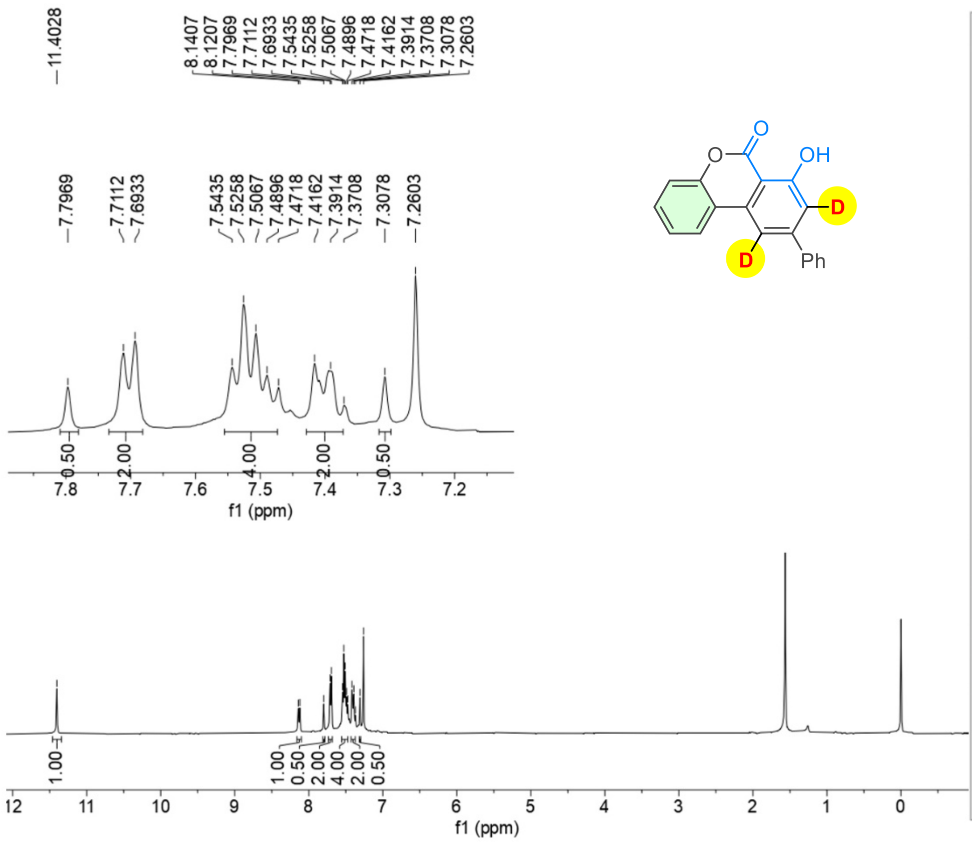

Parameter	值
1 Data File Name	E:/ 2023 hxw0627/ 23-1-hxw-H/ 42/ fid
2 标题	23-1-hxw-H. 42.fid
3 Comment	
4 Origin	Bruker BioSpin GmbH
5 Owner	nmrsu
6 Site	
7 Instrument	Avance NEO 400
8 Author	
9 Solvent	CDCl3
10 Temperature	295.0
11 Pulse Sequence	zg30
12 Experiment	1D
13 Probe	Z163739_0511 (PI HR-BBO400S1-BBF/ H/ D-5.0-Z SP)
14 Number of Scans	4
15 Receiver Gain	101.0
16 Relaxation Delay	1.0000
17 Pulse Width	8.0000
18 Presaturation Frequency	
19 Acquisition Time	3.9977
20 Acquisition Date	2023-03-08T21:27:16
21 Modification Date	2023-03-08T21:26:22
22 Class	
23 Spectrometer Frequency	400.15
24 Spectral Width	8196.7
25 Lowest Frequency	-1637.0
26 Nucleus	1H
27 Acquired Size	32768
28 Spectral Size	65536

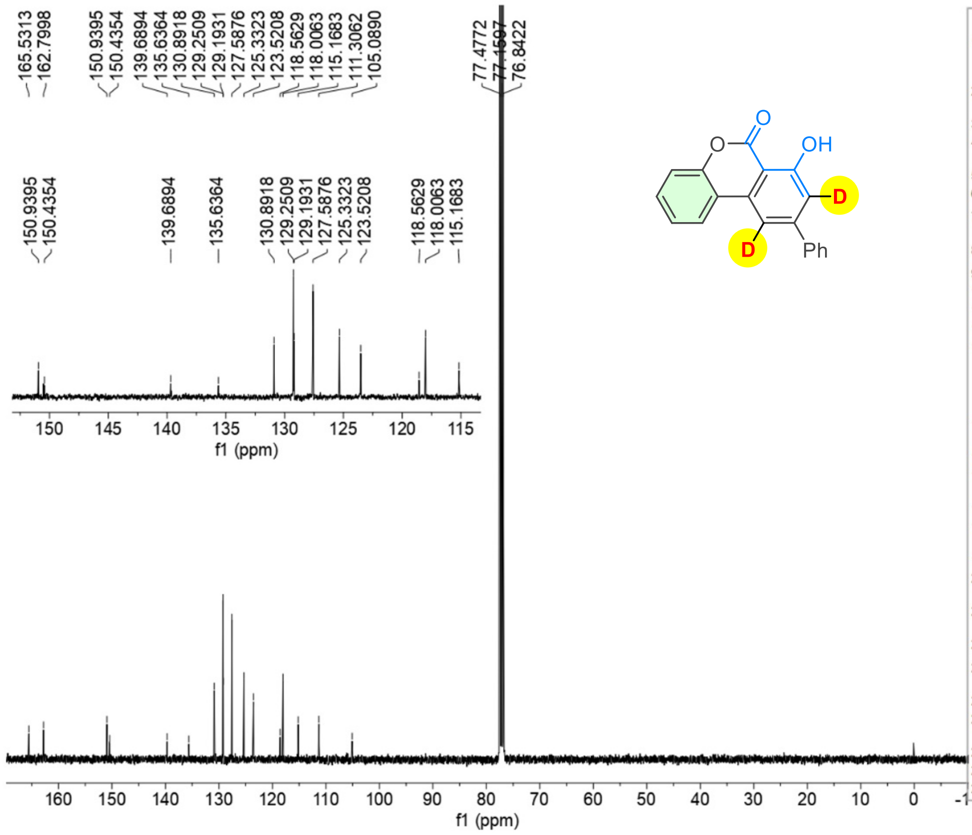

Parameter	值
1 Data File Name	E:/ 2023 hxw0627/ 23-1-hxw-C/ 137/ fid
2 标题	23-1-hxw-C. 137.fid
3 Comment	LYN-LV
4 Origin	Bruker BioSpin GmbH
5 Owner	nmrsu
6 Site	
7 Instrument	Avance NEO 400
8 Author	
9 Solvent	CDCl3
10 Temperature	295.6
11 Pulse Sequence	zgpg30
12 Experiment	1D
13 Probe	Z163739_0511 (PI HR-BBO400S1-BBF/ H/ D-5.0-Z SP)
14 Number of Scans	1024
15 Receiver Gain	14.6
16 Relaxation Delay	2.0000
17 Pulse Width	8.0000
18 Presaturation Frequency	
19 Acquisition Time	1.3763
20 Acquisition Date	2023-04-22T05:25:56
21 Modification Date	2023-04-22T05:26:20
22 Class	
23 Spectrometer Frequency	100.63
24 Spectral Width	23809.5
25 Lowest Frequency	-1830.2
26 Nucleus	13C
27 Acquired Size	32768
28 Spectral Size	65536

7-Methoxy-9-phenyl-6*H*-benzo[*c*]chromen-6-one (product 7)

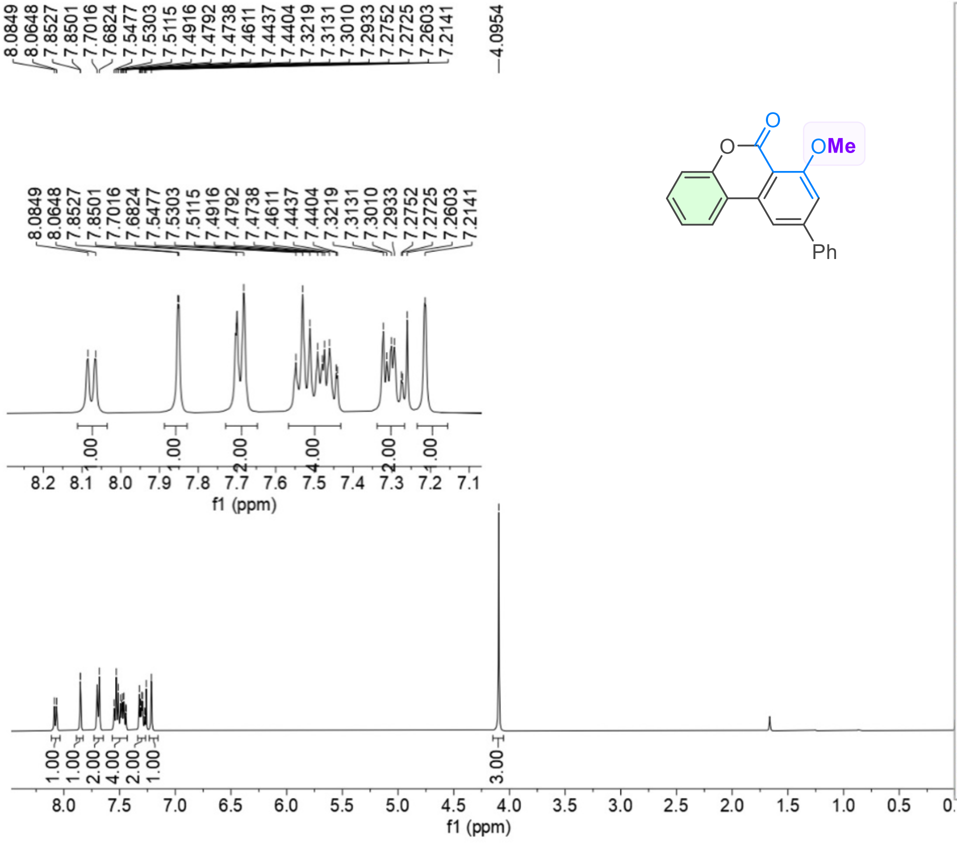

Parameter	值
1 Data File Name	E:/ 23-1-hxw-H/ 34/ fid
2 标题	23-1-hxw-H.34.fid
3 Comment	
4 Origin	Bruker BioSpin GmbH
5 Owner	nmrsu
6 Site	
7 Instrument	Avance NEO 400
8 Author	
9 Solvent	CDCl3
10 Temperature	295.3
11 Pulse Sequence	zg30
12 Experiment	1D
13 Probe	Z163739_0511 (PI HR-BBO400S1-BBF/ H/ D-5.0-Z SP)
14 Number of Scans	4
15 Receiver Gain	101.0
16 Relaxation Delay	1.0000
17 Pulse Width	8.0000
18 Presaturation Frequency	
19 Acquisition Time	3.9977
20 Acquisition Date	2023-03-03T21:45:54
21 Modification Date	2023-03-03T21:44:56
22 Class	
23 Spectrometer Frequency	400.15
24 Spectral Width	8196.7
25 Lowest Frequency	-1637.0
26 Nucleus	1H
27 Acquired Size	32768
28 Spectral Size	65536

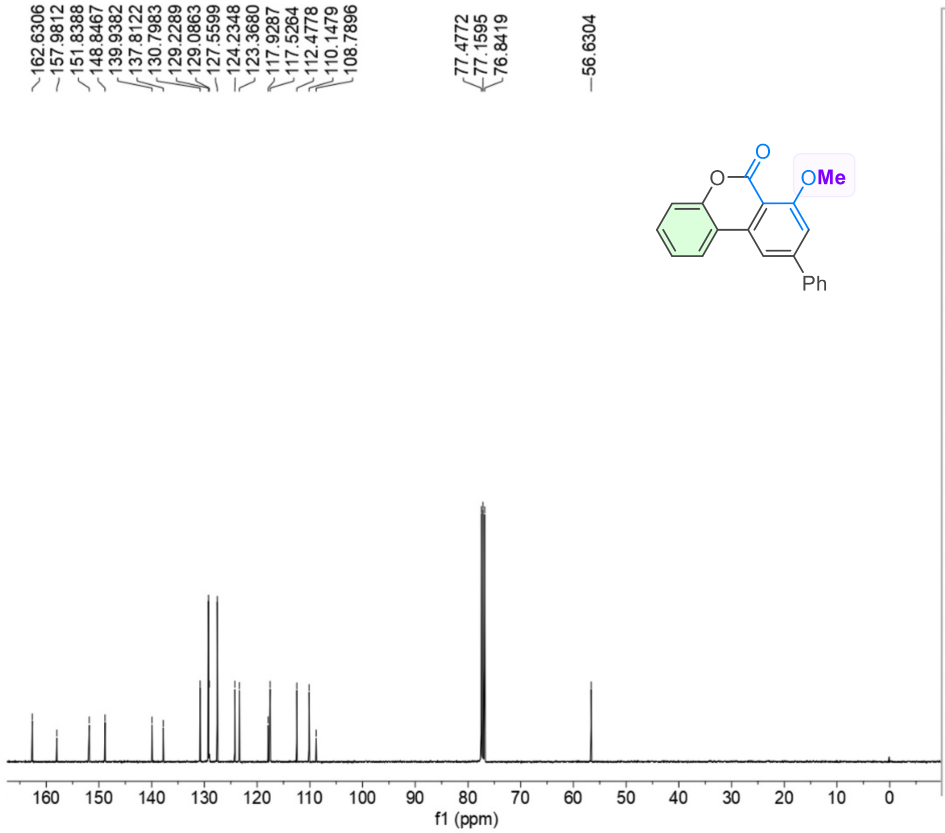

Parameter	值
1 Data File Name	E:/ 23-1-hxw-C/ 29/ fid
2 标题	23-1-hxw-C.29.fid
3 Comment	
4 Origin	Bruker BioSpin GmbH
5 Owner	nmrsu
6 Site	
7 Instrument	Avance NEO 400
8 Author	
9 Solvent	CDCl3
10 Temperature	295.7
11 Pulse Sequence	zgpg30
12 Experiment	1D
13 Probe	Z163739_0511 (PI HR-BBO400S1-BBF/ H/ D-5.0-Z SP)
14 Number of Scans	1024
15 Receiver Gain	16.2
16 Relaxation Delay	2.0000
17 Pulse Width	8.0000
18 Presaturation Frequency	
19 Acquisition Time	1.3763
20 Acquisition Date	2023-03-04T00:23:17
21 Modification Date	2023-03-04T00:22:24
22 Class	
23 Spectrometer Frequency	100.63
24 Spectral Width	23809.5
25 Lowest Frequency	-1834.1
26 Nucleus	13C
27 Acquired Size	32768
28 Spectral Size	65536

7-(Benzyloxy)-9-phenyl-6*H*-benzo[*c*]chromen-6-one (product 8)

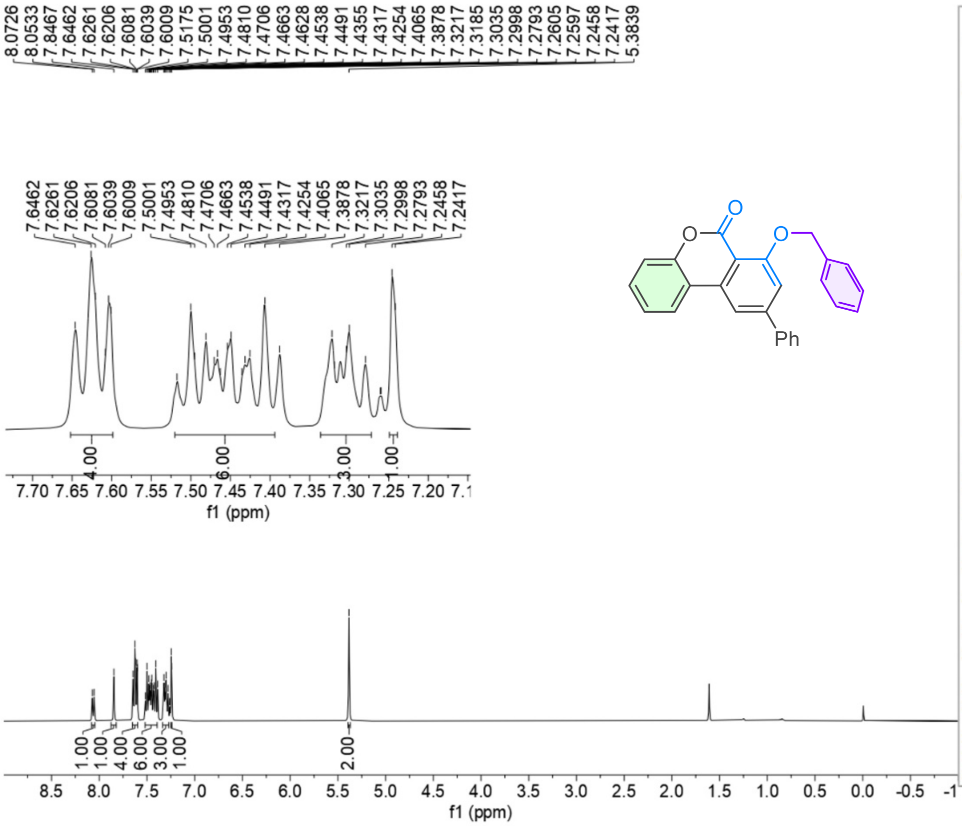

	Parameter	值
1	Data File Name	E:/ hxw2023/ 23-1-hxw-H/ 100/ fid
2	标题	23-1-hxw-H. 100.fid
3	Comment	
4	Origin	Bruker BioSpin GmbH
5	Owner	nmrsu
6	Site	
7	Instrument	Avance NEO 400
8	Author	
9	Solvent	CDCl3
10	Temperature	295.1
11	Pulse Sequence	zg30
12	Experiment	1D
13	Probe	Z163739_0511 (PI HR-BBO400S1-BBF/ H/ D-5.0-Z SP)
14	Number of Scans	4
15	Receiver Gain	101.0
16	Relaxation Delay	1.0000
17	Pulse Width	8.0000
18	Presaturation Frequency	
19	Acquisition Time	3.9977
20	Acquisition Date	2023-04-05T17:24:47
21	Modification Date	2023-04-05T17:24:52
22	Class	
23	Spectrometer Frequency	400.15
24	Spectral Width	8196.7
25	Lowest Frequency	-1642.6
26	Nucleus	1H
27	Acquired Size	32768
28	Spectral Size	65536

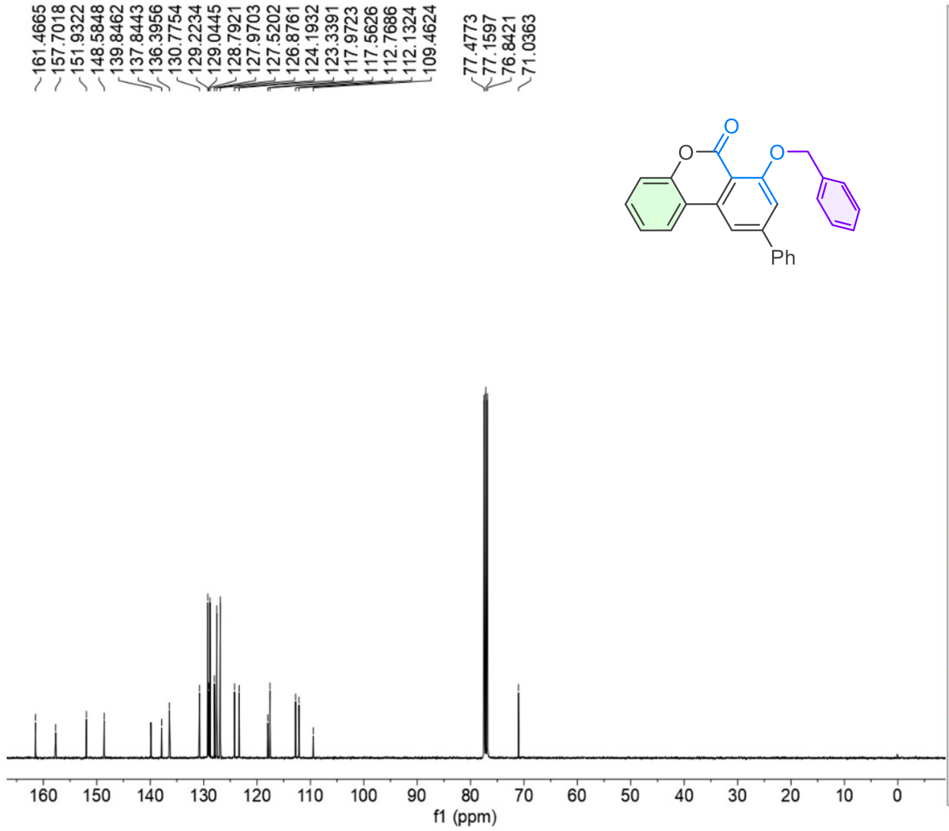

	Parameter	值
1	Data File Name	E:/ hxw2023/ 23-1-hxw-C/ 85/ fid
2	标题	23-1-hxw-C. 85.fid
3	Comment	
4	Origin	Bruker BioSpin GmbH
5	Owner	nmrsu
6	Site	
7	Instrument	Avance NEO 400
8	Author	
9	Solvent	CDCl3
10	Temperature	295.7
11	Pulse Sequence	zgpg30
12	Experiment	1D
13	Probe	Z163739_0511 (PI HR-BBO400S1-BBF/ H/ D-5.0-Z SP)
14	Number of Scans	1000
15	Receiver Gain	16.2
16	Relaxation Delay	2.0000
17	Pulse Width	8.0000
18	Presaturation Frequency	
19	Acquisition Time	1.3763
20	Acquisition Date	2023-04-05T18:36:31
21	Modification Date	2023-04-05T18:36:40
22	Class	
23	Spectrometer Frequency	100.63
24	Spectral Width	23809.5
25	Lowest Frequency	-1833.7
26	Nucleus	13C
27	Acquired Size	32768
28	Spectral Size	65536

6-Oxo-9-phenyl-6*H*-benzo[*c*]chromen-7-yl trifluoromethanesulfonate (product 9)

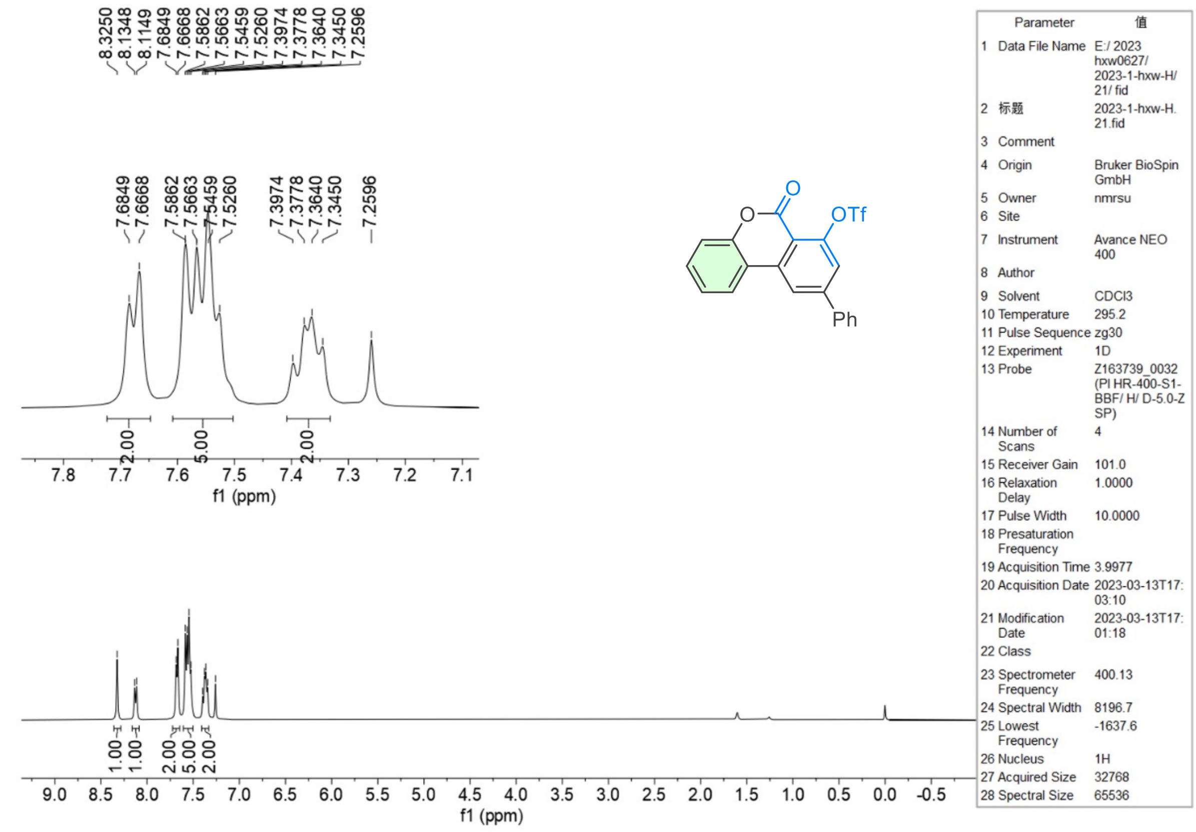

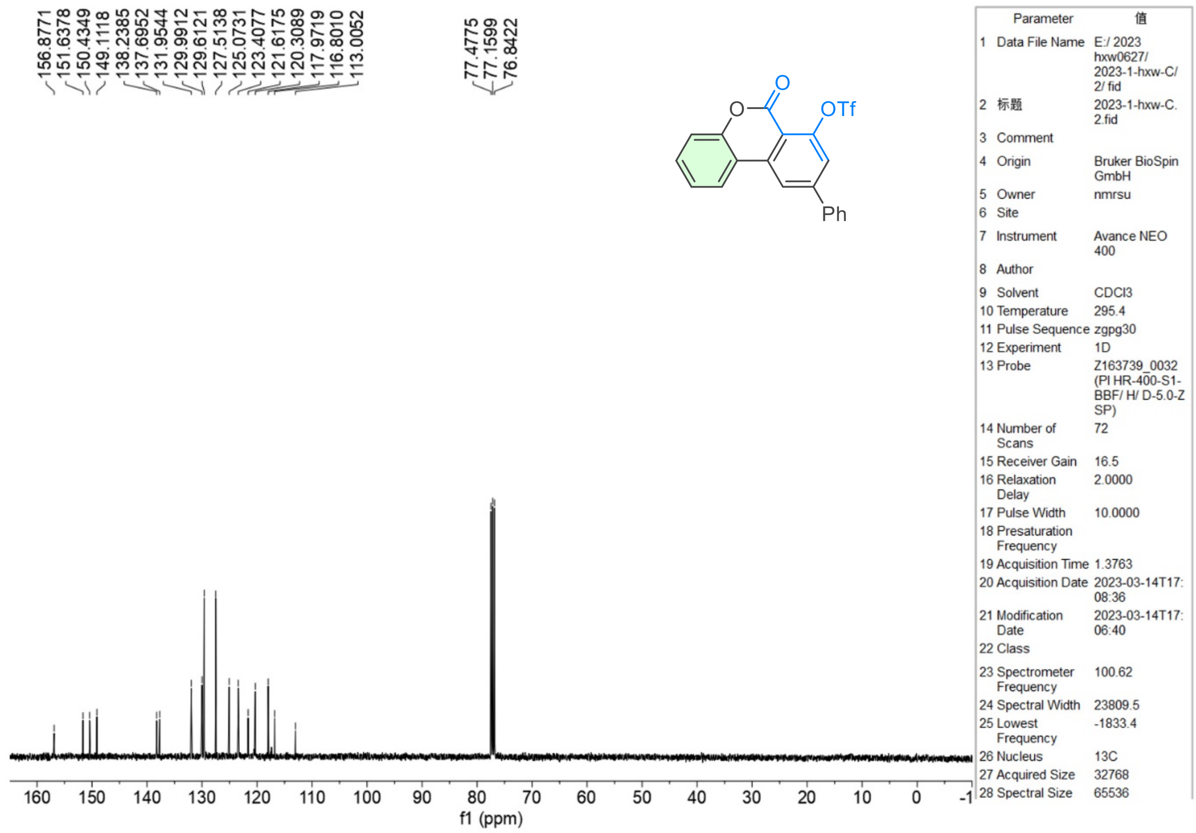

6-Oxo-9-phenyl-6*H*-benzo[*c*]chromen-7-yl trifluoromethanesulfonate (product 9)

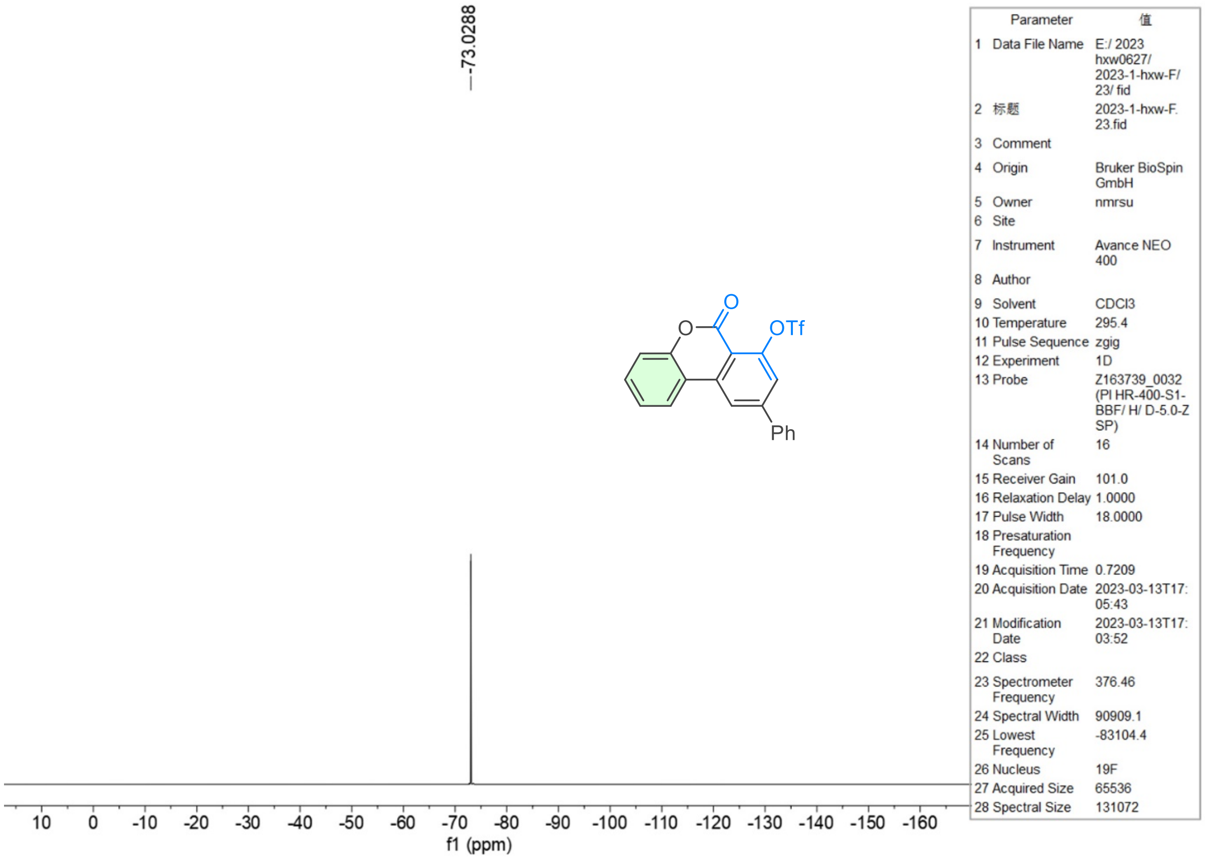

	Parameter	值
1	Data File Name	E:/ 2023 hxw0627/ 2023-1-hxw-F/ 23/ fid
2	标题	2023-1-hxw-F. 23.fid
3	Comment	
4	Origin	Bruker BioSpin GmbH
5	Owner	nmrsu
6	Site	
7	Instrument	Avance NEO 400
8	Author	
9	Solvent	CDCl3
10	Temperature	295.4
11	Pulse Sequence	zgig
12	Experiment	1D
13	Probe	Z163739_0032 (PI HR-400-S1-BBF/ H/ D-5.0-Z SP)
14	Number of Scans	16
15	Receiver Gain	101.0
16	Relaxation Delay	1.0000
17	Pulse Width	18.0000
18	Presaturation Frequency	
19	Acquisition Time	0.7209
20	Acquisition Date	2023-03-13T17:05:43
21	Modification Date	2023-03-13T17:03:52
22	Class	
23	Spectrometer Frequency	376.46
24	Spectral Width	90909.1
25	Lowest Frequency	-83104.4
26	Nucleus	19F
27	Acquired Size	65536
28	Spectral Size	131072

7-(Diphenylphosphoryl)-9-phenyl-6*H*-benzo[*c*]chromen-6-one (product 10)

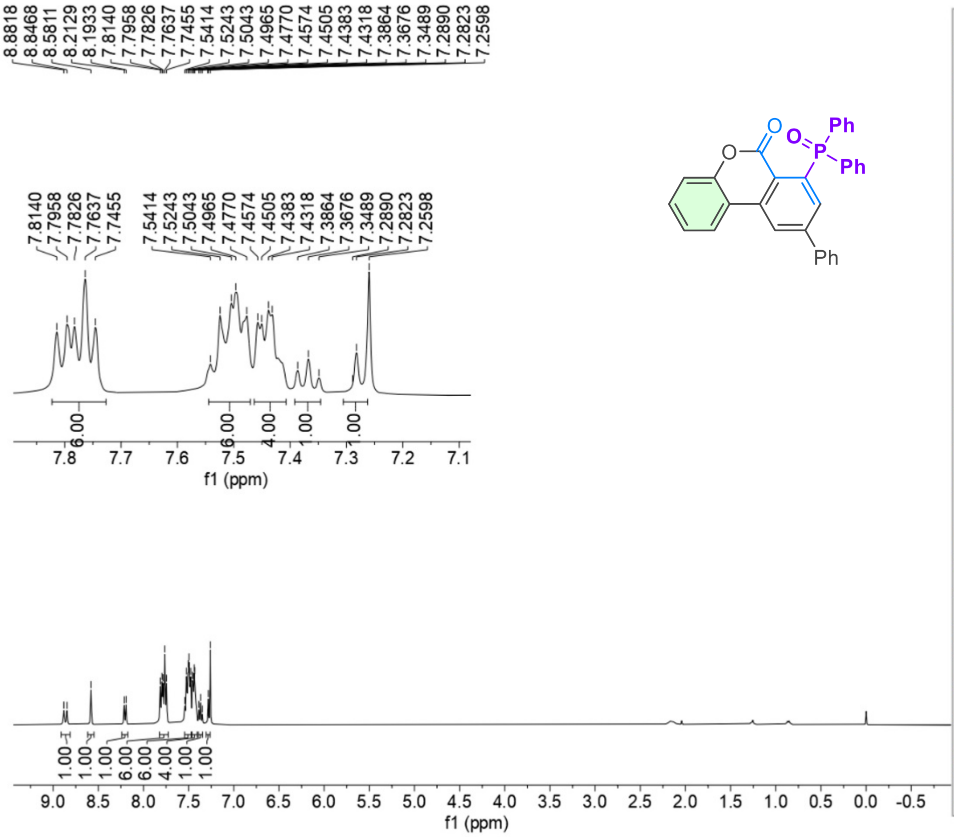

Parameter	值
1 Data File Name	E:/ 2023 hxw0627/ 23-1-hxw-H/ 94/ fid
2 标题	23-1-hxw-H.94.fid
3 Comment	
4 Origin	Bruker BioSpin GmbH
5 Owner	nmrsu
6 Site	
7 Instrument	Avance NEO 400
8 Author	
9 Solvent	CDCl3
10 Temperature	295.0
11 Pulse Sequence	zg30
12 Experiment	1D
13 Probe	Z163739_0511 (PI HR-BBO400S1-BBF/ H/ D-5.0-Z SP)
14 Number of Scans	4
15 Receiver Gain	101.0
16 Relaxation Delay	1.0000
17 Pulse Width	8.0000
18 Presaturation Frequency	
19 Acquisition Time	3.9977
20 Acquisition Date	2023-03-29T21:09:56
21 Modification Date	2023-03-29T21:09:04
22 Class	
23 Spectrometer Frequency	400.15
24 Spectral Width	8196.7
25 Lowest Frequency	-1637.4
26 Nucleus	1H
27 Acquired Size	32768
28 Spectral Size	65536

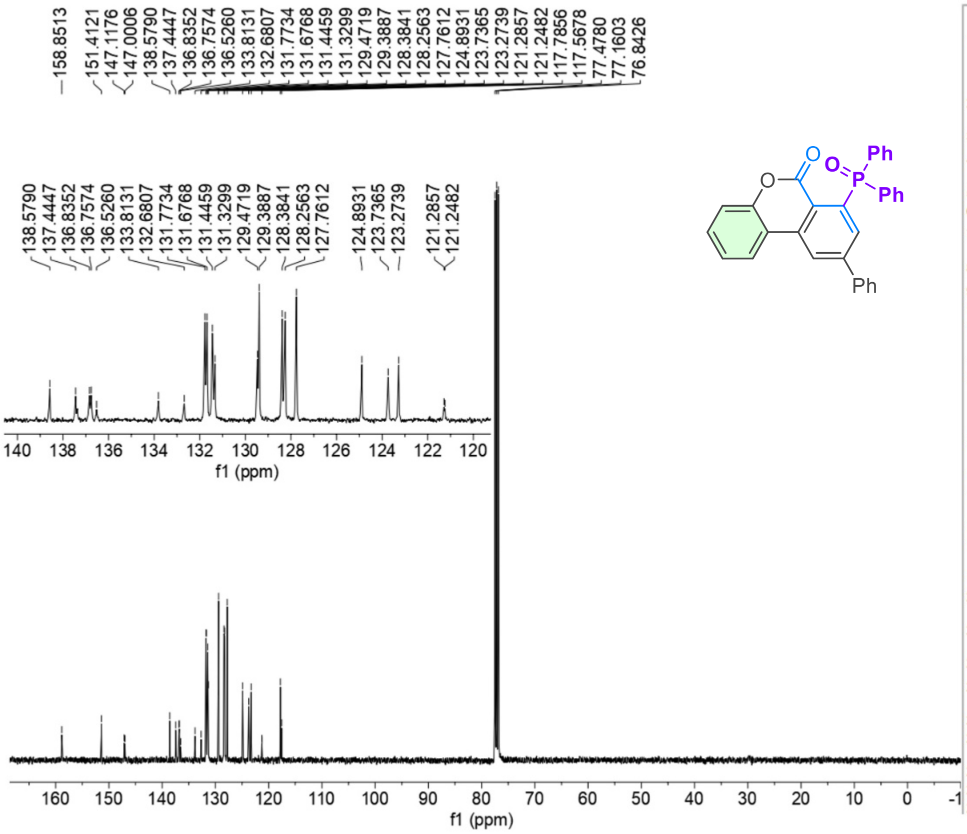

Parameter	值
1 Data File Name	E:/ 2023 hxw0627/ 2023-1-hxw-C/ 5/ fid
2 标题	2023-1-hxw-C. 5.fid
3 Comment	
4 Origin	Bruker BioSpin GmbH
5 Owner	nmrsu
6 Site	
7 Instrument	Avance NEO 400
8 Author	
9 Solvent	CDCl3
10 Temperature	295.5
11 Pulse Sequence	zgpg30
12 Experiment	1D
13 Probe	Z163739_0032 (PI HR-400-S1-BBF/ H/ D-5.0-Z SP)
14 Number of Scans	1500
15 Receiver Gain	16.5
16 Relaxation Delay	2.0000
17 Pulse Width	10.0000
18 Presaturation Frequency	
19 Acquisition Time	1.3763
20 Acquisition Date	2023-04-01T02:36:20
21 Modification Date	2023-04-01T02:35:28
22 Class	
23 Spectrometer Frequency	100.62
24 Spectral Width	23809.5
25 Lowest Frequency	-1834.1
26 Nucleus	13C
27 Acquired Size	32768
28 Spectral Size	65536

7-(Diphenylphosphoryl)-9-phenyl-6*H*-benzo[*c*]chromen-6-one (product 10)

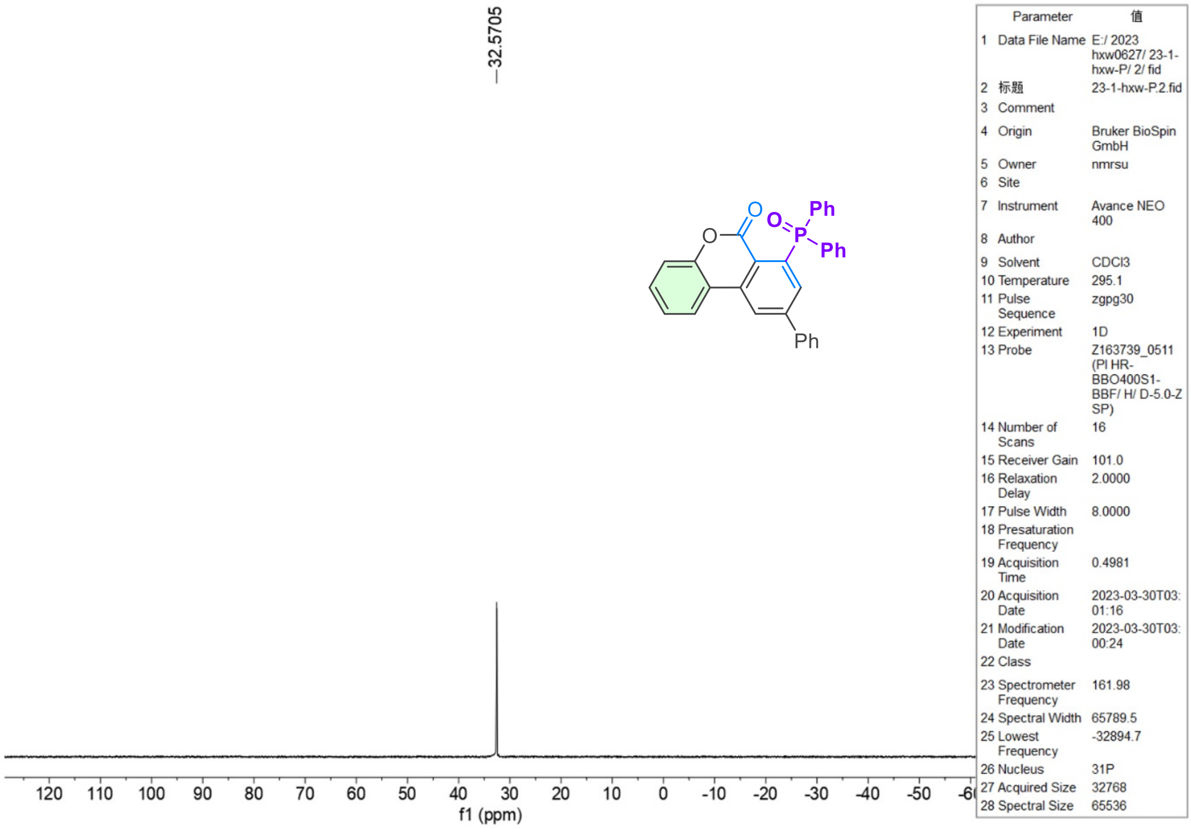

7-(4-(Diphenylamino)phenyl)-9-phenyl-6*H*-benzo[*c*]chromen-6-one (product 11)

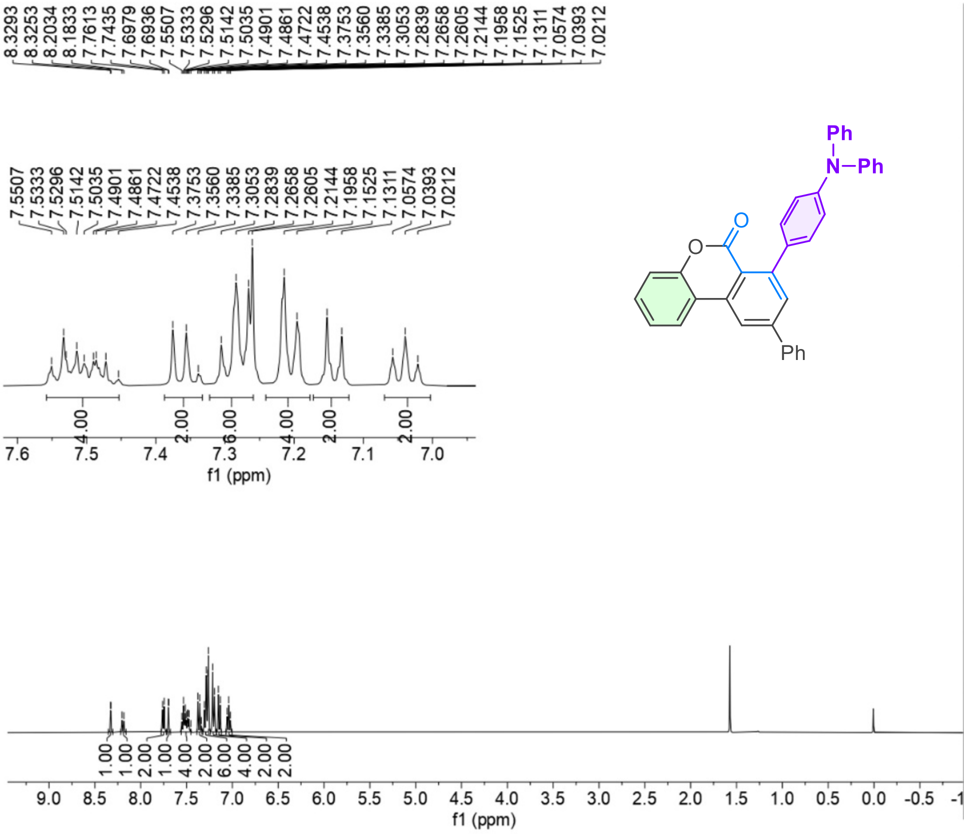

Parameter	值
1 Data File Name	E:/ 2023 hxw0627/ 2023-1-hxw-H/ 39/ fid
2 标题	2023-1-hxw-H. 39.fid
3 Comment	
4 Origin	Bruker BioSpin GmbH
5 Owner	nmrsu
6 Site	
7 Instrument	Avance NEO 400
8 Author	
9 Solvent	CDCl3
10 Temperature	294.9
11 Pulse Sequence	zg30
12 Experiment	1D
13 Probe	Z163739_0032 (PI HR-400-S1-BBF/ H/ D-5.0-Z SP)
14 Number of Scans	4
15 Receiver Gain	101.0
16 Relaxation Delay	1.0000
17 Pulse Width	10.0000
18 Presaturation Frequency	
19 Acquisition Time	3.9977
20 Acquisition Date	2023-03-31T21:37:21
21 Modification Date	2023-03-31T21:36:30
22 Class	
23 Spectrometer Frequency	400.13
24 Spectral Width	8196.7
25 Lowest Frequency	-1637.2
26 Nucleus	1H
27 Acquired Size	32768
28 Spectral Size	65536

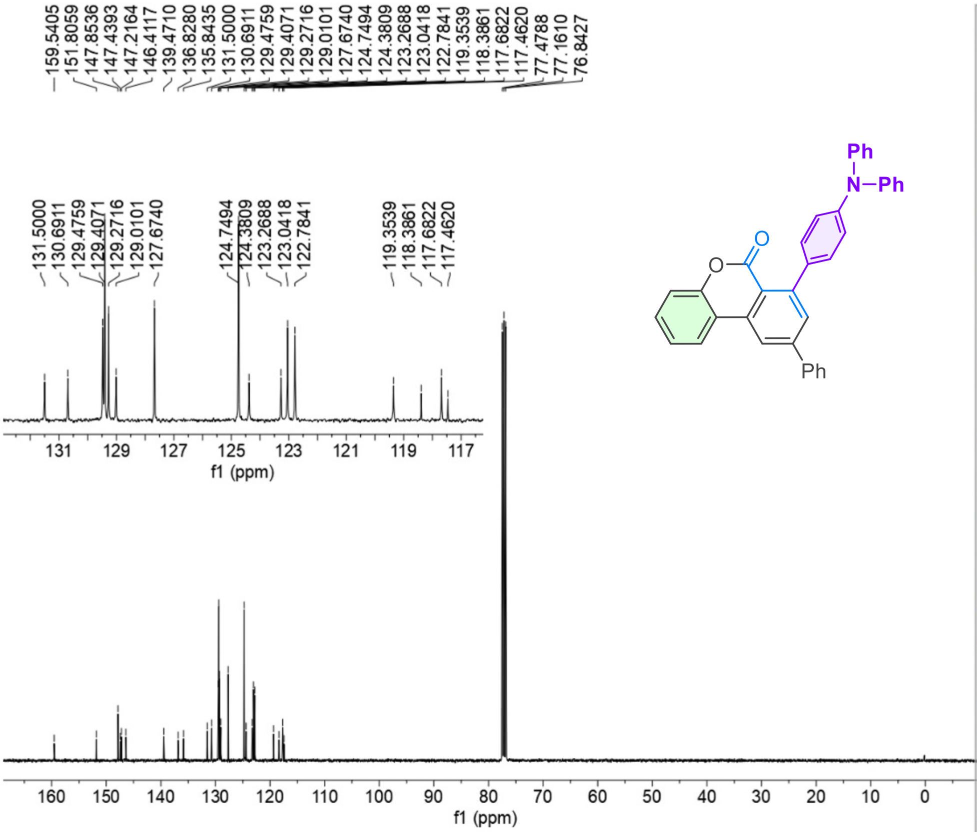

Parameter	值
1 Data File Name	E:/ 2023 hxw0627/ 2023-1-hxw-C/ 6/ fid
2 标题	2023-1-hxw-C. 6.fid
3 Comment	
4 Origin	Bruker BioSpin GmbH
5 Owner	nmrsu
6 Site	
7 Instrument	Avance NEO 400
8 Author	
9 Solvent	CDCl3
10 Temperature	295.5
11 Pulse Sequence	zgpg30
12 Experiment	1D
13 Probe	Z163739_0032 (PI HR-400-S1-BBF/ H/ D-5.0-Z SP)
14 Number of Scans	1024
15 Receiver Gain	15.5
16 Relaxation Delay	2.0000
17 Pulse Width	10.0000
18 Presaturation Frequency	
19 Acquisition Time	1.3763
20 Acquisition Date	2023-04-01T03:45:52
21 Modification Date	2023-04-01T03:45:00
22 Class	
23 Spectrometer Frequency	100.62
24 Spectral Width	23809.5
25 Lowest Frequency	-1820.1
26 Nucleus	13C
27 Acquired Size	32768
28 Spectral Size	65536

6,6-Dimethyl-9-phenyl-6*H*-benzo[*c*]chromen-7-yl trifluoromethanesulfonate (product 12)

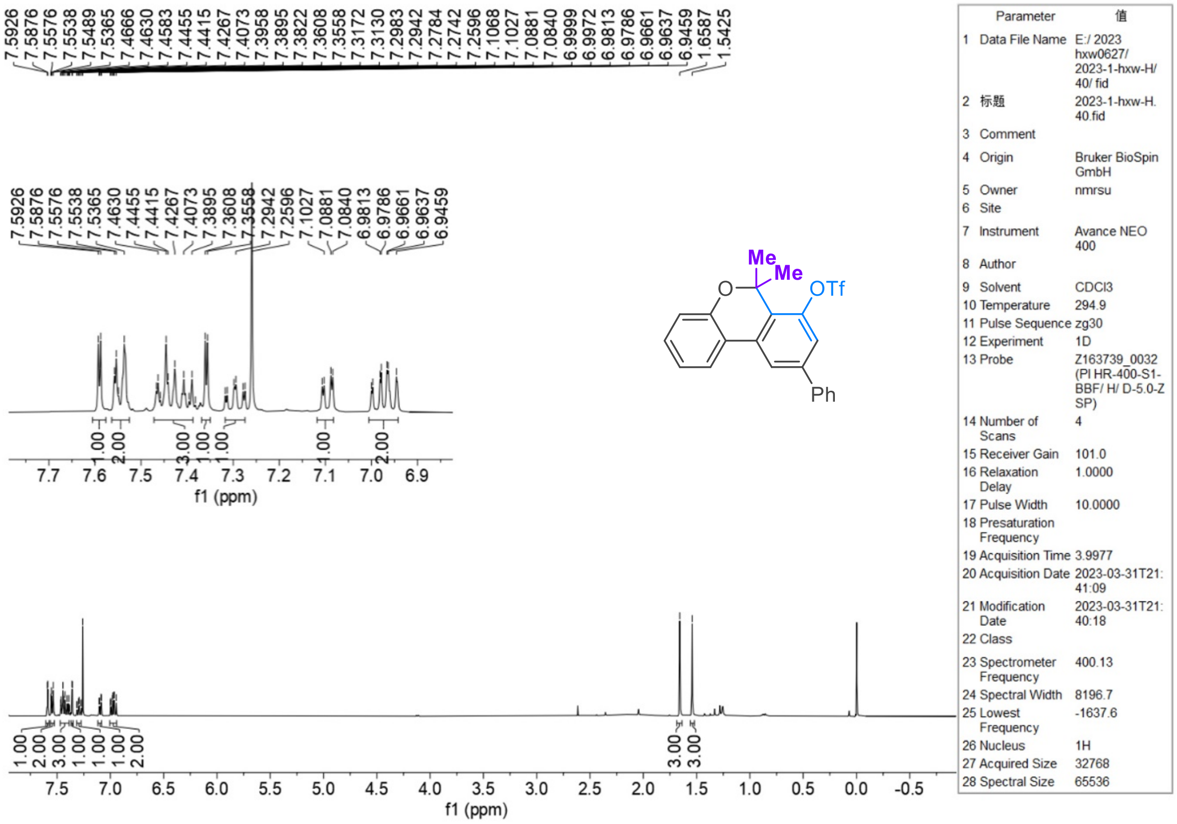

	Parameter	值
1	Data File Name	E:/ 2023 hxw0627/ 2023-1-hxw-H/ 40/ fid
2	标题	2023-1-hxw-H. 40.fid
3	Comment	
4	Origin	Bruker BioSpin GmbH
5	Owner	nmrsu
6	Site	
7	Instrument	Avance NEO 400
8	Author	
9	Solvent	CDCl3
10	Temperature	294.9
11	Pulse Sequence	zg30
12	Experiment	1D
13	Probe	Z163739_0032 (PI HR-400-S1-BBF/ H/ D-5.0-Z SP)
14	Number of Scans	4
15	Receiver Gain	101.0
16	Relaxation Delay	1.0000
17	Pulse Width	10.0000
18	Presaturation Frequency	
19	Acquisition Time	3.9977
20	Acquisition Date	2023-03-31T21:41:09
21	Modification Date	2023-03-31T21:40:18
22	Class	
23	Spectrometer Frequency	400.13
24	Spectral Width	8196.7
25	Lowest Frequency	-1637.6
26	Nucleus	1H
27	Acquired Size	32768
28	Spectral Size	65536

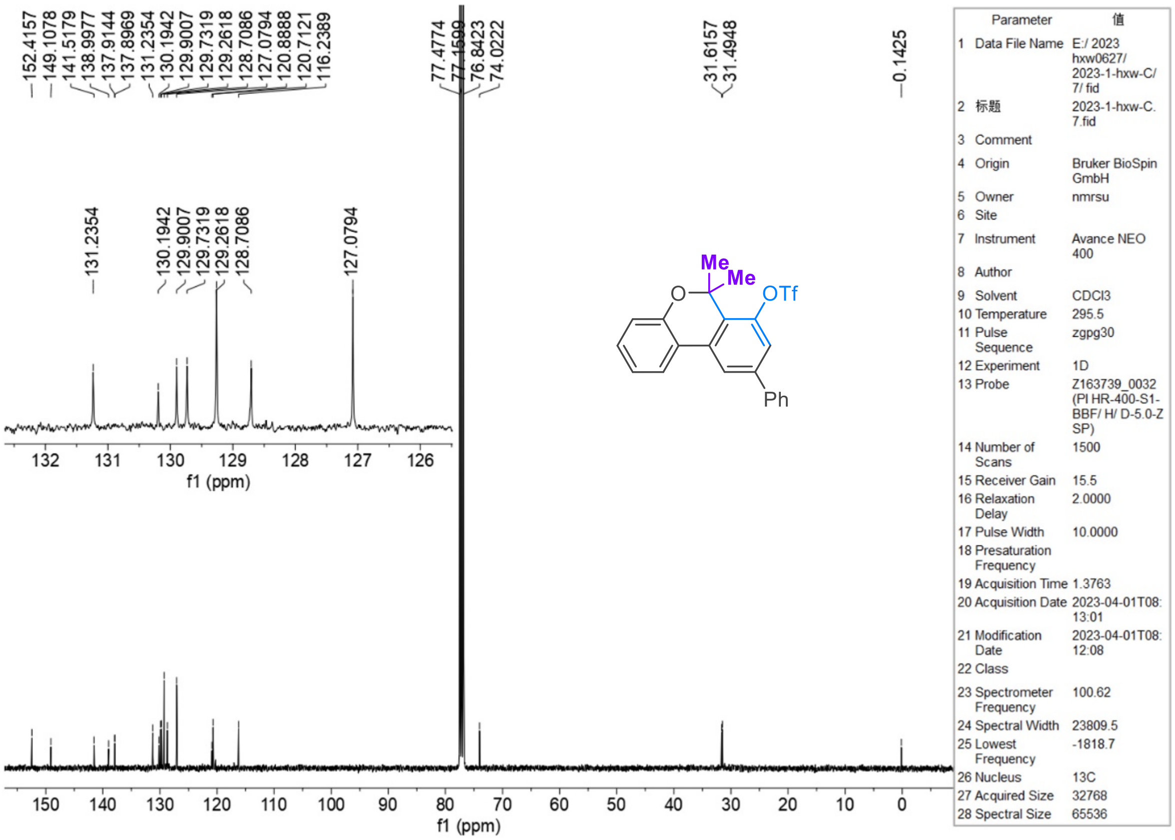

	Parameter	值
1	Data File Name	E:/ 2023 hxw0627/ 2023-1-hxw-C/ 7/ fid
2	标题	2023-1-hxw-C. 7.fid
3	Comment	
4	Origin	Bruker BioSpin GmbH
5	Owner	nmrsu
6	Site	
7	Instrument	Avance NEO 400
8	Author	
9	Solvent	CDCl3
10	Temperature	295.5
11	Pulse Sequence	zgpg30
12	Experiment	1D
13	Probe	Z163739_0032 (PI HR-400-S1-BBF/ H/ D-5.0-Z SP)
14	Number of Scans	1500
15	Receiver Gain	15.5
16	Relaxation Delay	2.0000
17	Pulse Width	10.0000
18	Presaturation Frequency	
19	Acquisition Time	1.3763
20	Acquisition Date	2023-04-01T08:13:01
21	Modification Date	2023-04-01T08:12:08
22	Class	
23	Spectrometer Frequency	100.62
24	Spectral Width	23809.5
25	Lowest Frequency	-1818.7
26	Nucleus	13C
27	Acquired Size	32768
28	Spectral Size	65536

6,6-Dimethyl-9-phenyl-6*H*-benzo[*c*]chromen-7-yl trifluoromethanesulfonate (product 12)

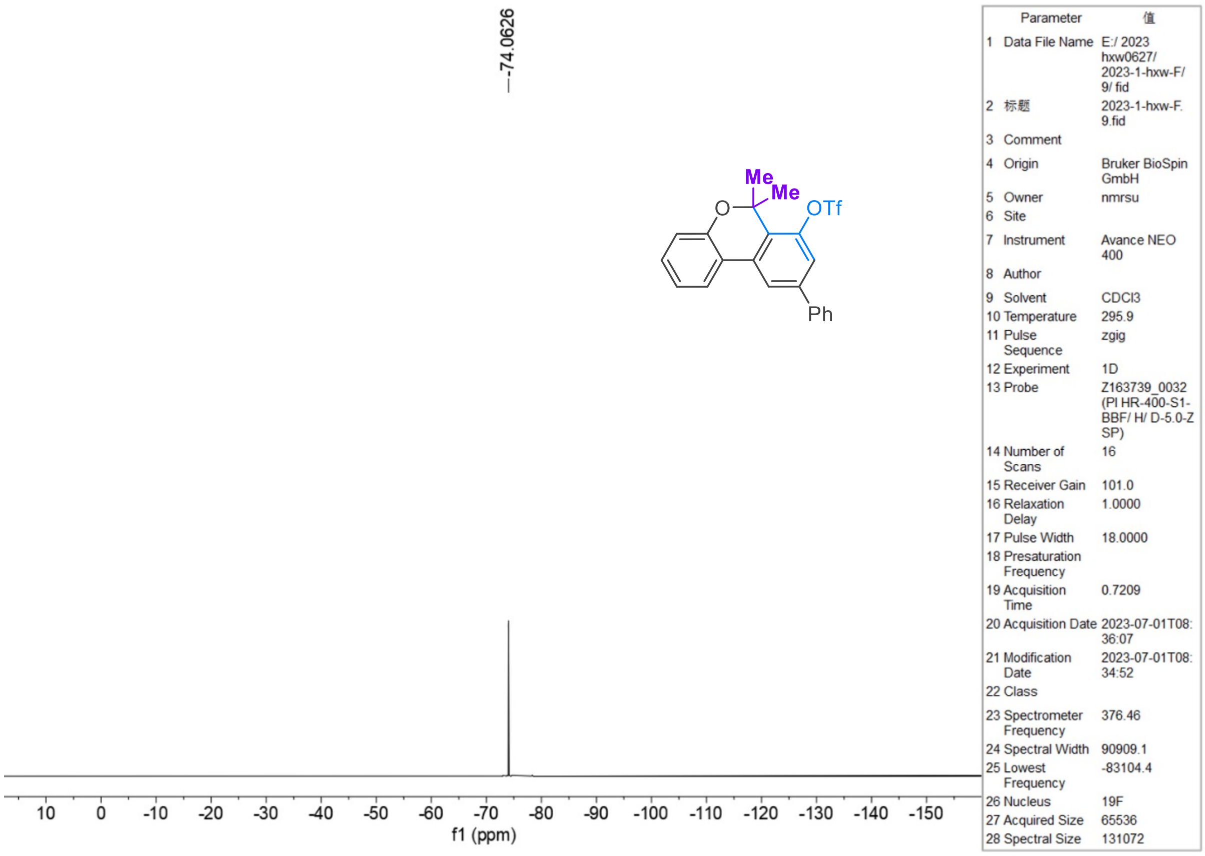

	Parameter	值
1	Data File Name	E:/ 2023 hxw0627/ 2023-1-hxw-F/ 9/ fid
2	标题	2023-1-hxw-F. 9.fid
3	Comment	
4	Origin	Bruker BioSpin GmbH
5	Owner	nmrsu
6	Site	
7	Instrument	Avance NEO 400
8	Author	
9	Solvent	CDCl3
10	Temperature	295.9
11	Pulse Sequence	zgig
12	Experiment	1D
13	Probe	Z163739_0032 (PI HR-400-S1-BBF/ H/ D-5.0-Z SP)
14	Number of Scans	16
15	Receiver Gain	101.0
16	Relaxation Delay	1.0000
17	Pulse Width	18.0000
18	Presaturation Frequency	
19	Acquisition Time	0.7209
20	Acquisition Date	2023-07-01T08:36:07
21	Modification Date	2023-07-01T08:34:52
22	Class	
23	Spectrometer Frequency	376.46
24	Spectral Width	90909.1
25	Lowest Frequency	-83104.4
26	Nucleus	19F
27	Acquired Size	65536
28	Spectral Size	131072

7-Hydroxy-9-phenyl-2-(phenylethynyl)-6*H*-benzo[*c*]chromen-6-one (product 13)

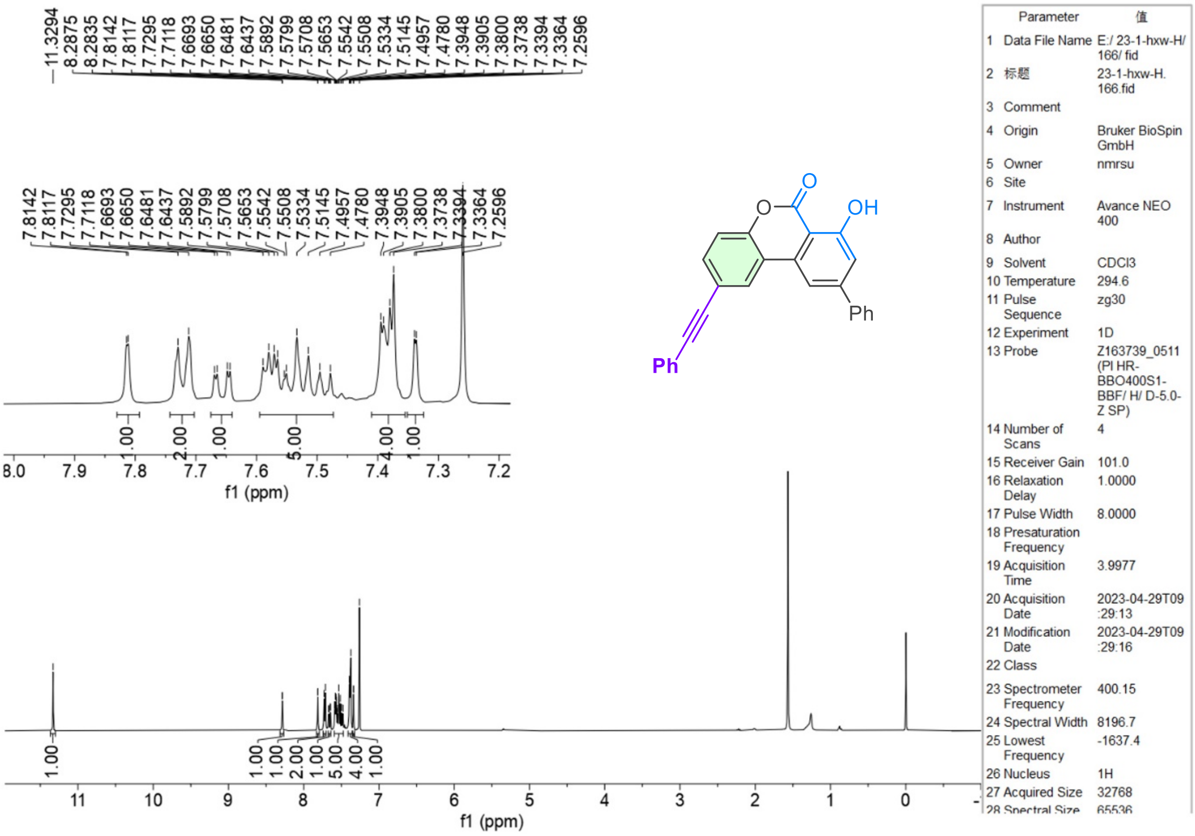

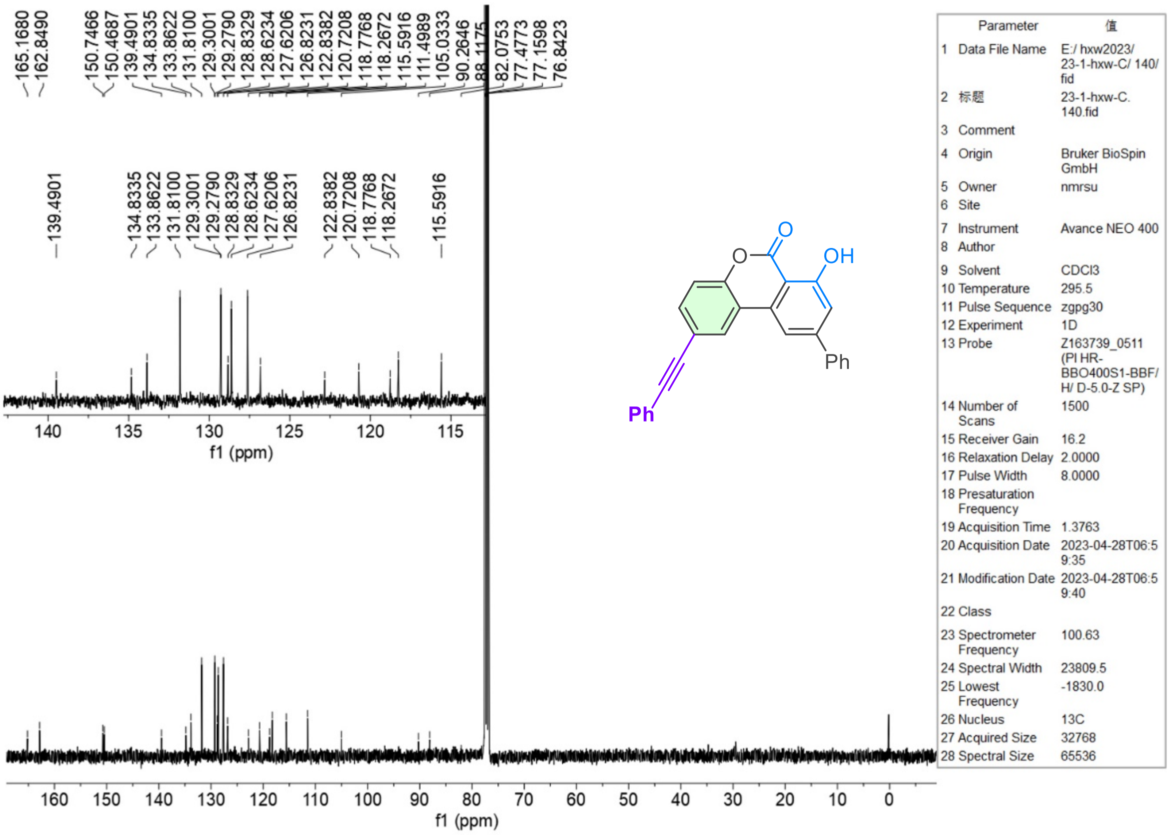

Supplement: Supplementary file 1 — jo3c02188_si_001.pdf [file jo3c02188_si_001.pdf]
